# Photoredox-Catalyzed Synthesis of 3-Sulfonylated Pyrrolin-2-ones via a Regioselective Tandem Sulfonylation Cyclization of 1,5-Dienes

**DOI:** 10.3390/molecules28145473

**Published:** 2023-07-17

**Authors:** Ran Ding, Liang Li, Ya-Ting Yu, Bing Zhang, Pei-Long Wang

**Affiliations:** 1 College of Chemistry and Materials Engineering, Anhui Science and Technology University, Bengbu 233100, China; ll15855863179@126.com (L.L.); yyt2747196669@126.com (Y.-T.Y.); zhangbing2168@126.com (B.Z.); 2 Key Laboratory of Green and Precise Synthetic Chemistry and Applications, Ministry of Education, School of Chemistry and Materials Science, Huaibei Normal University, Huaibei 235000, China; 3 Information College, Huaibei Normal University, Huaibei 235000, China

**Keywords:** regioselective, dienes, radical, cyclization, pyrrolin-2-ones

## Abstract

A mild, visible-light-induced, regioselective cascade sulfonylation-cyclization of 1,5-dienes with sulfonyl chlorides through the intermolecular radical addition/cyclization of alkenes C(sp^2^)-H was developed. This procedure proceeds well and affords a mild and efficient route to a range of monosulfonylated pyrrolin-2-ones at room temperatures.

## 1. Introduction

**Figure 1:**
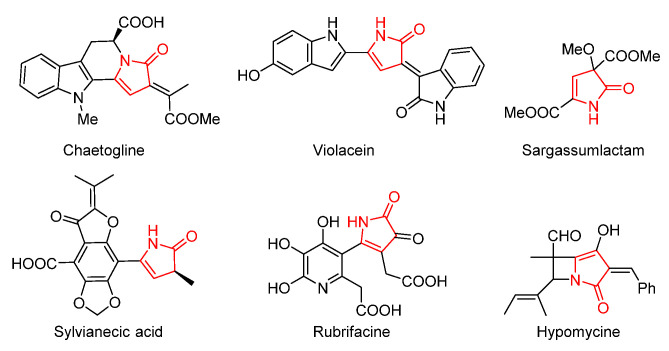
Examples of compounds containing pyrrolin-2-ones.

Pyrrolin-2-ones, which constitute one of the most prominent classes of skeletons exhibiting unique biological activities, are prevalent in a large number of biological pharmaceutical molecules [1,2] and natural products, like chaetogline, violacein, and hypomycine [3,4,5,6] (Figure 1). In this context, considerable effort has been focused in establishing such valuable frameworks, but most of these methods suffer from transition metals or harsh reaction conditions [7,8,9,10,11,12]. Therefore, developing general and effective synthetic methods for pyrrolin-2-ones and its derivatives with mild conditions has been attracting increasing attention and largely promote progress in this area [13,14,15,16,17,18]. On the other hand, the photoinduced radical cascade cyclization reaction has become a powerful tool to construct *N*-containing heterocycles because of its extremely high efficiency, inherently green, infinite availability, safety, and ease of operation [19,20,21,22,23,24]. However, such an efficient strategy for the synthesis of pyrrolin-2-ones has rarely been reported [25].

**Scheme 1:**
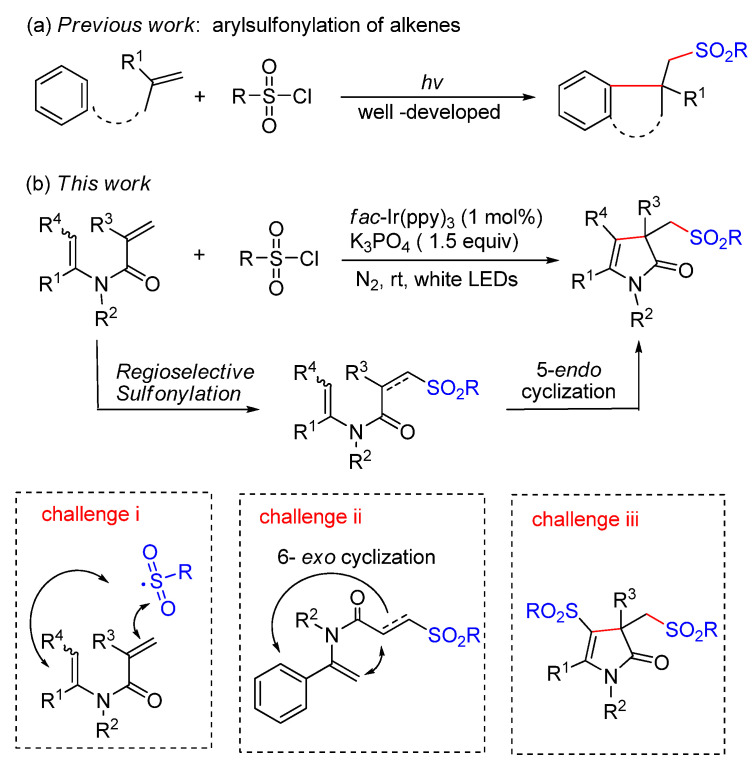
Radical cyclization of tethered alkenes. Challenge i: Regioselectivity of sulfone radical addition; Challenge ii: Two pathway of cyclization; Challenge iii: 3,4-disulfonated pyrrolin-2-ones.

Sulfones constitute an important class of functional groups in organic synthesis that can participate in various chemical transformations [26,27] and that are found widely in the structures of natural products [28,29,30]. The introduction of sulfonyl functional groups can cause molecules to exhibit unique biological activity [31,32]. In this regard, a considerable amount of effort has been devoted to the development of efficient, simple, and convenient methods for synthesizing sulfonyl-containing compounds [33,34,35,36,37,38]. Among the many approaches, the difunctionalization of alkenes through a radical process has been used to prepare several sulfone-containing compounds [39,40,41,42,43,44,45,46,47,48]. Sulfonyl chloride is a readily available and easily handled source of the sulfonyl moiety and is commonly used to generate sulfonyl radicals under visible light conditions; major advances have focused on reactions with heteroaryl or aryl-tethered alkenes to produce sulfonyl-containing aromatic compounds (Scheme 1a) [49,50,51,52,53,54,55]. Nevertheless, the reactions of vinyl-tethered alkenes remain elusive [56,57].

Considering the significance of pyrrolinones and the importance of sulfone moieties in organic synthesis. Herein, we aimed to develop an unprecedented visible-light-induced photoredox-catalyzed reaction of linear 1,5-dienes with sulfonyl chlorides via regioselective sulfonylation and 5-endo cyclization to produce important pyrrolinones (Scheme 1b). However, three challenges hinder the successful development of such a process: (i) The selective addition of the sulfone radical between two carbon-carbon double bonds is challenging. (ii) 6-Exo cyclization competes with the desired reaction and needs to be restricted. (iii) The C=C bond in the target product continues to react with the sulfonyl radical to afford 3,4-disulfonated pyrrolin-2-ones.

We then focused on the reaction of *N*-acetyl-*N*-(1-phenylvinyl)methacrylamide **1a** and *p*-toluenesulfonyl chloride **2a**. To our delight, when the reaction was performed in the presence of a catalytic amount of *fac*-Ir(ppy)_3_ and equivalent of Na_2_CO_3_ in CH_2_Cl_2_ under irradiation with 20 W white LEDs (Light-Emitting Diodes) for 16 h, the target sulfonylated pyrrolinone **3a** could be isolated in 57% yield (Table 1, entry 1). Subsequently, other photocatalysts, such as Ru(bpy)_3_Cl_2_ and eosin Y, were investigated, but all failed to obtain product **3a** (entries 2, 3). After examining various bases, such as Li_2_CO_3_ (59%), NaHCO_3_ (64%), K_3_PO_4_ (72%), and Na_3_PO_4_ (63%), K_3_PO_4_ was determined to be the best base (entries 4–8). A variety of solvents, including DCE (1,2-dichloroethane), CHCl_3_, acetone, toluene, THF (tetrahydrofuran), and EtOAc, were subsequently screened, but the yield of product **3a** was not promoted (entries 9–14). Next, the amounts of K_3_PO_4_ were evaluated (entries 15, 16). Using 1.5 equiv. of K_3_PO_4_ improved the yield of product **3a** by 79%. When the light source was changed to 5 W white LEDs, product **3a** was afforded in the same yield as previously obtained (entries 15 vs. 17). The results of the control experiments showed that visible light, photocatalyst [*fac*-Ir(ppy)_3_], and base K_3_PO_4_ were necessary for this reaction (entries 18–20).

## 2. Results and Discussion

**Figure 2:**
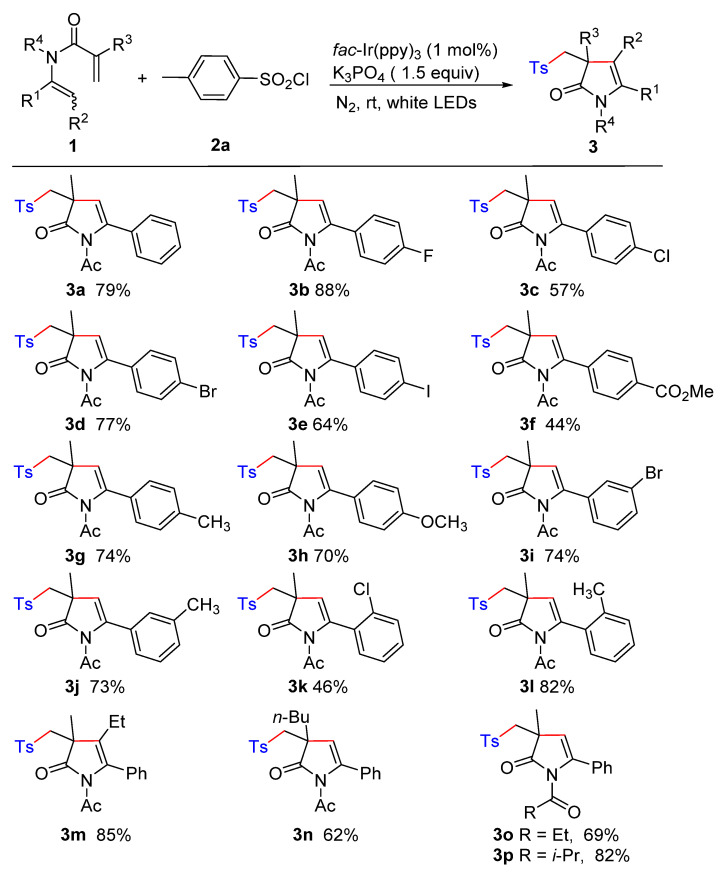
Substrate Scope of 1,5-dienes. Reaction conditions: **1** (0.1 mmol), **2a** (0.2 mmol), and Ir(ppy)_3_ (1 mol%) in CH_2_Cl_2_ (1 mL) were irradiated with 5 W white LEDs at room temperature under N_2_ for 16 h. The yields were isolated yields.

After obtaining the optimal reaction conditions, we embarked upon exploring the substrate scope of 1,5-dienes. Different R^1^, R^2^, R^3^ and R^4^ groups of 1,5-dienes were tested with p-toluenesulfonyl chloride **2a**; the results are shown in Figure 2. Substrates with halogen atoms (F, Cl, Br, and I) and electron-donating groups (Me and MeO) at the para-positions of the benzene ring proceeded well to give target products **3b**–**3g** and **3g**–**3h** in medium to good yields. Gratifyingly, the CO_2_Et group at the para-position of the benzene ring furnished product **3f** in an acceptable yield. The reactivity of substituents at the meta- or ortho-position was also tested, achieving yields of products **3i**–**3l** from 46% to 82%. Notably, substrates with an ethyl group at the *β*-position of the enamide moiety or an *n*-butyl group at the *α*-position of the acrylamide moiety smoothly converted to the corresponding product **3m** or **3n** in 85% yield or 62% yield. In addition, using propionyl or isobutyryl as the nitrogen-protecting groups was viable for this reaction to give target products **3o** and **3p** in considerable yields.

**Figure 3:**
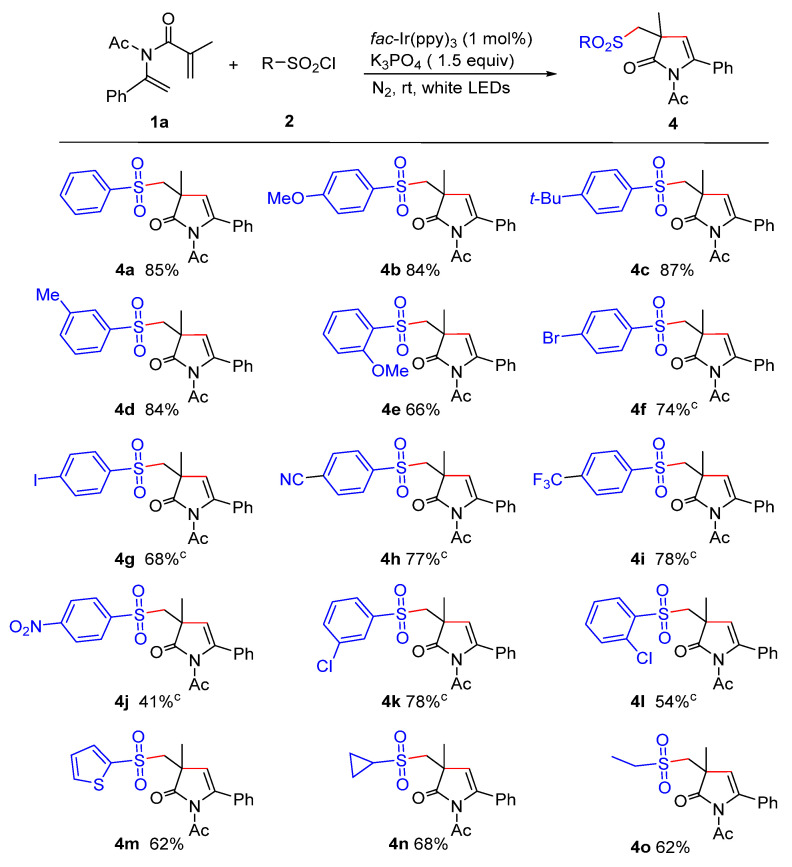
Substrate scope of sulfonyl chlorides. Reaction conditions: a mixture of **1a** (0.1 mmol), **2** (0.2 mmol), and Ir(ppy)_3_ (1 mol%) in CH_2_Cl_2_ (1 mL), which were irradiated with 5 W white LEDs at room temperature under N_2_ for 16 h. The yields were isolated yields. ^c^ 20 W white LEDs were used.

Next, we moved on to explore the generality of various sulfonyl chlorides (Figure 3). Arylsulfonyl chlorides bearing electron-rich (Me, MeO, and *t*-Bu) groups at different positions worked well, giving corresponding sulfones **4b**–**4e** in 66–84% yield. Electron-poor arylsulfonyl chlorides, such as Br, I, CN, CF_3_, and NO_2_ groups on the benzene ring, allowed the formation of product **4f**–**4j** in 41% to 78% yield with the need for 20 W white LEDs as the light source. It is noteworthy that arylsulfonyl chlorides having substituents at the ortho-position were inferior to those at the para- or meta-position, mainly because of the large steric hindrance of the ortho-position (**4b** vs. **4e** and **4k** vs. **4l**). Remarkably, 2-thiophenesulfonyl chloride survived under the current conditions to achieve product **4m** in 62% yield. Moreover, alkyl-substituted sulfonyl chlorides, such as cyclopropyl and ethyl, were applicable for this reaction and transferred to **4n** and **4o** in 68% and 62% yield, respectively.

**Scheme 2:**
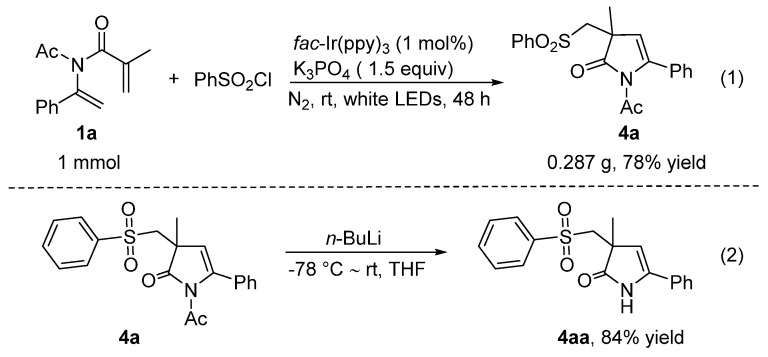
Gram−scale reaction and removal of OAc group. (1): Gram-scale reaction; (2): Removal of OAc group.

In order to further expand the practicality of the reaction, a gram scale reaction and removal of OAc group of compound **4a** were conducted. We were delighted to obtain the sulfonylated pyrrolinone **4a** in 78% yield with a prolonged time when the reaction was taken on 1 mmol scale (Scheme 2, (1)). Furthermore, with the addition of *n*-BuLi in THF at −78 °C, the compound **4a** could smoothly remove the OAc group, which generated the product **4aa** in 84% yield (Scheme 2, (2)).

**Scheme 3:**
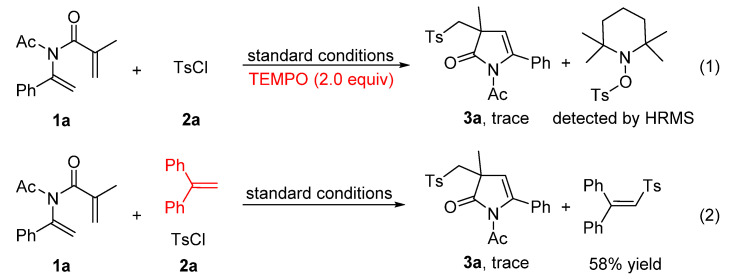
Mechanistic studies. (1): TEMPO (2.0 equiv.) was added; (2): 1,1-diphenylethylene (2.0 equiv.) was added.

To shed the possible mechanism of this visible-light-induced sulfonylation-cyclization of 1,5-dienes, some control experiments were carried out (Scheme 3). When 2.0 equivalents of TEMPO or 1,1-diphenylethylene was added to the reaction of 1,5-diene and *p*-toluenesulfonyl chloride under standard conditions, the transformation was completely suppressed, suggesting that a free-radical pathway may be involved in this sulfonylation-cyclization reaction. In addition, visible-light irradiation on/off experiments were performed on the model reaction, and the results show that a long-chain process was unlikely to be involved in this reaction (see Supplementary Materials).

**Scheme 4:**
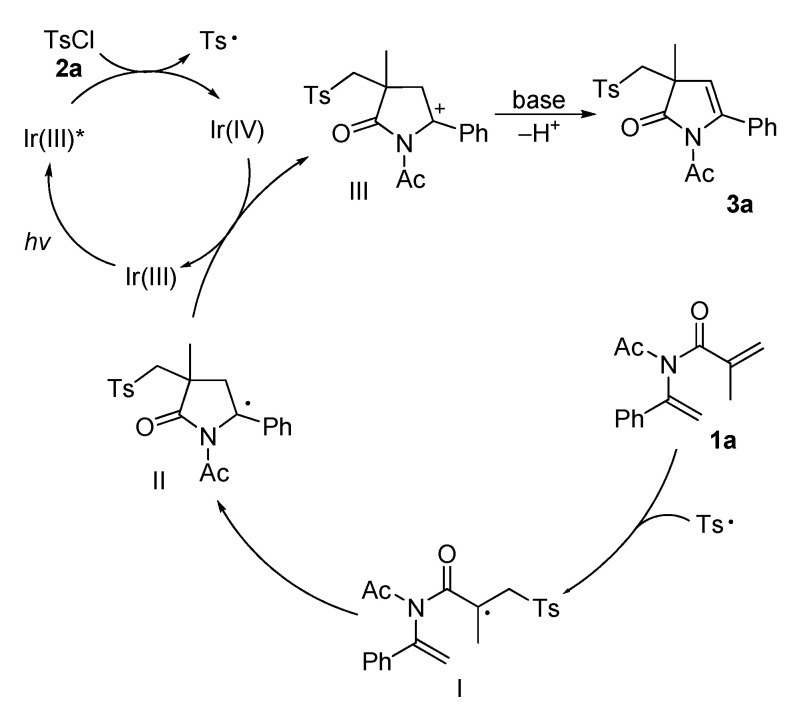
Proposed reaction mechanism.

According to the above experimental results and previous literature reports [13,14,15,16,17,18], we propose a possible mechanism for visible-light-induced regioselective cascade sulfonylation-cyclization of 1,5-dienes (Scheme 4). First, the photocatalyst [*fac*-Ir(ppy)_3_] under visible light irradiation is excited to form the strongly reducing state *[*fac*-Ir(ppy)_3_]. A single electron transfer between *[*fac*-Ir(ppy)_3_] and *p*-toluenesulfonyl chloride produces the *p*-toluenesulfonyl radical and oxidation state [*fac*-Ir(ppy)_3_]^+^. Second, the *p*-toluenesulfonyl radical was selectively added to the terminal carbon-carbon double bond of acrylamide of 1,5-diene, followed by a 5-endo cyclization to produce radical species II [58,59]. Although 5-endo cyclizations are often less favorable kinetically than their 4-*exo* cyclizations, the switch from 4-*exo* to 5-endo mode can be achieved through specific properties of the Ts radical [60,61]. The high regioselectivity can be explained by the reason that the rate of sulfonyl radical addition to the carbo–carbon double bond of acrylamide is much greater than to the enamine carbon–carbon double bond. Third, radical species II loses an electron by the oxidation of photocatalyst [*fac*-Ir(ppy)_3_]^+^ to forge tertiary cation intermediate III and to regenerate photocatalyst [*fac*-Ir(ppy)_3_] for the next turnover. Last, deprotonation of cation intermediate III occurs in the presence of K_3_PO_4_, giving sulfonylated pyrrolinone **3a**. However, since the presence of base is important for the reaction, it cannot be ruled out that the radical II is directly deprotonated by the base to form radical anion, which is oxidized by the photocatalyst [*fac*-Ir (ppy)_3_]^+^ [62]. It is notable that arylsulfonyl radicals are prone to loss of SO_2_ to form aryl radicals, which could induce the cyclization of 1,5-dienes in the same way as arylsulfonyl radicals, but the corresponding products have not been found in this system [63,64,65,66,67,68].

## 3. Materials and Methods

### 3.1. General Considerations

All the reagents purchased from Leyan company were directly used. ^1^H-NMR and ^13^C-NMR spectra of the products were recorded on a Bruker FT-NMR 400M or 600M spectrometer (Bruker Beijing Scientific Technology Co., Ltd., Beijing, China). Chemical shifts spectra are given as δ in the units of parts per million (ppm) with reference to tetramethylsilane (TMS). Multiplicities were indicated as follows: d (doublet); s (singlet); t (triplet); q (quartet); m (multiplets); etc. Coupling constants are reported as a *J* value in Hz. High-resolution mass spectral analysis (HRMS) of the products were collected on an Agilent Technologies 6540 UHD Accurate-Mass Q-TOF LC/MS (ESI) instrument (Beijing Agilent Technologies Co., Ltd, Beijing, China).

### 3.2. Typical Procedure for the Preparation of ***3a***

1,5-dienes **1a** (0.1 mmol), sulfonyl chlorides **2a** (0.2 mmol), *fac*-Ir(ppy)_3_ (1 mol%), K_3_PO_4_ (1.5 equiv.), and CH_2_Cl_2_ (1 mL) were added into a dry 25 mL Schlenk tube containing a magnetic stirring bar under nitrogen atmosphere, Then the mixture was stirred and irradiated with 5 W white LEDs at room temperature for 16 h. After completing, the reaction mixture was directly subjected to flash column chromatography (10–40% EtOAc/Petroleum ether) to obtain the desired product **3a** as a white solid (79% yield).

### 3.3. Procedure for the Synthesis of the Coupling Product ***4aa***

*n*-BuLi (2.5 M, 0.24 mmol) was slowly added to the solution of compound **4a** (0.2 mmol) and THF (8 mL) at −78 °C. After 15 min, the reaction increased to room temperature. After completing, 8 mL water was added to quench the reaction and the mixture was extracted with 10 mL dichloromethane 3 times. The combined dichloromethane phases were dried over CaCl_2_, concentrated *in vacuo* and purified by flash column chromatography (30–40% EtOAc/petroleum ether) to furnish the desired product **4aa** as a white solid (84% yield).

*1-Acetyl-3-methyl-5-phenyl-3-(tosylmethyl)-1H-pyrrol-2(3H)-one* (**3a**): ^1^H NMR (600 MHz, CDCl_3_) δ 7.71 (d, *J* = 8.3 Hz, 2H), 7.37–7.34 (m, 3H), 7.27 (d, *J* = 8.1 Hz, 2H), 7.24 (dd, *J* = 6.6, 3.0 Hz, 2H), 5.51 (s, 1H), 3.69 (d, *J* = 14.4 Hz, 1H), 3.46 (d, *J* = 14.4 Hz, 1H), 2.49 (s, 3H), 2.42 (s, 3H), 1.39 (s, 3H). δ ^13^C NMR (151 MHz, CDCl_3_) δ 179.60, 169.23, 145.06, 142.87, 136.57, 129.89, 128.50, 128.21, 127.88, 126.78, 115.54, 62.17, 47.76, 26.01, 24.57, 21.61.

*1-Acetyl-5-(4-fluorophenyl)-3-methyl-3-(tosylmethyl)-1H-pyrrol-2(3H)-one* (**3b**): ^1^H NMR (400 MHz, CDCl_3_) δ 7.71 (d, *J* = 8.2 Hz, 2H), 7.29 (d, *J* = 8.1 Hz, 2H), 7.26–7.20 (m, 2H), 7.05 (t, *J* = 8.7 Hz, 2H), 5.53 (s, 1H), 3.69 (d, *J* = 14.3 Hz, 1H), 3.45 (d, *J* = 14.3 Hz, 1H), 2.50 (s, 3H), 2.43 (s, 3H), 1.39 (s, 3H). ^13^C NMR (101 MHz, CDCl_3_) δ 179.56, 169.33, 162.72 (*J* = 252 Hz), 145.15, 142.02, 136.61, 129.94, 128.85, 128.77, 128.14, 115.66, 115.06, 114.85, 62.22, 47.69, 26.05, 24.53, 21.63. ^19^F NMR (565 MHz, CDCl_3_) δ-112.68.

*1-Acetyl-5-(4-chlorophenyl)-3-methyl-3-(tosylmethyl)-1H-pyrrol-2(3H)-one* (**3c**): ^1^H NMR (600 MHz, CDCl_3_) δ 7.70 (d, *J* = 8.3 Hz, 2H), 7.33 (d, *J* = 8.5 Hz, 2H), 7.29 (d, *J* = 8.0 Hz, 2H), 7.19 (d, *J* = 8.5 Hz, 2H), 5.56 (s, 1H), 3.68 (d, *J* = 14.3 Hz, 1H), 3.45 (d, *J* = 14.3 Hz, 1H), 2.50 (s, 3H), 2.43 (s, 3H), 1.39 (s, 3H). ^13^C NMR (151 MHz, CDCl_3_) δ 179.46, 169.26, 145.16, 141.90, 136.55, 134.39, 131.26, 129.94, 128.23, 128.13, 128.13, 116.05, 62.19, 47.74, 25.97, 24.48, 21.62.

*1-acetyl-5-(4-bromophenyl)-3-methyl-3-(tosylmethyl)-1H-pyrrol-2(3H)-one* (**3d**). ^1^H NMR (600 MHz, CDCl_3_) δ 7.70 (d, *J* = 8.2 Hz, 2H), 7.49 (d, *J* = 8.4 Hz, 2H), 7.29 (d, *J* = 8.0 Hz, 2H), 7.13 (d, *J* = 8.4 Hz, 2H), 5.56 (s, 1H), 3.68 (d, *J* = 14.3 Hz, 1H), 3.44 (d, *J* = 14.3 Hz, 1H), 2.50 (s, 3H), 2.43 (s, 3H), 1.39 (s, 3H). ^13^C NMR (101 MHz, CDCl_3_) δ 179.45, 169.27, 145.19, 141.95, 136.53, 131.74, 131.08, 129.96, 128.49, 128.14, 122.60, 116.10, 62.18, 47.77, 25.99, 24.47, 21.65.

*1-Acetyl-5-(4-iodophenyl)-3-methyl-3-(tosylmethyl)-1H-pyrrol-2(3H)-one* (**3e**). ^1^H NMR (400 MHz, CDCl_3_) δ 7.69 (dd, *J* = 8.0, 2.4 Hz, 4H), 7.29 (d, *J* = 7.9 Hz, 2H), 6.99 (d, *J* = 7.6 Hz, 2H), 5.56 (s, 1H), 3.69 (d, *J* = 14.2 Hz, 1H), 3.45 (d, *J* = 14.3 Hz, 1H), 2.50 (s, 3H), 2.43 (s, 3H), 1.38 (s, 3H). ^13^C NMR (101 MHz, CDCl_3_) δ 179.41, 169.24, 145.16, 141.99, 136.97, 136.44, 132.28, 129.93, 128.55, 128.11, 116.10, 94.28, 62.12, 47.75, 25.95, 24.44, 21.63.

*Methyl-4-(1-acetyl-4-methyl-5-oxo-4-(tosylmethyl)-4,5-dihydro-1H-pyrrol-2-yl)benzoate* (**3f**). ^1^H NMR (600 MHz, CDCl_3_) δ 8.03 (d, *J* = 8.3 Hz, 2H), 7.70 (d, *J* = 8.2 Hz, 2H), 5.61 (s, 1H), 3.93 (s, 3H), 3.70 (d, *J* = 14.4 Hz, 1H), 3.48 (d, *J* = 14.4 Hz, 1H), 2.52 (s, 3H), 2.42 (s, 3H), 1.40 (s, 3H). ^13^C NMR (151 MHz, CDCl_3_) δ 179.34, 169.17, 166.61, 145.19, 142.05, 137.21, 136.50, 129.95, 129.20, 128.14, 126.77, 116.93, 62.17, 52.20, 47.90, 25.85, 24.43, 21.62.

*1-Acetyl-3-methyl-5-p-tolyl-3-(tosylmethyl)-1H-pyrrol-2(3H)-one* (**3g**). ^1^H NMR (400 MHz, CDCl_3_) δ 7.71 (d, *J* = 8.3 Hz, 2H), 7.27 (d, *J* = 8.1 Hz, 2H), 7.17 (d, *J* = 8.1 Hz, 2H), 7.13 (d, *J* = 8.3 Hz, 2H), 5.49 (s, 1H), 3.69 (d, *J* = 14.4 Hz, 1H), 3.45 (d, *J* = 14.4 Hz, 1H), 2.47 (s, 3H), 2.42 (s, 3H), 2.38 (s, 3H), 1.39 (s, 3H). ^13^C NMR (101 MHz, CDCl_3_) δ 179.68, 169.28, 145.03, 142.88, 138.45, 136.57, 129.90, 129.76, 128.62, 128.26, 126.71, 114.96, 62.18, 47.72, 26.09, 24.61, 21.62, 21.37.

*1-Acetyl-5-(4-methoxyphenyl)-3-methyl-3-(tosylmethyl)-1H-pyrrol-2(3H)-one* (**3h**). ^1^H NMR (400 MHz, CDCl_3_) δ 7.71 (d, *J* = 8.3 Hz, 2H), 7.20–7.10 (m, 2H), 6.89 (d, *J* = 8.8 Hz, 2H), 5.45 (s, 1H), 3.84 (s, 3H), 3.69 (d, *J* = 14.3 Hz, 1H), 3.45 (d, *J* = 14.4 Hz, 1H), 2.48 (s, 3H), 2.42 (s, 3H), 1.38 (s, 3H). ^13^C NMR (101 MHz, CDCl_3_) δ 179.75, 169.41, 159.75, 145.04, 142.62, 136.59, 129.89, 128.25, 128.23, 125.05, 114.47, 113.35, 62.21, 55.32, 47.64, 26.17, 24.65, 21.63.

*1-Acetyl-5-(3-bromophenyl)-3-methyl-3-(tosylmethyl)-1H-pyrrol-2(3H)-one* (**3i**). ^1^H NMR (600 MHz, CDCl_3_) δ 7.70 (d, *J* = 8.0 Hz, 2H), 7.47 (d, *J* = 7.7 Hz, 1H), 7.35 (s, 1H), 7.31 (d, *J* = 8.0 Hz, 2H), 7.22 (t, *J* = 7.9 Hz, 1H), 7.16 (d, *J* = 7.7 Hz, 1H), 5.51 (s, 1H), 3.69 (d, *J* = 14.4 Hz, 1H), 3.47 (d, *J* = 14.4 Hz, 1H), 2.52 (s, 3H), 2.45 (s, 3H), 1.39 (s, 3H). ^13^C NMR (151 MHz, CDCl_3_) δ 179.33, 169.17, 145.20, 141.52, 136.52, 134.71, 131.45, 129.99, 129.66, 129.33, 128.17, 125.56, 121.91, 116.51, 62.15, 47.79, 25.89, 24.43, 21.66.

*1-Acetyl-3-methyl-5-m-tolyl-3-(tosylmethyl)-1H-pyrrol-2(3H)-one* (**3j**). ^1^H NMR (400 MHz, CDCl_3_) δ 7.74–7.66 (m, 2H), 7.28 (d, *J* = 7.9 Hz, 2H), 7.24 (d, *J* = 7.6 Hz, 1H), 7.16 (d, *J* = 7.6 Hz, 1H), 7.05 (s, 1H), 7.02 (d, *J* = 7.6 Hz, 1H), 5.49 (s, 1H), 3.69 (d, *J* = 14.3 Hz, 1H), 3.46 (d, *J* = 14.4 Hz, 1H), 2.49 (s, 3H), 2.43 (s, 3H), 2.37 (s, 3H), 1.39 (s, 3H). ^13^C NMR (101 MHz, CDCl_3_) δ 179.64, 169.22, 145.03, 142.97, 137.58, 136.58, 132.58, 129.92, 129.34, 128.26, 127.76, 127.32, 123.89, 115.35, 62.17, 47.76, 26.05, 24.58, 21.63, 21.46.

*1-Acetyl-5-(2-chlorophenyl)-3-methyl-3-(tosylmethyl)-1H-pyrrol-2(3H)-one* (**3k**). ^1^H NMR (600 MHz, CDCl_3_) δ 7.78 (d, *J* = 8.0 Hz, 2H), 7.43 (s, 1H), 7.34 (dt, *J* = 14.4, 4.1 Hz, 5H), 5.68 (s, 1H), 3.66 (s, 1H), 3.44 (d, *J* = 14.2 Hz, 1H), 2.44 (d, *J* = 9.1 Hz, 6H), 1.45 (s, 3H). ^13^C NMR (151 MHz, CDCl_3_) δ 178.67, 168.78, 145.16, 132.80, 129.98, 129.92, 129.82, 128.88, 128.11, 126.70, 116.75, 61.92, 47.61, 25.34, 24.43, 21.61.

*1-Acetyl-3-methyl-5-o-tolyl-3-(tosylmethyl)-1H-pyrrol-2(3H)-one* (**3l**). ^1^H NMR (600 MHz, CDCl_3_) δ 7.78 (d, *J* = 8.0 Hz, 2H), 7.33 (d, *J* = 8.0 Hz, 2H), 7.28 (d, *J* = 7.4 Hz, 1H), 7.19 (dd, *J* = 15.2, 7.4 Hz, 2H), 5.58 (s, 1H), 3.67 (d, *J* = 14.0 Hz, 1H), 3.42 (d, *J* = 14.0 Hz, 1H), 2.48 (s, 3H), 2.43 (s, 3H), 2.26 (s, 3H), 1.41 (s, 3H). ^13^C NMR (151 MHz, CDCl_3_) δ 179.56, 168.91, 145.09, 136.91, 133.15, 129.98, 129.57, 128.60, 128.41, 128.00, 125.37, 115.27, 62.16, 47.39, 25.73, 24.93, 21.63, 19.80.

*1-Acetyl-4-ethyl-3-methyl-5-phenyl-3-(tosylmethyl)-1H-pyrrol-2(3H)-one* (**3m**). ^1^H NMR (600 MHz, CDCl_3_) δ 7.76 (d, *J* = 7.9 Hz, 2H), 7.39 (t, *J* = 7.3 Hz, 2H), 7.36 (d, *J* = 7.2 Hz, 1H), 7.34–7.27 (m, 4H), 3.70 (d, *J* = 14.3 Hz, 1H), 3.49 (d, *J* = 14.3 Hz, 1H), 2.44 (s, 3H), 2.43 (s, 3H), 2.21 (dd, *J* = 15.2, 7.7 Hz, 1H), 1.94 (dd, *J* = 15.0, 7.5 Hz, 1H), 1.38 (s, 3H), 0.91 (t, *J* = 7.6 Hz, 3H). ^13^C NMR (151 MHz, CDCl_3_) δ 179.42, 169.08, 145.02, 137.67, 136.88, 132.56, 129.92, 128.32, 128.13, 128.00, 127.93, 126.10, 61.75, 50.46, 26.18, 24.64, 21.63, 18.04, 14.58.

*1-Acetyl-3-butyl-5-phenyl-3-(tosylmethyl)-1H-pyrrol-2(3H)-one* (**3n**). ^1^H NMR (600 MHz, CDCl_3_) δ 7.70 (d, *J* = 8.1 Hz, 2H), 7.38–7.34 (m, 4H), 7.26 (d, *J* = 5.4 Hz, 5H), 5.42 (s, 1H), 3.69 (d, *J* = 14.4 Hz, 1H), 3.49 (d, *J* = 14.4 Hz, 1H), 2.49 (s, 3H), 2.41 (s, 3H), 1.75–1.67 (m, 2H), 1.27 (s, 3H), 1.11 (d, *J* = 11.6 Hz, 1H), 0.85 (t, *J* = 7.0 Hz, 3H). ^13^C NMR (151 MHz, CDCl_3_) δ 179.40, 169.09, 145.00, 143.77, 136.68, 132.82, 129.88, 128.48, 128.22, 127.90, 126.82, 114.00, 61.79, 51.71, 38.07, 26.05, 25.64, 22.60, 21.61, 13.75.

*3-Methyl-5-phenyl-1-propionyl-3-(tosylmethyl)-1H-pyrrol-2(3H)-one* (**3o**). ^1^H NMR (600 MHz, CDCl_3_) δ 7.70 (d, *J* = 8.2 Hz, 2H), 7.38–7.33 (m, 3H), 7.27 (d, *J* = 5.6 Hz, 2H), 7.23 (dd, *J* = 6.5, 2.9 Hz, 2H), 5.51 (s, 1H), 3.70 (d, *J* = 14.4 Hz, 1H), 3.45 (d, *J* = 14.4 Hz, 1H), 2.95–2.81 (m, 2H), 2.41 (s, 3H), 1.39 (s, 3H), 1.14 (t, *J* = 7.3 Hz, 3H). ^13^C NMR (151 MHz, CDCl_3_) δ 179.43, 173.23, 145.03, 143.01, 136.60, 132.85, 129.87, 128.48, 128.22, 127.93, 126.70, 115.49, 62.16, 47.83, 31.58, 24.63, 21.62, 8.33.

*1-Isobutyryl-3-methyl-5-phenyl-3-(tosylmethyl)-1H-pyrrol-2(3H)-one* (**3p**). ^1^H NMR (600 MHz, CDCl_3_) δ ^1^H NMR (600 MHz, CDCl_3_) δ 7.69 (d, *J* = 8.3 Hz, 2H), 7.36 (dd, *J* = 5.0, 1.8 Hz, 3H), 7.24 (d, *J* = 8.0 Hz, 2H), 7.19 (dd, *J* = 6.5, 3.1 Hz, 2H), 5.49 (s, 1H), 3.70 (d, *J* = 14.4 Hz, 1H), 3.66 (s, 1H), 3.46 (d, *J* = 14.4 Hz, 1H), 2.40 (s, 3H), 1.40 (s, 3H), 1.24 (d, *J* = 6.9 Hz, 3H), 1.18 (d, *J* = 6.8 Hz, 3H). ^13^C NMR (151 MHz, CDCl_3_) δ 178.91, 176.67, 144.97, 143.15, 136.68, 132.81, 129.86, 128.49, 128.18, 128.05, 126.22, 115.31, 62.11, 48.11, 35.57, 24.69, 21.62, 18.60, 18.37.

*1-Acetyl-3-methyl-5-phenyl-3-(phenylsulfonylmethyl)-1H-pyrrol-2(3H)-one* (**4a**). ^1^H NMR (600 MHz, CDCl_3_) δ 7.84 (d, *J* = 7.6 Hz, 2H), 7.63 (t, *J* = 7.4 Hz, 1H), 7.49 (t, *J* = 7.8 Hz, 2H), 7.37–7.33 (m, 3H), 7.24 (dd, *J* = 6.4, 2.6 Hz, 2H), 5.50 (s, 1H), 3.71 (d, *J* = 14.4 Hz, 1H), 3.49 (d, *J* = 14.4 Hz, 1H), 2.51 (s, 3H), 1.41 (s, 3H). ^13^C NMR (151 MHz, CDCl_3_) δ 179.58, 169.29, 143.05, 139.60, 133.94, 132.63, 129.30, 128.54, 128.17, 127.92, 126.76, 115.37, 62.13, 47.78, 26.07, 24.53.

*1-Acetyl-3-((4-methoxyphenylsulfonyl)methyl)-3-methyl-5-phenyl-1H-pyrrol-2(3H)-one* (**4b**). ^1^H NMR (600 MHz, CDCl_3_) δ 7.74 (d, *J* = 8.8 Hz, 2H), 7.42–7.32 (m, 3H), 6.91 (d, *J* = 8.9 Hz, 2H), 5.50 (s, 1H), 3.84 (s, 3H), 3.70 (d, *J* = 14.4 Hz, 1H), 3.45 (d, *J* = 14.4 Hz, 1H), 2.50 (s, 3H), 1.39 (s, 3H).^13^C NMR (151 MHz, CDCl_3_) δ 179.60, 169.30, 163.90, 142.80, 132.71, 130.90, 130.46, 128.50, 127.90, 126.77, 115.66, 114.45, 62.36, 55.73, 47.80, 26.04, 24.64.

*1-Acetyl-3-((4-tert-butylphenylsulfonyl)methyl)-3-methyl-5-phenyl-1H-pyrrol-2(3H)-one* (**4c**). ^1^H NMR (600 MHz, CDCl_3_) δ 7.73 (d, *J* = 8.5 Hz, 2H), 7.47 (d, *J* = 8.5 Hz, 2H), 7.38–7.32 (m, 3H), 7.21 (dd, *J* = 3.9, 1.8 Hz, 2H), 5.45 (s, 1H), 3.71 (d, *J* = 14.4 Hz, 1H), 3.48 (d, *J* = 14.4 Hz, 1H), 2.50 (s, 3H), 1.40 (s, 3H), 1.32 (s, 9H). ^13^C NMR (151 MHz, CDCl_3_) δ 179.57, 169.24, 157.94, 142.81, 136.43, 132.64, 128.49, 128.04, 127.88, 126.73, 126.32, 115.56, 62.07, 47.75, 35.27, 31.02, 26.10, 24.55.

*1-Acetyl-3-methyl-5-phenyl-3-((m-tolylsulfonyl)methyl)-1,3-dihydro-2H-pyrrol-2-one* (**4d**). White solid; mp 136.3–138.0 °C; ^1^H NMR (600 MHz, CDCl_3_) δ 7.62 (d, *J* = 11.2 Hz, 2H), 7.41 (d, *J* = 7.6 Hz, 1H), 7.39–7.33 (m, 4H), 7.24 (dd, *J* = 6.6, 2.9 Hz, 2H), 5.46 (s, 1H), 3.71 (d, *J* = 14.4 Hz, 1H), 3.48 (d, *J* = 14.4 Hz, 1H), 2.53 (s, 3H), 2.32 (s, 3H), 1.40 (s, 3H). ^13^C NMR (151 MHz, CDCl_3_) δ 179.65, 169.31, 143.07, 139.70, 139.47, 134.73, 132.68, 129.17, 128.54, 128.48, 127.93, 126.77, 125.22, 115.33, 62.13, 47.79, 26.08, 24.51, 21.19. HRMS (ESI, *m*/*z*): Calcd. For C_21_H_21_NSO_4_Na [M + Na]^+^ 406.1083, found: 406.1085.

*1-Acetyl-3-(((2-methoxyphenyl)sulfonyl)methyl)-3-methyl-5-phenyl-1,3-dihydro-2H-pyrrol-2-one* (**4e**). White solid; mp 123.4–125.0 °C; ^1^H NMR (600 MHz, CDCl_3_) δ 7.76 (dd, *J* = 7.8, 1.7 Hz, 1H), 7.59–7.53 (m, 1H), 7.34–7.29 (m, 3H), 7.12 (dd, *J* = 6.5, 3.1 Hz, 2H), 7.03 (d, *J* = 8.3 Hz, 1H), 6.95 (t, *J* = 7.6 Hz, 1H), 5.37 (s, 1H), 4.02 (s, 3H), 3.95 (d, *J* = 14.5 Hz, 1H), 3.77 (d, *J* = 14.6 Hz, 1H), 2.47 (s, 3H), 1.39 (s, 3H). ^13^C NMR (151 MHz, CDCl_3_) δ 179.69, 169.24, 157.38, 142.70, 135.86, 132.68, 130.67, 128.39, 127.79, 127.19, 126.65, 120.87, 115.64, 112.35, 60.13, 56.47, 47.69, 26.00, 24.55. HRMS (ESI, *m*/*z*): Calcd. For C_21_H_21_NO_5_SNa [M + Na]^+^ 422.1033, found: 422.1038.

*1-Acetyl-3-((4-bromophenylsulfonyl)methyl)-3-methyl-5-phenyl-1H-pyrrol-2(3H)-one* (**4f**). ^1^H NMR (400 MHz, CDCl_3_) δ 7.67 (d, *J* = 8.0 Hz, 2H), 7.61 (d, *J* = 7.9 Hz, 2H), 7.36 (s, 3H), 7.22 (s, 2H), 5.48 (s, 1H), 3.70 (d, *J* = 14.4 Hz, 1H), 3.46 (d, *J* = 14.3 Hz, 1H), 2.52 (s, 3H), 1.39 (s, 3H). ^13^C NMR (101 MHz, CDCl_3_) δ 179.45, 169.28, 143.22, 138.49, 132.66, 132.50, 129.75, 129.44, 128.67, 128.01, 126.71, 115.17, 62.20, 47.77, 26.03, 24.57. HRMS (ESI, *m*/*z*): Calcd. For C_20_H_18_NO_4_SBrNa [M + Na]^+^ 470.0032, found: 470.0035.

*1-Acetyl-3-((4-iodophenylsulfonyl)methyl)-3-methyl-5-phenyl-1H-pyrrol-2(3H)-one* (**4g**). ^1^H NMR (600 MHz, CDCl_3_) δ 7.84 (d, *J* = 8.3 Hz, 2H), 7.52 (d, *J* = 8.4 Hz, 2H), 7.37 (d, *J* = 1.6 Hz, 3H), 7.22 (d, *J* = 3.6 Hz, 2H), 5.49 (s, 1H), 3.70 (d, *J* = 14.4 Hz, 1H), 3.46 (d, *J* = 14.4 Hz, 1H), 2.52 (s, 3H), 1.40 (s, 3H). ^13^C NMR (151 MHz, CDCl_3_) δ 179.41, 169.25, 143.17, 139.09, 138.63, 132.47, 129.49, 128.65, 127.99, 126.69, 115.16, 102.04, 62.12, 47.73, 26.03, 24.58.

*4-(((1-Acetyl-3-methyl-2-oxo-5-phenyl-2,3-dihydro-1H-pyrrol-3-yl)methyl)sulfonyl)benzonitrile* (**4h**). White solid; mp 189.4–191.5 °C; ^1^H NMR (600 MHz, CDCl_3_) δ 7.94 (d, *J* = 8.1 Hz, 2H), 7.76 (d, *J* = 8.1 Hz, 2H), 7.38 (s, 3H), 7.22 (d, *J* = 3.2 Hz, 2H), 5.43 (s, 1H), 3.72 (d, *J* = 14.4 Hz, 1H), 3.52 (d, *J* = 14.4 Hz, 1H), 2.55 (s, 3H), 1.41 (s, 3H). ^13^C NMR (151 MHz, CDCl_3_) δ 179.31, 169.26, 143.63, 143.57, 133.03, 132.35, 128.87, 128.79, 128.06, 126.61, 117.72, 116.90, 114.77, 62.11, 47.75, 26.05, 24.39. HRMS (ESI, *m*/*z*): Calcd. For C_21_H_18_N_2_O_4_SNa [M + Na]^+^ 417.0879, found: 417.0883.

*1-Acetyl-3-methyl-5-phenyl-3-((4(trifluoromethyl)phenylsulfonyl)methyl)-1H-pyrrol-2(3H)-one* (**4i**). ^1^H NMR (400 MHz, CDCl_3_) δ 7.96 (d, *J* = 7.7 Hz, 2H), 7.74 (d, *J* = 7.7 Hz, 2H), 7.37 (s, 3H), 7.21 (s, 2H), 5.45 (s, 1H), 3.74 (d, *J* = 14.4 Hz, 1H), 3.52 (d, *J* = 14.4 Hz, 1H), 2.52 (s, 3H), 1.41 (s, 3H). ^13^C NMR (101 MHz, CDCl_3_) δ 179.35, 169.30, 143.37, 142.98, 135.63 (*J* = 32 Hz), 132.38, 128.86, 128.74, 128.03, 126.63, 126.48 (*J* = 3 Hz), 125.28 (*J* = 250 Hz), 114.99, 76.75, 62.08, 47.74, 26.03, 24.53. ^19^F NMR (565 MHz, CDCl_3_) δ-63.25.

*1-Acetyl-3-methyl-3-((4-nitrophenylsulfonyl)methyl)-5-phenyl-1H-pyrrol-2(3H)-one* (**4j**). ^1^H NMR (400 MHz, CDCl_3_) δ 8.29 (d, *J* = 8.1 Hz, 2H), 8.01 (d, *J* = 8.3 Hz, 2H), 7.37 (s, 3H), 7.22 (d, *J* = 3.7 Hz, 2H), 5.44 (s, 1H), 3.74 (d, *J* = 14.3 Hz, 1H), 3.54 (d, *J* = 14.4 Hz, 1H), 2.55 (s, 3H), 1.41 (s, 3H). ^13^C NMR (101 MHz, CDCl_3_) δ 179.29, 169.27, 150.85, 145.06, 143.63, 132.28, 129.62, 128.80, 128.06, 126.57, 124.44, 114.68, 62.16, 47.75, 26.05, 24.38.

*1-Acetyl-3-((3-chlorophenylsulfonyl)methyl)-3-methyl-5-phenyl-1H-pyrrol-2(3H)-one* (**4k**). ^1^H NMR (400 MHz, CDCl_3_) δ 7.82 (s, 1H), 7.72 (d, *J* = 7.3 Hz, 1H), 7.60 (d, *J* = 7.8 Hz, 1H), 7.43 (t, *J* = 7.9 Hz, 1H), 7.36 (s, 2H), 7.26 (s, 2H), 5.49 (s, 1H), 3.72 (d, *J* = 14.4 Hz, 1H), 3.50 (d, *J* = 14.4 Hz, 1H), 2.55 (s, 3H), 1.41 (s, 3H). ^13^C NMR (101 MHz, CDCl_3_) δ 179.45, 169.26, 143.35, 141.29, 135.56, 134.13, 132.44, 130.61, 128.60, 128.13, 127.96, 126.70, 126.27, 114.91, 62.13, 47.75, 26.08, 24.45.

*1-Acetyl-3-((2-chlorophenylsulfonyl)methyl)-3-methyl-5-phenyl-1H-pyrrol-2(3H)-one* (**4l**). ^1^H NMR (400 MHz, CDCl_3_) δ 7.91 (d, *J* = 7.7 Hz, 1H), 7.52 (d, *J* = 6.7 Hz, 2H), 7.33 (s, 3H), 7.26–7.20 (m, 1H), 7.13 (d, *J* = 3.3 Hz, 2H), 5.29 (s, 1H), 3.92 (s, 2H), 2.53 (s, 3H), 1.40 (s, 3H). ^13^C NMR (101 MHz, CDCl_3_) δ 179.38, 169.33, 143.22, 137.02, 134.93, 132.62, 132.48, 131.84, 128.52, 127.86, 127.46, 126.52, 115.06, 60.08, 47.68, 26.05, 24.38.

*1-Acetyl-3-methyl-5-phenyl-3-((thiophen-2ylsulfonyl)methyl)-1H-pyrrol-2(3H)-one* (**4m**). ^1^H NMR (600 MHz, CDCl_3_) δ 7.70 (d, *J* = 4.4 Hz, 1H), 7.62 (d, *J* = 3.0 Hz, 1H), 7.40–7.33 (m, 3H), 7.26 (d, *J* = 4.9 Hz, 2H), 7.11–7.02 (m, 1H), 5.59 (s, 1H), 3.82 (d, *J* = 14.4 Hz, 1H), 3.59 (d, *J* = 14.4 Hz, 1H), 2.53 (s, 3H), 1.43 (s, 3H). ^13^C NMR (151 MHz, CDCl_3_) δ 179.45, 169.26, 143.15, 140.70, 134.67, 134.58, 132.61, 128.55, 127.92, 126.84, 115.19, 63.58, 47.89, 26.09, 24.46.

*1-Acetyl-3-(cyclopropylsulfonylmethyl)-3-methyl-5-phenyl-1H-pyrrol-2(3H)-one* (**4n**). ^1^H NMR (600 MHz, CDCl_3_) δ 7.36–7.33 (m, 3H), 7.28 (dd, *J* = 6.5, 2.9 Hz, 2H), 5.72 (s, 1H), 3.62 (d, *J* = 14.0 Hz, 1H), 3.41 (d, *J* = 14.0 Hz, 1H), 2.57 (s, 3H), 2.41–2.36 (m, 1H), 1.47 (s, 3H), 1.28–1.25 (m, 1H), 1.21 (dd, *J* = 4.8, 1.8 Hz, 1H), 1.05–1.01 (m, 2H). ^13^C NMR (151 MHz, CDCl_3_) δ 179.93, 169.38, 143.37, 132.73, 128.53, 127.93, 126.93, 115.39, 59.87, 47.49, 31.27, 26.11, 24.19, 5.35, 5.14.

*1-Acetyl-3-((ethylsulfonyl)methyl)-3-methyl-5-phenyl-1,3-dihydro-2H-pyrrol-2-one* (**4o**). Amorphous solid; ^1^H NMR (600 MHz, CDCl_3_) δ 7.37–7.32 (m, 3H), 7.28 (dd, *J* = 6.6, 3.0 Hz, 2H), 5.69 (s, 1H), 3.49 (d, *J* = 13.9 Hz, 1H), 3.33 (d, *J* = 13.9 Hz, 1H), 2.98 (d, *J* = 7.5 Hz, 2H), 2.57 (s, 3H), 1.46 (s, 3H), 1.38 (t, *J* = 7.5 Hz, 3H). ^13^C NMR (151 MHz, CDCl_3_) δ 179.90, 169.36, 143.63, 132.75, 128.55, 127.93, 126.97, 115.02, 57.84, 49.54, 47.30, 26.11, 24.15, 6.57. HRMS (ESI, *m*/*z*): Calcd. For C_16_H_19_NSO_4_Na [M + Na]^+^ 344.0927, found: 344.0932.

*3-Methyl-5-phenyl-3-((phenylsulfonyl)methyl)-1,3-dihydro-2H-pyrrol-2-one* (**4aa**). White solid; mp 186.5–188.4 °C; ^1^H NMR (600 MHz, CDCl_3_) δ 8.60 (s, 1H), 7.83 (d, *J* = 7.3 Hz, 2H), 7.60 (t, *J* = 7.5 Hz, 1H), 7.48–7.42 (m, 6H), 7.39 (dd, *J* = 8.2, 5.6 Hz, 1H), 5.75 (d, *J* = 1.8 Hz, 1H), 3.61 (d, *J* = 14.3 Hz, 1H), 3.50 (d, *J* = 14.3 Hz, 1H), 1.44 (s, 3H). ^13^C NMR (151 MHz, CDCl_3_) δ 182.28, 139.99, 139.92, 133.78, 129.49, 129.35, 129.08, 128.94, 128.18, 124.94, 107.88, 77.24, 77.03, 76.82, 61.68, 48.27, 23.52. HRMS (ESI, *m*/*z*): Calcd. For C_18_H_17_NO_3_SNa [M + Na]^+^ 350.0821, found: 350.0827.

*(2-Tosylethene-1,1-diyl)dibenzene.* ^1^H NMR (600 MHz, CDCl_3_) δ 7.47 (d, *J* = 8.1 Hz, 2H), 7.37 (dd, *J* = 14.0, 7.4 Hz, 2H), 7.30 (t, *J* = 7.6 Hz, 4H), 7.20 (d, *J* = 7.6 Hz, 2H), 7.15 (d, *J* = 8.1 Hz, 2H), 7.10 (d, *J* = 7.4 Hz, 2H), 6.99 (s, 1H), 2.38 (s, 3H). ^13^C NMR (151 MHz, CDCl_3_) δ 154.71, 143.76, 139.26, 138.63, 135.59, 130.23, 129.79, 129.34, 128.98, 128.85, 128.65, 128.58, 128.22, 127.82, 127.71, 126.05, 21.58.

## 4. Conclusions

In conclusion, we developed a visible-light-induced, regioselective cascade sulfonylation/cyclization of 1,5-dienes with sulfonyl chlorides. A variety of structurally significant pyrrolinones with important classes of sulfonyl group patterns were obtained in medium to high yields. This methodology features sulfonyl radical addition/cyclization of alkenes C(sp^2^)-H with high regioselectivity under very mild conditions and tolerated broad functional groups.

## Figures and Tables

**Table 1:** Optimization of the reaction conditions ^a^.

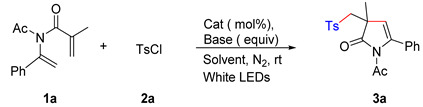				
Entry	Catalyst (1 mol%)	Base (Equiv.)	Solvent	Yield ^b^
1	*fac*-Ir(ppy)_3_	Na_2_CO_3_ (1.0)	CH_2_Cl_2_	57%
2	Ru(bpy)_3_Cl_2_	Na_2_CO_3_ (1.0)	CH_2_Cl_2_	trace
3	Eosin Y	Na_2_CO_3_ (1.0)	CH_2_Cl_2_	trace
4	*fac*-Ir(ppy)_3_	K_2_CO_3_ (1.0)	CH_2_Cl_2_	66%
5	*fac*-Ir(ppy)_3_	Li_2_CO_3_ (1.0)	CH_2_Cl_2_	59%
6	*fac*-Ir(ppy)_3_	NaHCO_3_ (1.0)	CH_2_Cl_2_	64%
7	*fac*-Ir(ppy)_3_	K_3_PO_4_ (1.0)	CH_2_Cl_2_	72%
8	*fac*-Ir(ppy)_3_	Na_3_PO_4_ (1.0)	CH_2_Cl_2_	63%
9	*fac*-Ir(ppy)_3_	K_3_PO_4_ (1.0)	DCE	55%
10	*fac*-Ir(ppy)_3_	K_3_PO_4_ (1.0)	CHCl_3_	62%
11	*fac*-Ir(ppy)_3_	K_3_PO_4_ (1.0)	Acetone	67%
12	*fac*-Ir(ppy)_3_	K_3_PO_4_ (1.0)	Toluene	44%
13	*fac*-Ir(ppy)_3_	K_3_PO_4_ (1.0)	THF	59%
14	*fac*-Ir(ppy)_3_	K_3_PO_4_ (1.0)	EtOAc	32%
15	*fac*-Ir(ppy)_3_	K_3_PO_4_ (1.5)	CH_2_Cl_2_	79%
16	*fac*-Ir(ppy)_3_	K_3_PO_4_ (2.0)	CH_2_Cl_2_	79%
17 ^c^	*fac*-Ir(ppy)_3_	K_3_PO_4_ (1.5)	CH_2_Cl_2_	79%
18 ^c^	------	K_3_PO_4_ (1.5)	CH_2_Cl_2_	0%
19 ^c^	*fac*-Ir(ppy)_3_	------	CH_2_Cl_2_	16%
20 ^d^	*fac*-Ir(ppy)_3_	K_3_PO_4_ (1.5)	CH_2_Cl_2_	0%

^a^ Reaction conditions: **1a** (0.1 mmol), **2a** (0.2 mmol), and catalyst (1 mol%) in solvent (1 mL), which were irradiated with 20 W white LEDs at room temperature under N_2_ for 16 h. ^b^ Isolated yields. ^c^ 5 W white LEDs was used. ^d^ The reaction was conducted in darkness.

## Data Availability

Not applicable.

## Supplementary Materials

The following supporting information can be downloaded at: https://www.mdpi.com/article/10.3390/molecules28145473/s1. Section S1, General information. Section S2, Procedure for the synthesis of compound **3a**–**3p**, **4a**–**4o**. Section S3, Procedures for the formation of compound **4aa.** Section S4, The Transformation with the Light ON/OFF over Time. Section S5, The radical trapping reaction residue. Section S6, NMR spectra for the products.

## Sample Availability

Samples of the compounds are available from the authors.
