# Supplementary material for: Photoredox-Catalyzed Synthesis of 3-Sulfonylated Pyrrolin-2-ones via a Regioselective Tandem Sulfonylation Cyclization of 1,5-Dienes
Source: Molecules. 2023 Jul 17;28(14):5473. doi: 10.3390/molecules28145473 (PMC10386375; doi:10.3390/molecules28145473)
Supplement: Supplementary file 1 [file molecules-28-05473-s001.zip › molecules-2436052-supplementary.pdf]

Supporting Information

Photoredox-Catalyzed Synthesis of 3-Sulfonylated Pyrrolin-2-ones via a Regioselective Tandem Sulfonylation Cyclization of 1,5-Dienes

Ran Ding [1,*], Liang Li [1], Ya-Ting Yu [1], Bing Zhang [1] and Pei-Long Wang [2,3,*]

[1] College of Chemistry and Materials Engineering, Anhui Science and Technology University, Bengbu 233100, China; ll15855863179@126.com (L.L.); yyt2747196669@126.com (Y.-T.Y.); zhangbing2168@126.com (B.Z.)
[2] Key Laboratory of Green and Precise Synthetic Chemistry and Applications, Ministry of Education, School of Chemistry and Materials Science, Huaibei Normal University, Huaibei 235000, China
[3] Information College, Huaibei Normal University, Huaibei 235000, China
* Correspondence: dingran@mail.ustc.edu.cn (R.D.); wangpl@chnu.edu.cn (P.-L.W.)

Table of Contents

S1. General information 1

S2. Procedure for the synthesis of compound **3a – 3p, 4a – 4o** 2

S3. Procedures for the formation of compound **4aa**...4

S4.The Transformation with the Light ON/OFF over Time..4

S5. The radical trapping reaction residue...4

S6. NMR spectra for the products.. 19

S1. General information

All the reagents purchased from Leyan company were directly used. 1H-NMR and 13C-NMR spectra of the products were recorded on a Bruker FT-NMR 400M or 600M spectrometers. Chemical shifts spectra are given as δ in the units of parts per million (ppm) with reference to tetramethylsilane (TMS). Multiplicities were indicated as follows: d (doublet); s (singlet); t (triplet); q (quartet); m (multiplets) and etc. Coupling constants are reported as a J value in Hz. High resolution mass spectral analysis (HRMS) of the products were collected on an Agilent Technologies 6540 UHD Accurate-Mass Q-TOF LC/MS (ESI) instrument.

S2. Procedure for the synthesis of compound 3a – 3p, 4a – 4o.

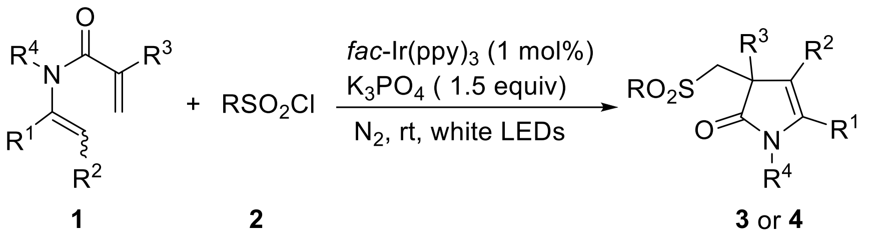

A dry 25-mL Schlenk tube containing a magnetic stirring bar was charged with 1,5-dienes **1** (0.1 mmol), sulfonyl chlorides **2** (0.2 mmol), *fac*-Ir(ppy)$_3$ (1 mol%), K_3PO_4 (1.5 equiv), and CH_2Cl_2 (1 mL), Then the mixture was irradiated with white LEDs at room temperature under N_2 for 16 h. After finishing, the reaction mixture was concentrated on a rotary evaporator and the residue was directly subjected to flash column chromatography on silica gel with (10-40% EtOAc/Petroleum ether) as eluate to furnish the desired product.

S3. Procedures for the formation of 4aa

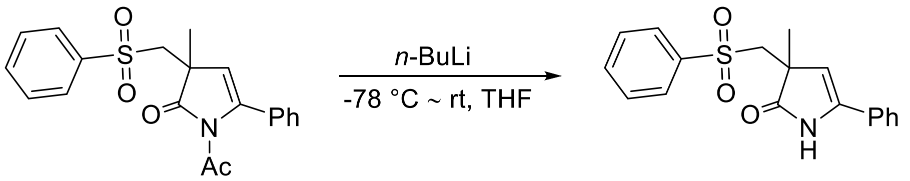

n-BuLi (2.5 M, 0.24 mmol) was slowly added to the solution of compound **4a** (0.2 mmol) and THF (8 mL) at −78 °C. After 15 minutes the reaction has risen to room temperature. After completing, 8 mL water was added to quench the reaction. Then, the mixture was extracted with 10 mL dichloromethane for 3 times. The combined dichloromethane were dried over $CaCl_2$, concentrated in vacuo and purified by flash column chromatography (30% EtOAc/Petroleum ether) to furnish the desired product **4aa** (84% yield).

S4. The Transformation with the Light ON/OFF over Time

The reaction mixture was stirred for 60 min with light-off. All of the following yields were analyzed in the identical way after a 60 min light on or off.

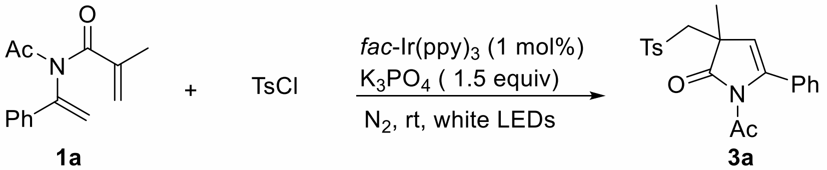

Time (h)	0	1	2	3	4	5	6
On/off	off	on	off	on	off	on	off
Yield (%)	0	26	26	46	46	62	62

S5. The radical trapping reaction residue was analyzed by high resolution mass spectrometry (HRMS)

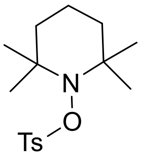

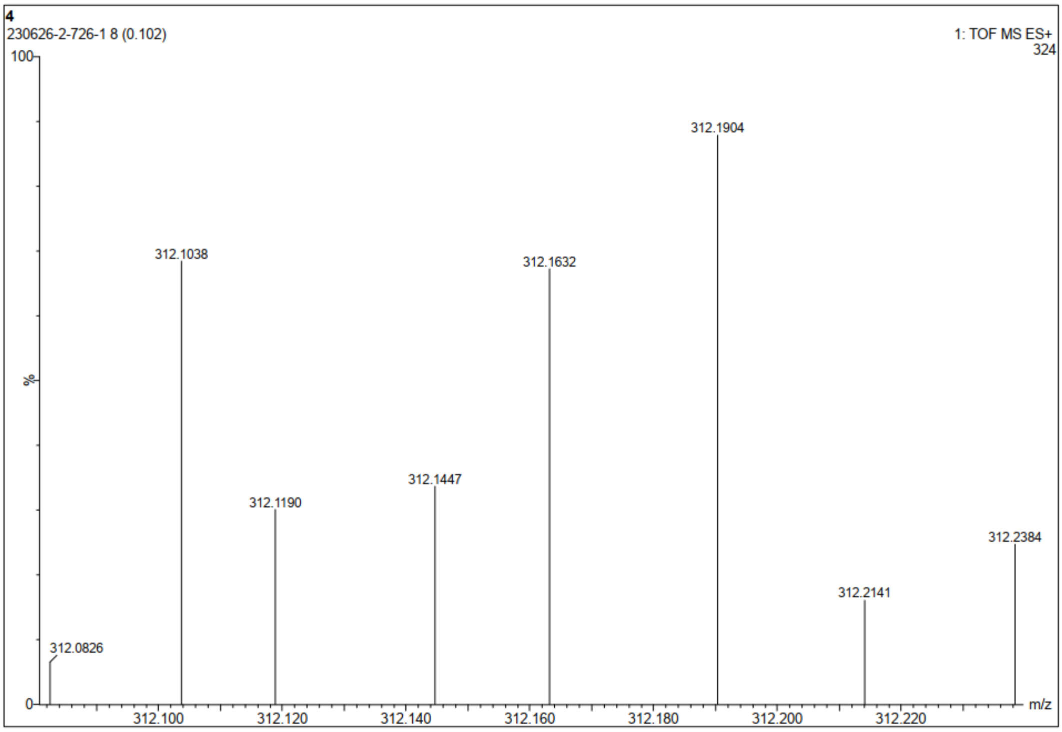

Single Mass Analysis
Tolerance = 5.0 mDa / DBE: min = -1.5, max = 50.0
Element prediction: Off
Number of isotope peaks used for i-FIT = 3

Monoisotopic Mass, Even Electron Ions
459 formula(e) evaluated with 1 results within limits (all results (up to 1000) for each mass)
Elements Used:
C: 16-16 H: 4-50 N: 0-200 O: 0-200 S: 1-3

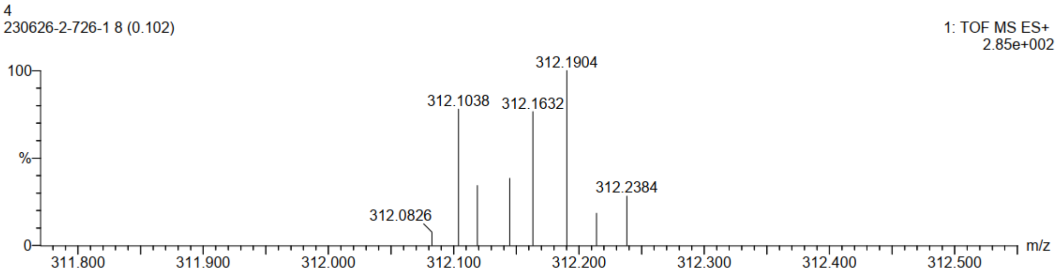

Minimum:				-1.5				
Maximum:		5.0	10.0	50.0				
Mass	Calc. Mass	mDa	PPM	DBE	i-FIT	Norm	Conf(%)	Formula
312.1632	312.1633	-0.1	-0.3	4.5	47.5	n/a	n/a	C16 H26 N O3 S

S6. NMR spectra

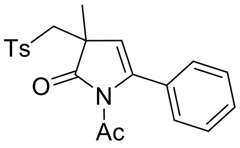

3a

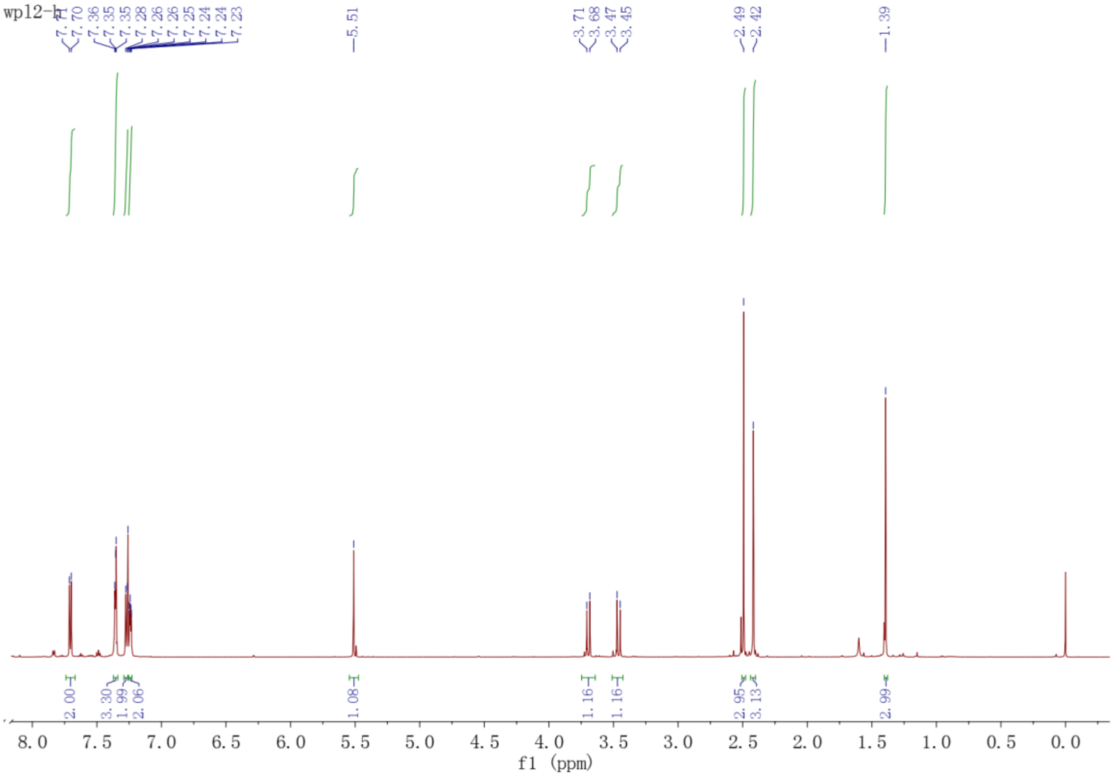

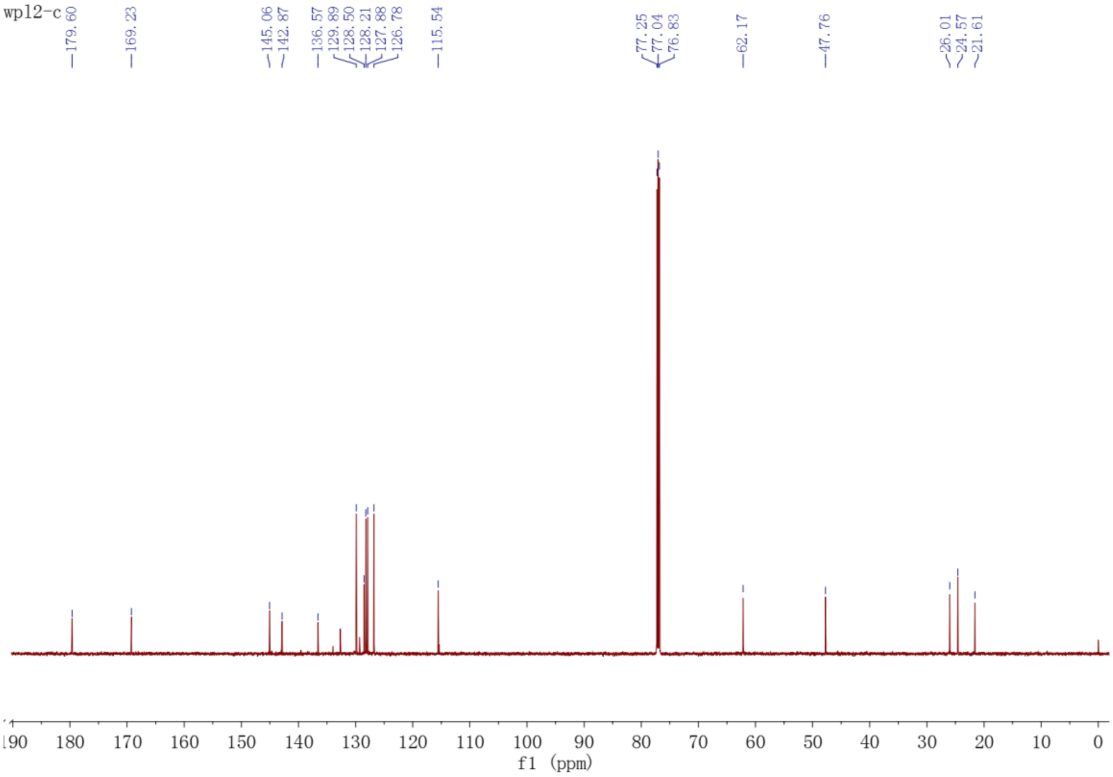

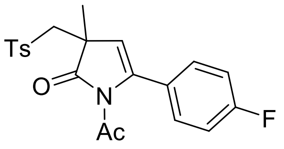
Ts
O
N
Ac
F

3b

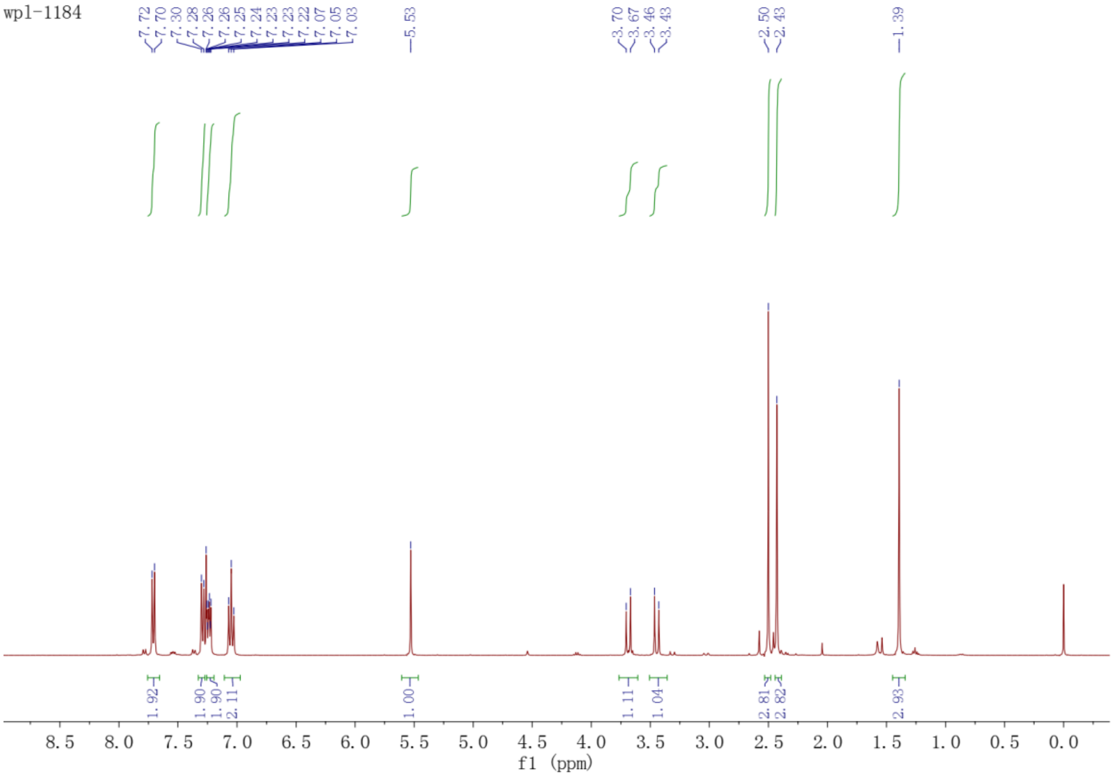
wpl-1184
7.72
7.70
7.30
7.28
7.26
7.26
7.25
7.24
7.23
7.23
7.22
7.07
7.05
7.03
5.53
3.70
3.67
3.46
3.43
2.50
2.43
1.39
1.92
1.90
1.90
2.11
1.00
1.11
1.04
2.81
2.82
2.93
8.5
8.0
7.5
7.0
6.5
6.0
5.5
5.0
4.5
4.0
3.5
3.0
2.5
2.0
1.5
1.0
0.5
0.0
f1 (ppm)

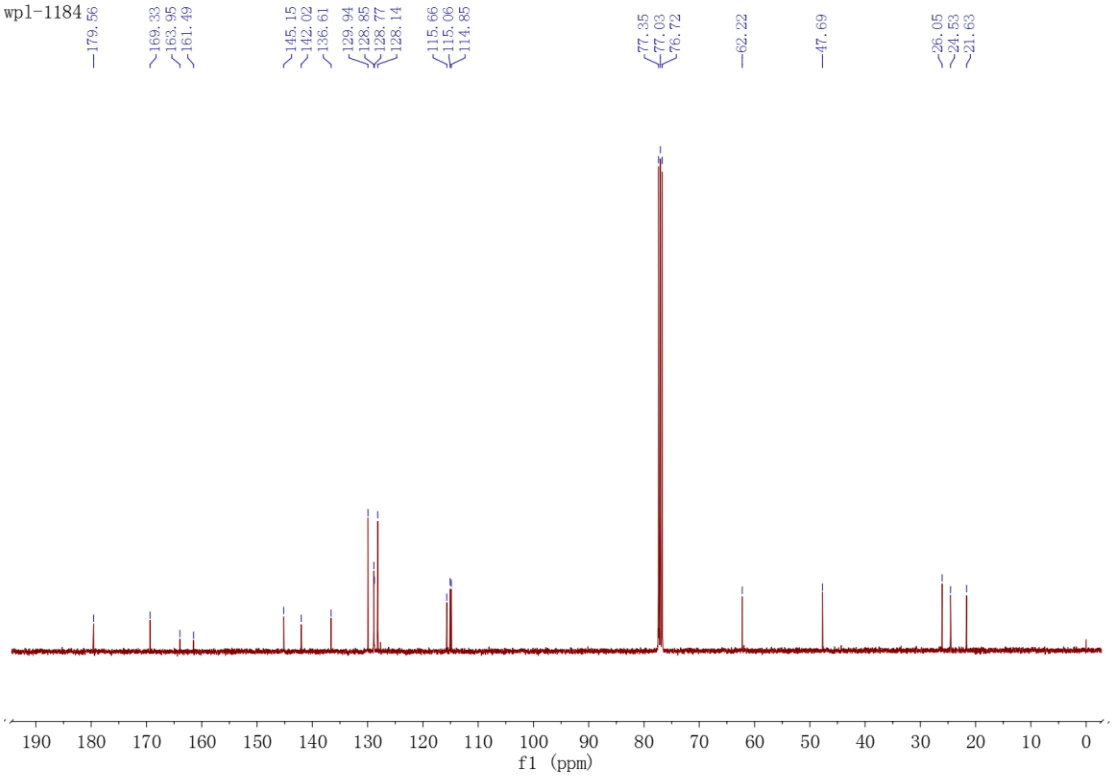
wpl-1184
179.56
169.33
163.95
161.49
145.15
142.02
136.61
129.94
128.85
128.77
128.14
115.66
115.06
114.85
77.35
77.03
76.72
62.22
47.69
26.05
24.53
21.63
190
180
170
160
150
140
130
120
110
100
90
80
70
60
50
40
30
20
10
0
f1 (ppm)

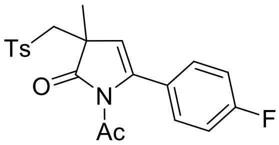
Ts
O
N
Ac
F

3b

wpl-1184

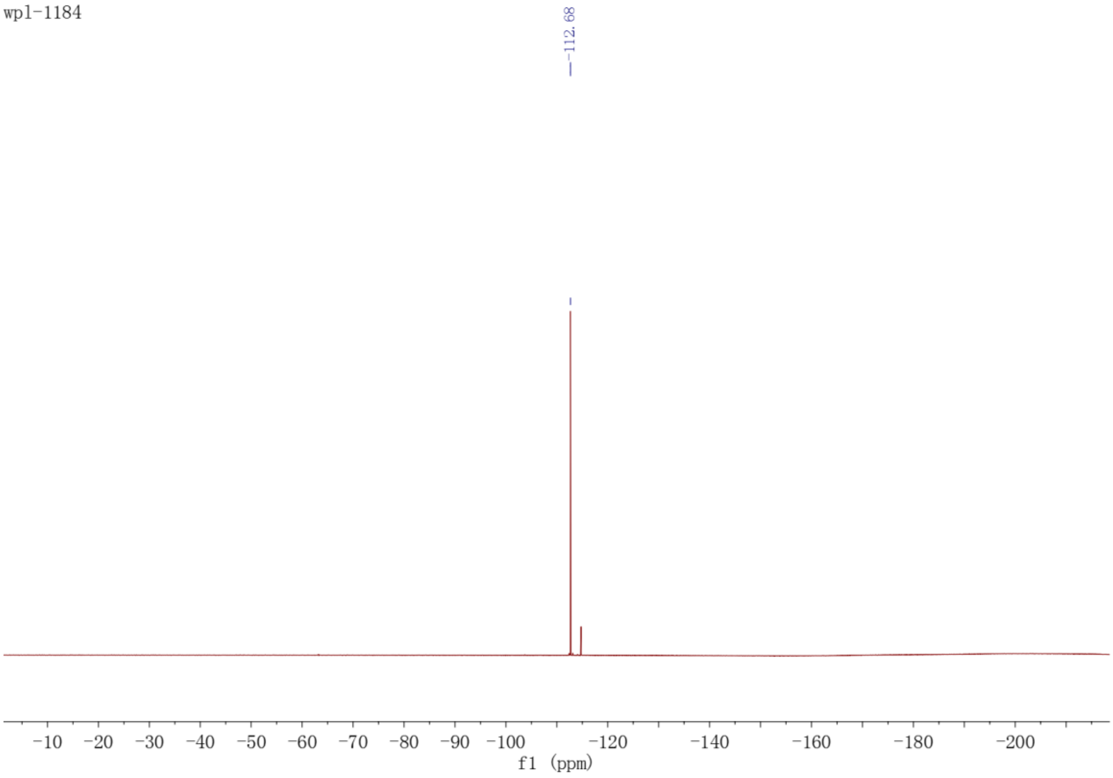
−112.68
−10 −20 −30 −40 −50 −60 −70 −80 −90 −100 −120 −140 −160 −180 −200
f1 (ppm)

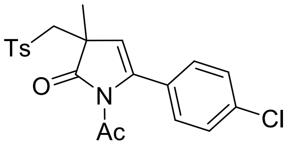
Ts
O
N
Ac
Cl

3c

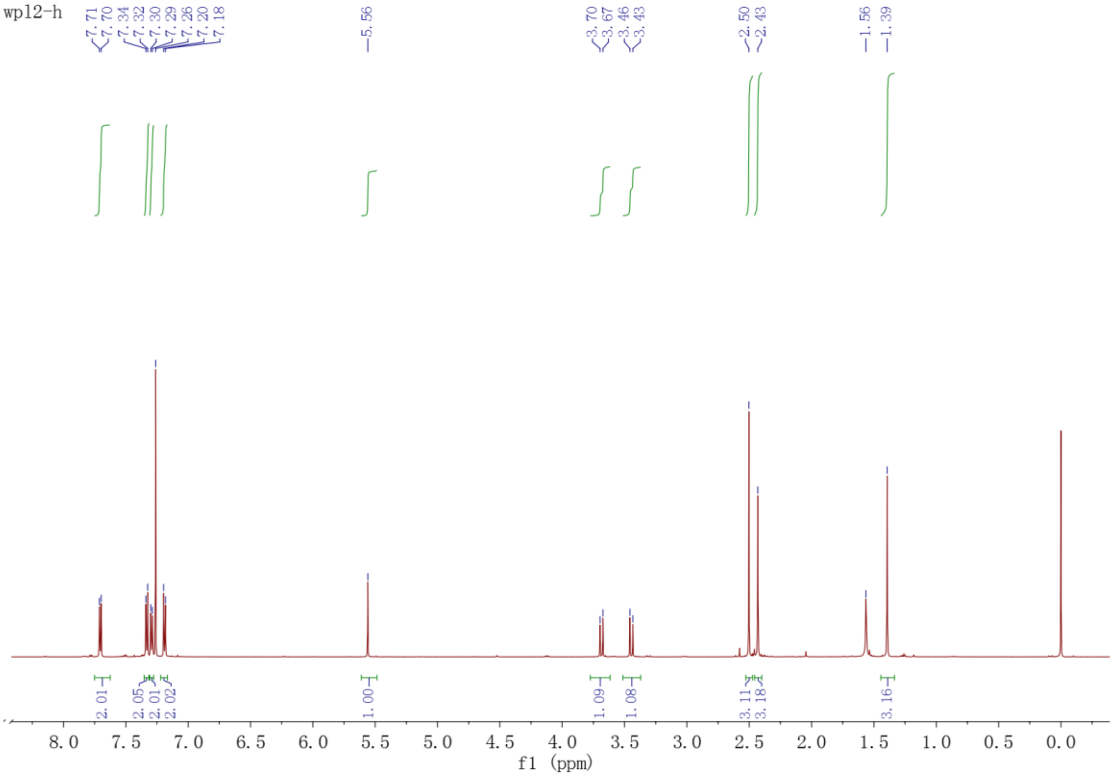
wp12-h
7.71
7.70
7.34
7.32
7.30
7.29
7.26
7.20
7.18
5.56
3.70
3.67
3.46
3.43
2.50
2.43
1.56
1.39
2.01
2.05
2.01
2.02
1.00
1.09
1.08
3.11
3.18
3.16
8.0 7.5 7.0 6.5 6.0 5.5 5.0 4.5 4.0 3.5 3.0 2.5 2.0 1.5 1.0 0.5 0.0
f1 (ppm)

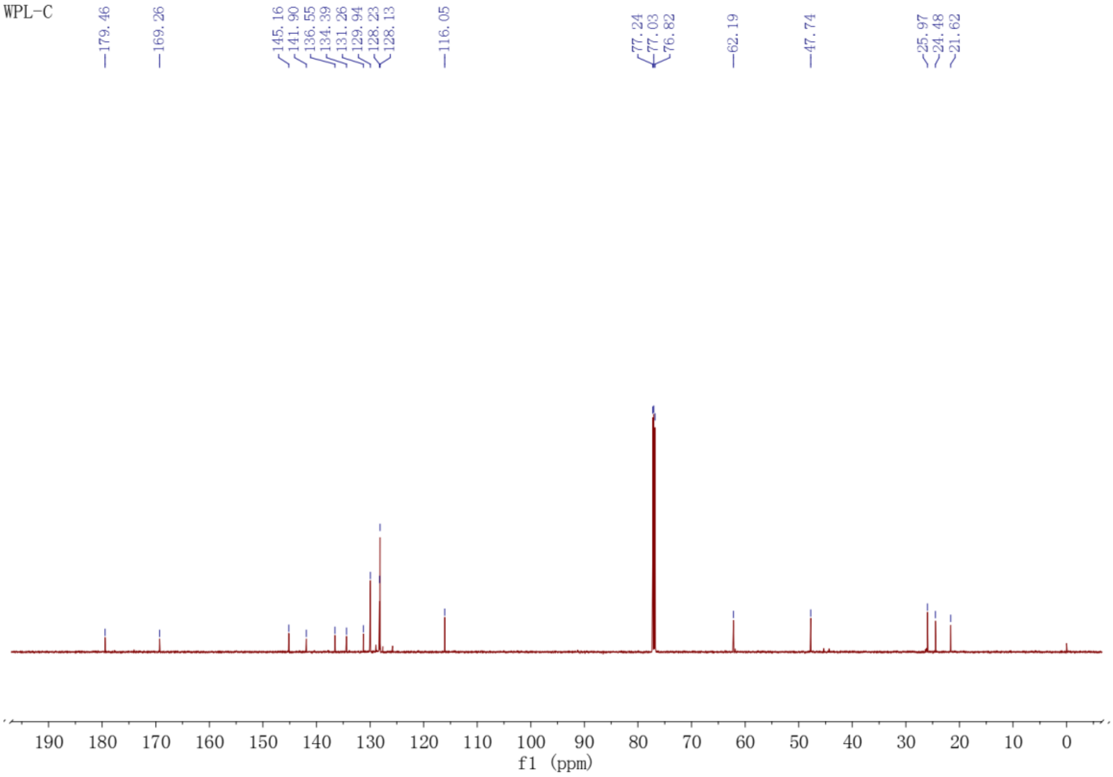
WPL-C
179.46
169.26
145.16
141.90
136.55
134.39
131.26
129.94
128.23
128.13
116.05
77.24
77.03
76.82
62.19
47.74
25.97
24.48
21.62
190 180 170 160 150 140 130 120 110 100 90 80 70 60 50 40 30 20 10 0
f1 (ppm)

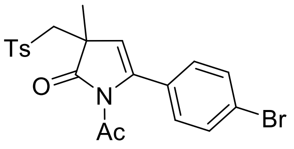
Ts
O
N
Ac
Br

3d

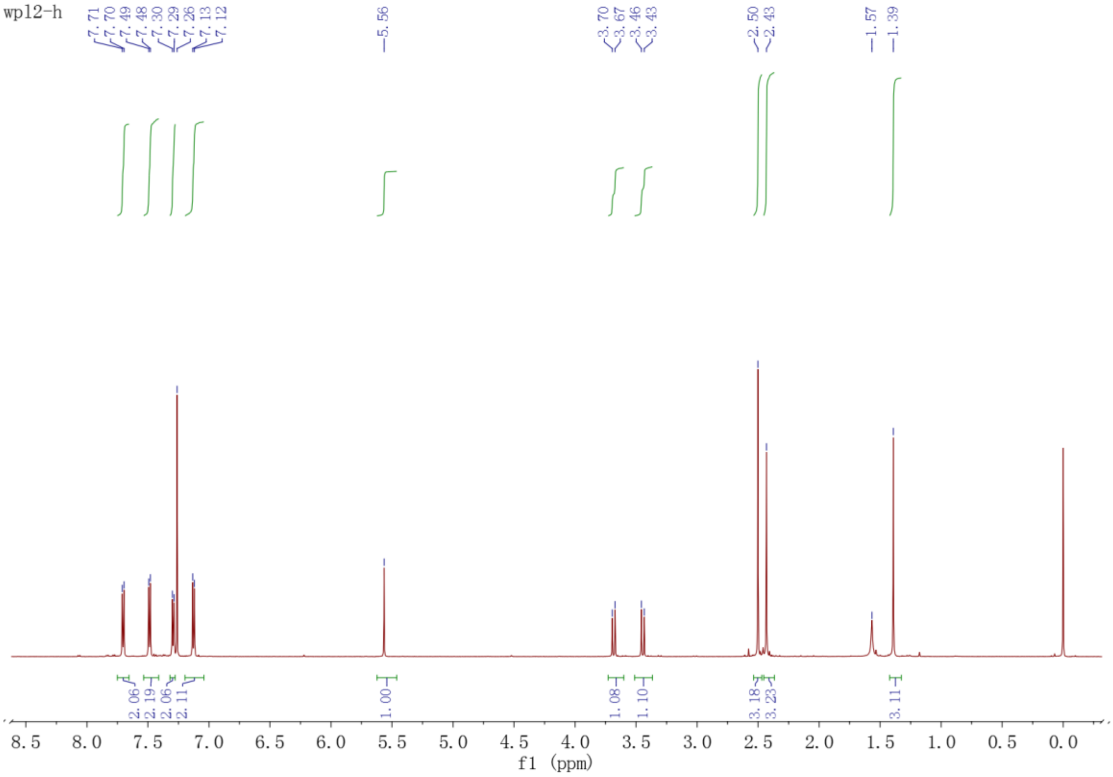
wp12-h
7.71
7.70
7.49
7.48
7.30
7.29
7.26
7.13
7.12
5.56
3.70
3.67
3.46
3.43
2.50
2.43
1.57
1.39
2.06
2.19
2.06
2.11
1.00
1.08
1.10
3.18
3.23
3.11
8.5 8.0 7.5 7.0 6.5 6.0 5.5 5.0 4.5 4.0 3.5 3.0 2.5 2.0 1.5 1.0 0.5 0.0
f1 (ppm)

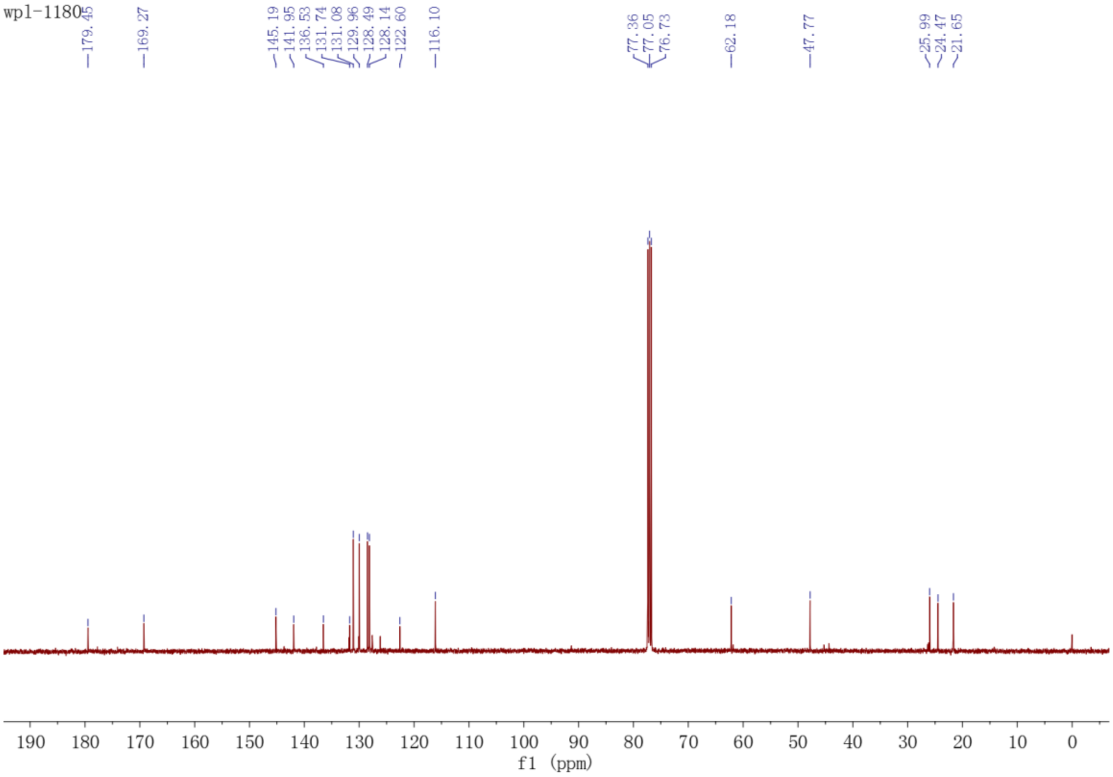
wp1-1180
179.45
169.27
145.19
141.95
136.53
131.74
131.08
129.96
128.49
128.14
122.60
116.10
77.36
77.05
76.73
62.18
47.77
25.99
24.47
21.65
190 180 170 160 150 140 130 120 110 100 90 80 70 60 50 40 30 20 10 0
f1 (ppm)

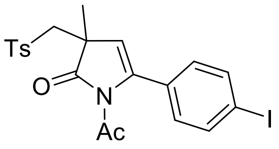

3e

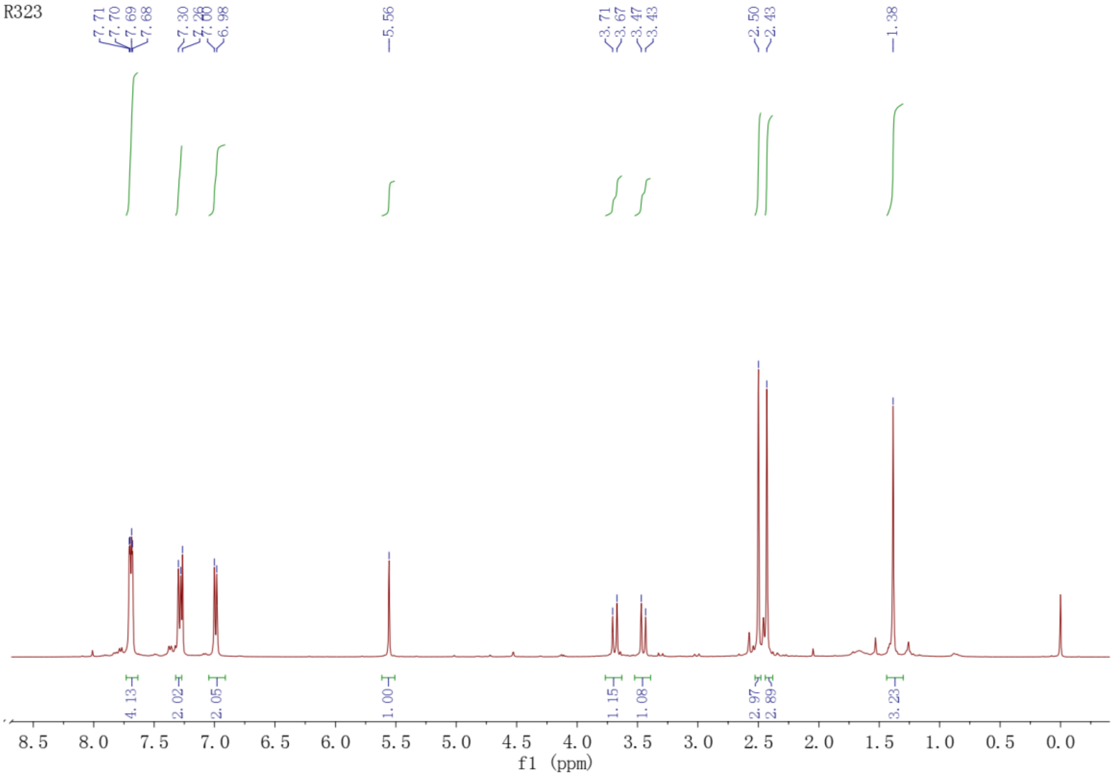
R323
7.71
7.70
7.69
7.68
7.30
7.26
7.00
6.98
5.56
3.71
3.67
3.47
3.43
2.50
2.43
1.38
4.13
2.02
2.05
1.00
1.15
1.08
2.97
2.89
3.23
8.5 8.0 7.5 7.0 6.5 6.0 5.5 5.0 4.5 4.0 3.5 3.0 2.5 2.0 1.5 1.0 0.5 0.0
f1 (ppm)

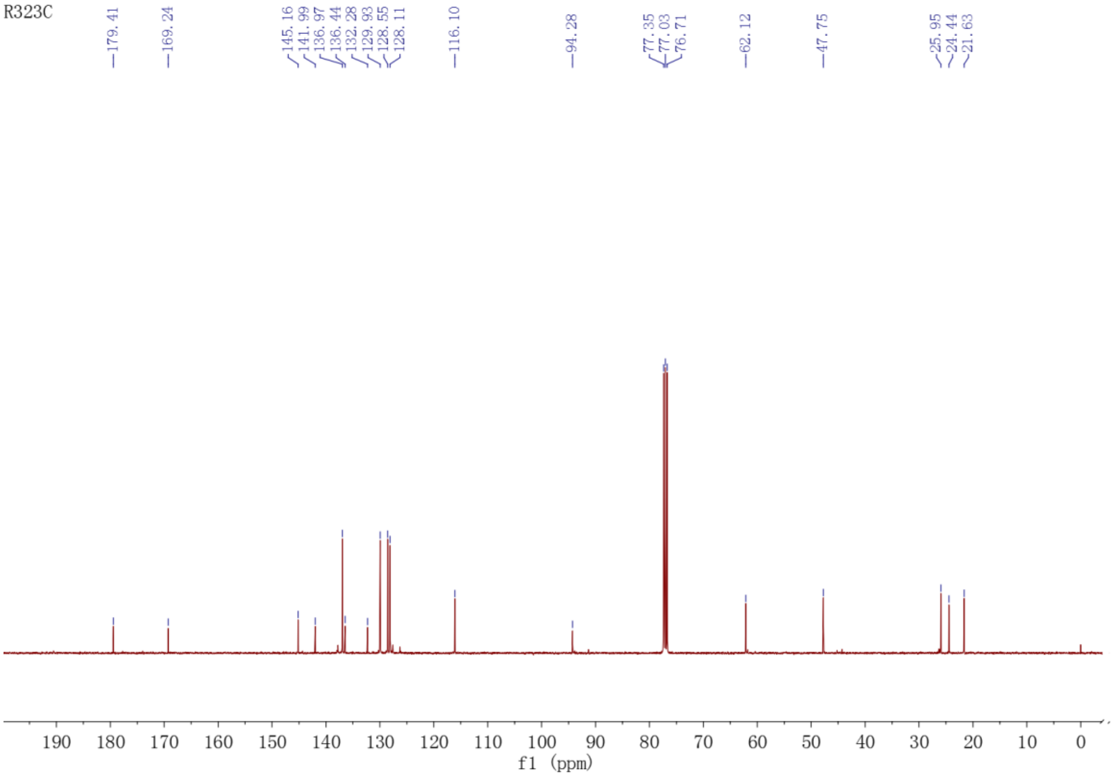
R323C
179.41
169.24
145.16
141.99
136.97
136.44
132.28
129.93
128.55
128.11
116.10
94.28
77.35
77.03
76.71
62.12
47.75
25.95
24.44
21.63
190 180 170 160 150 140 130 120 110 100 90 80 70 60 50 40 30 20 10 0
f1 (ppm)

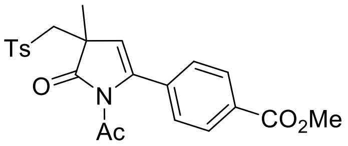
Ts
O
N
Ac
CO2Me

3f

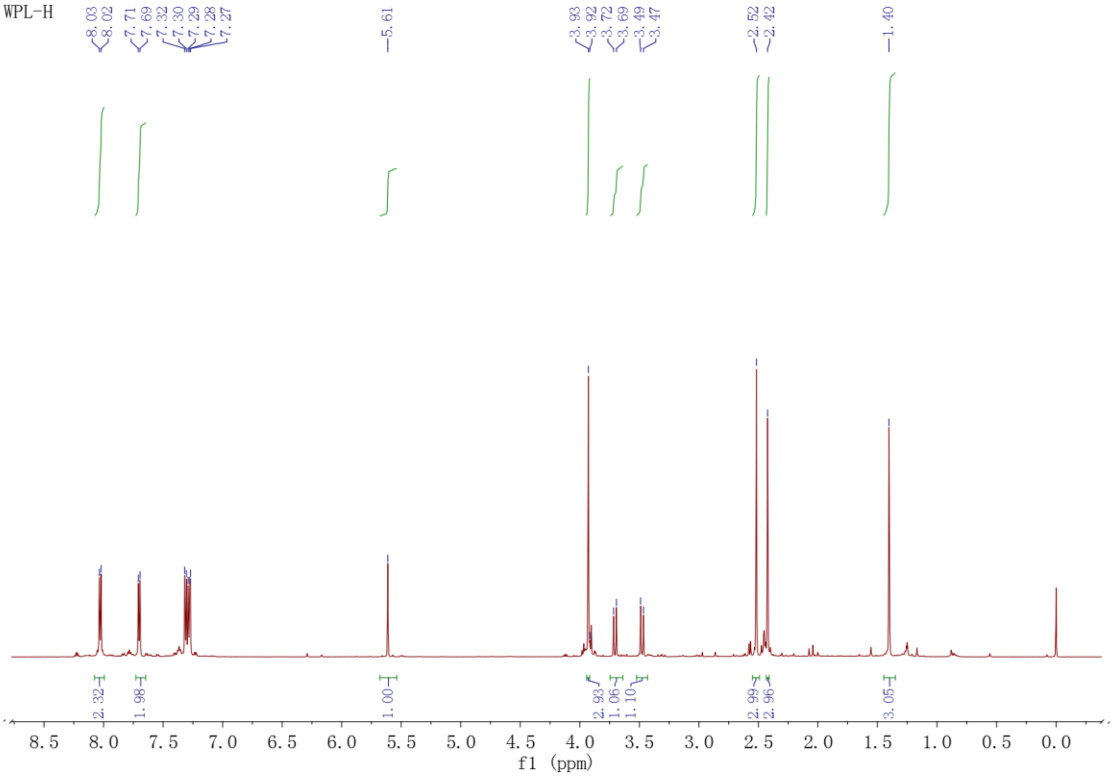
WPL-H
8.03
8.02
7.71
7.69
7.32
7.30
7.29
7.28
7.27
5.61
3.93
3.92
3.72
3.69
3.49
3.47
2.52
2.42
1.40
2.32
1.98
1.00
2.93
1.06
1.10
2.99
2.96
3.05
8.5 8.0 7.5 7.0 6.5 6.0 5.5 5.0 4.5 4.0 3.5 3.0 2.5 2.0 1.5 1.0 0.5 0.0
f1 (ppm)

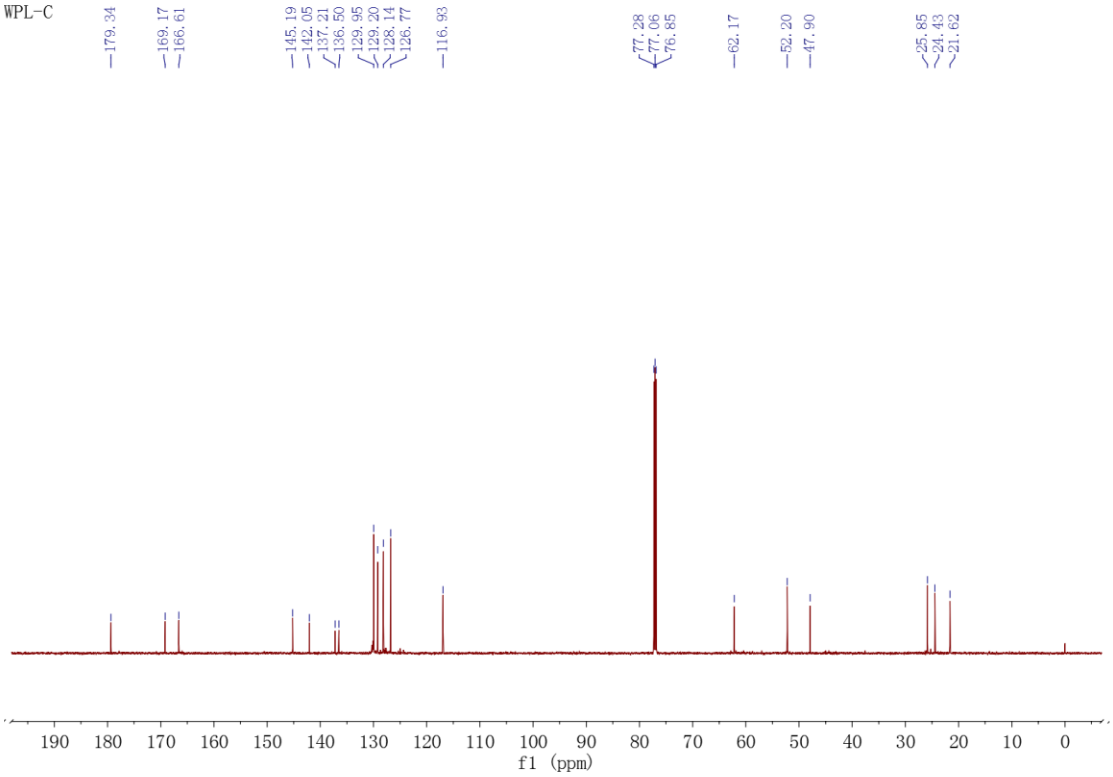
WPL-C
179.34
169.17
166.61
145.19
142.05
137.21
136.50
129.95
129.20
128.14
126.77
116.93
77.28
77.06
76.85
62.17
52.20
47.90
25.85
24.43
21.62
190 180 170 160 150 140 130 120 110 100 90 80 70 60 50 40 30 20 10 0
f1 (ppm)

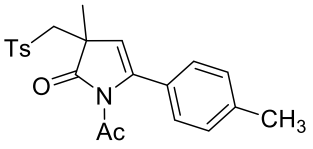
Ts
O
N
Ac
CH3

3g

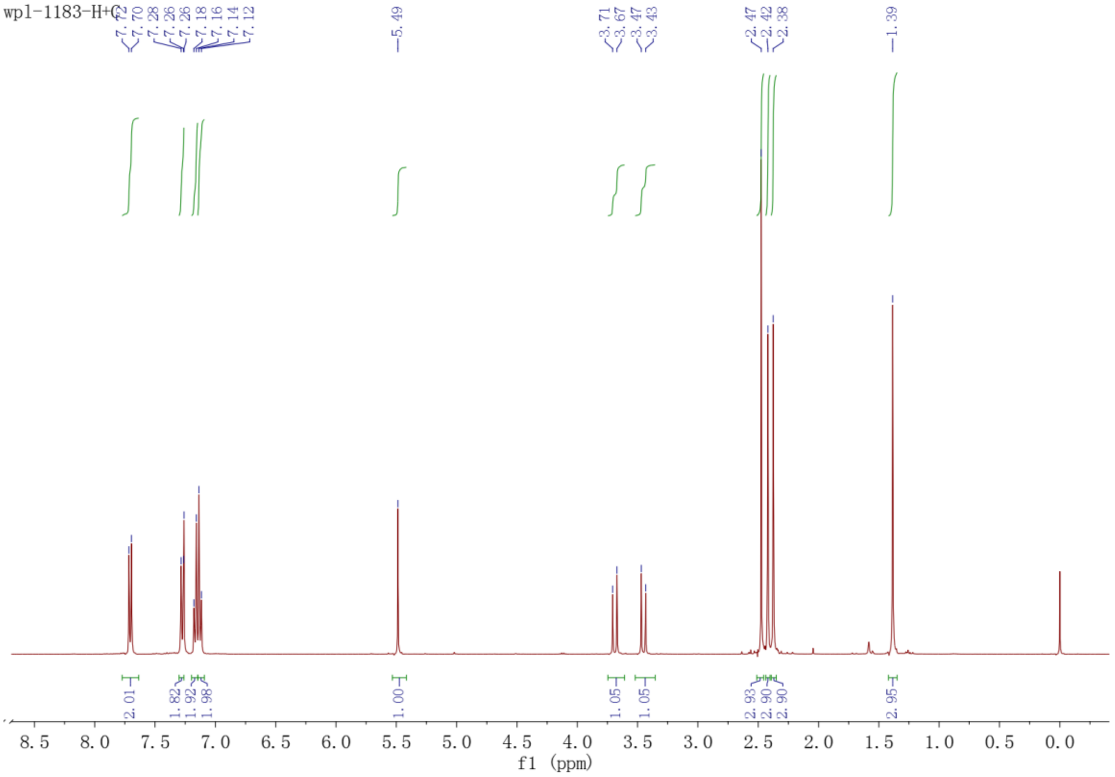
wpl-1183-H+C
7.72
7.70
7.28
7.26
7.26
7.18
7.16
7.14
7.12
5.49
3.71
3.67
3.47
3.43
2.47
2.42
2.38
1.39
2.01
1.82
1.92
1.98
1.00
1.05
1.05
2.93
2.90
2.90
2.95
8.5 8.0 7.5 7.0 6.5 6.0 5.5 5.0 4.5 4.0 3.5 3.0 2.5 2.0 1.5 1.0 0.5 0.0
f1 (ppm)

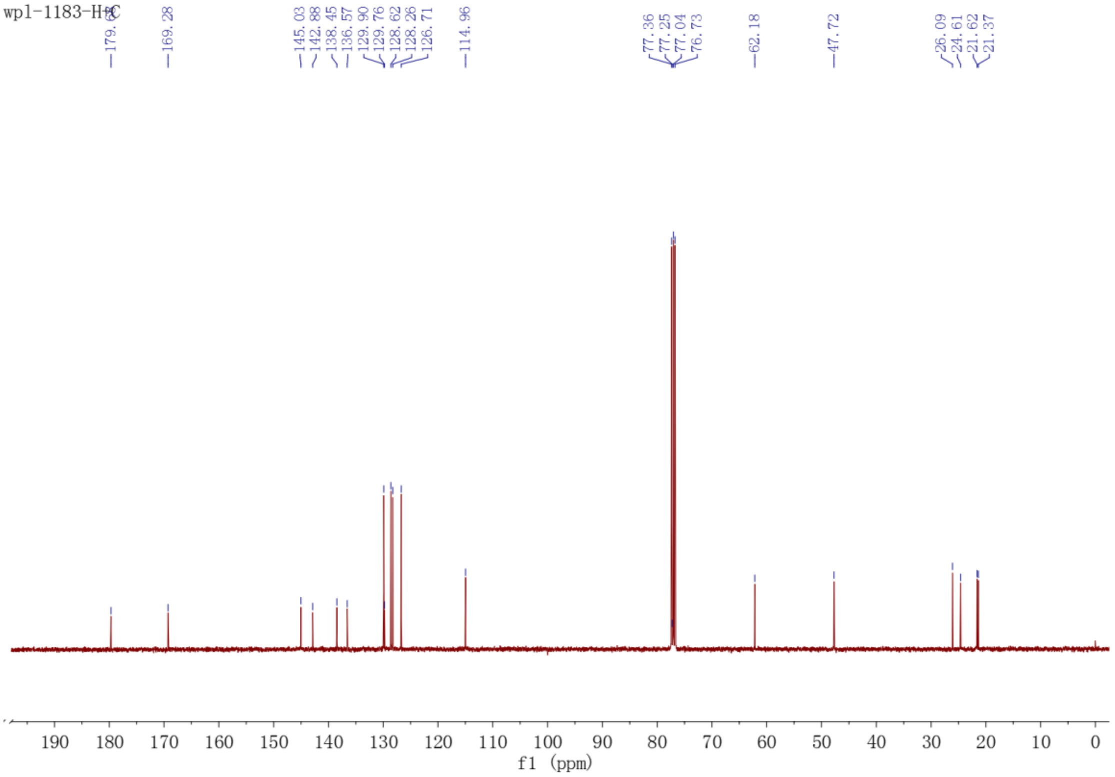
wpl-1183-H+C
179.6
169.28
145.03
142.88
138.45
136.57
129.90
129.76
128.62
128.26
126.71
114.96
77.36
77.25
77.04
76.73
62.18
47.72
26.09
24.61
21.62
21.37
190 180 170 160 150 140 130 120 110 100 90 80 70 60 50 40 30 20 10 0
f1 (ppm)

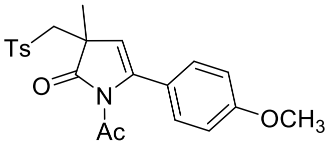
Ts
O
N
Ac
OCH3

3h

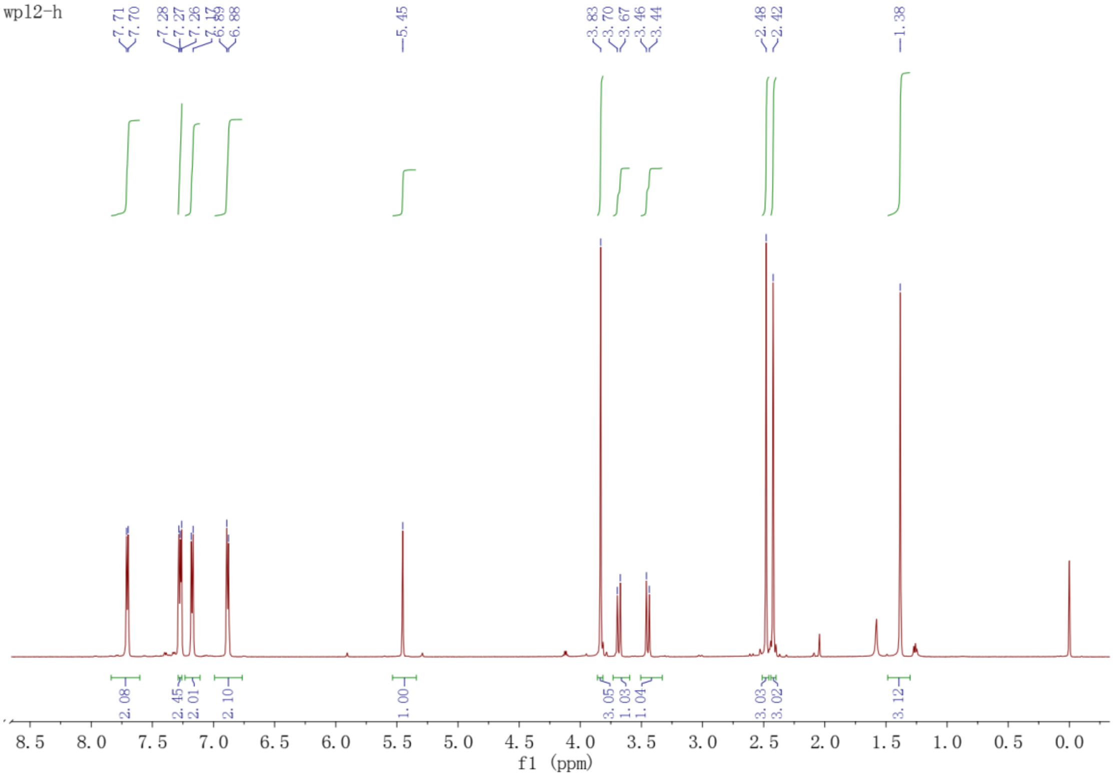
wp12-h
7.71
7.70
7.28
7.27
7.26
7.17
6.89
6.88
5.45
3.83
3.70
3.67
3.46
3.44
2.48
2.42
1.38
2.08
2.45
2.01
2.10
1.00
3.05
1.03
1.04
3.03
3.02
3.12
8.5 8.0 7.5 7.0 6.5 6.0 5.5 5.0 4.5 4.0 3.5 3.0 2.5 2.0 1.5 1.0 0.5 0.0
f1 (ppm)

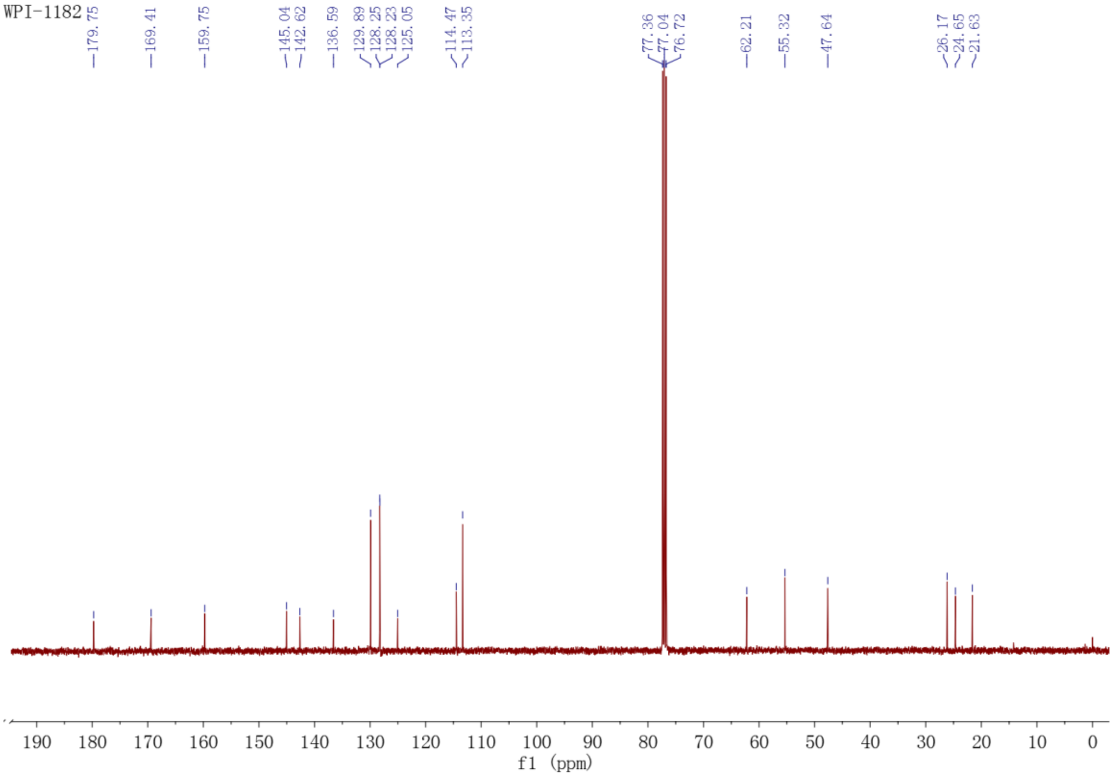
WPI-1182
179.75
169.41
159.75
145.04
142.62
136.59
129.89
128.25
128.23
125.05
114.47
113.35
77.36
77.04
76.72
62.21
55.32
47.64
26.17
24.65
21.63
190 180 170 160 150 140 130 120 110 100 90 80 70 60 50 40 30 20 10 0
f1 (ppm)

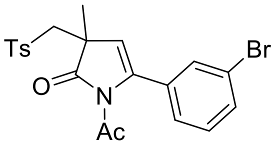
Ts
Br
O
N
Ac

3i

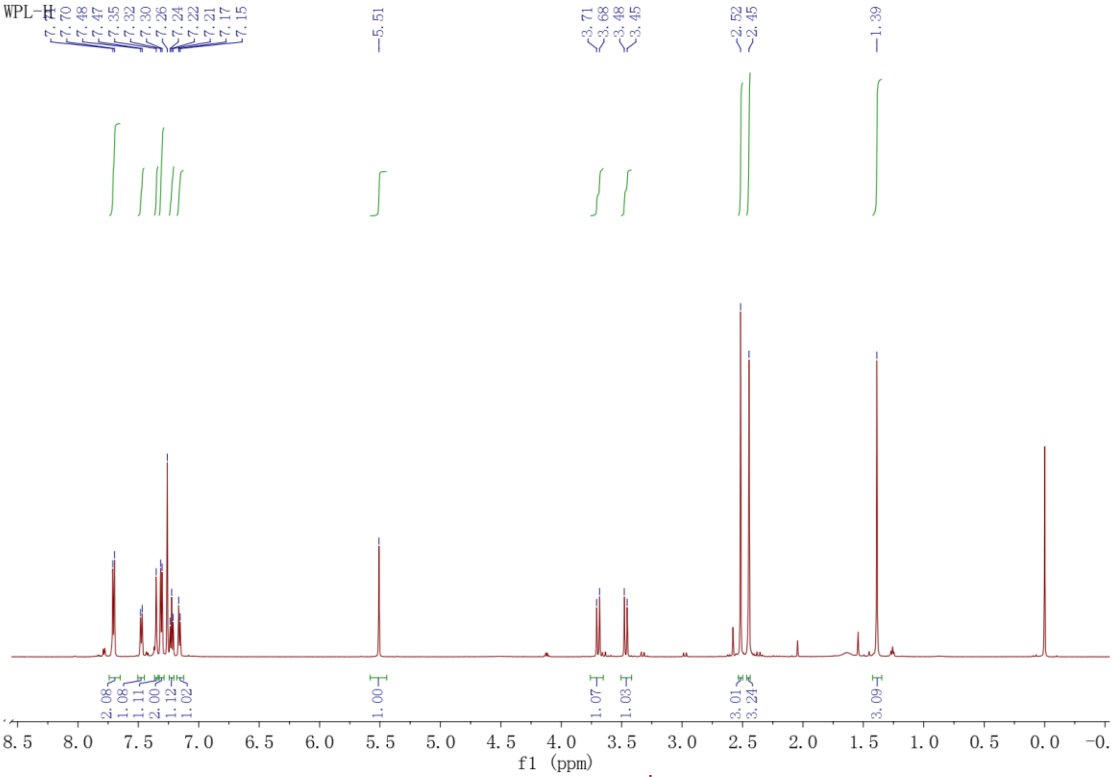
WPL-H
7.70 7.70 7.48 7.47 7.35 7.32 7.30 7.26 7.24 7.22 7.21 7.17 7.15
5.51
3.71 3.68 3.48 3.45
2.52 2.45
1.39
2.08 1.08 1.11 2.00 1.12 1.02
1.00
1.07 1.03
3.01 3.24
3.09
f1 (ppm)

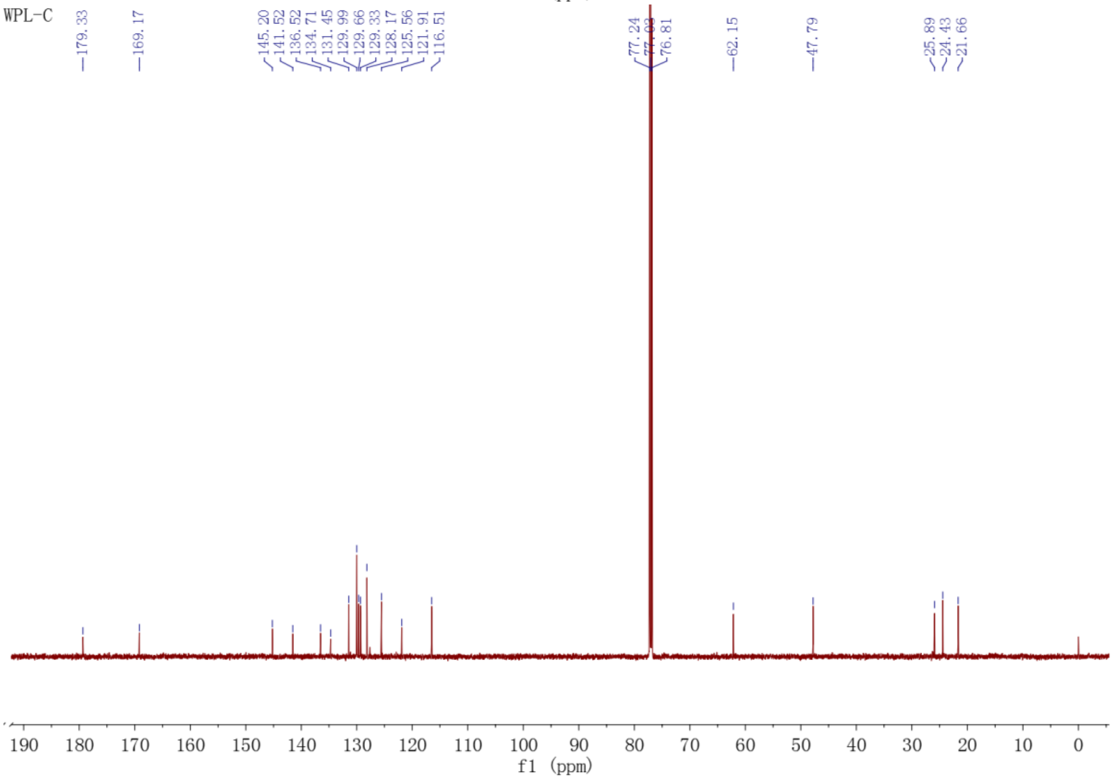
WPL-C
179.33
169.17
145.20 141.52 136.52 134.71 131.45 129.99 129.66 129.33 128.17 125.56 121.91 116.51
77.24 77.03 76.81
62.15
47.79
25.89 24.43 21.66
f1 (ppm)

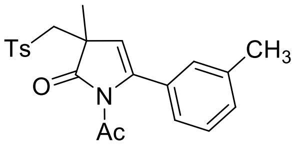
Ts
CH3
O
N
Ac

3j

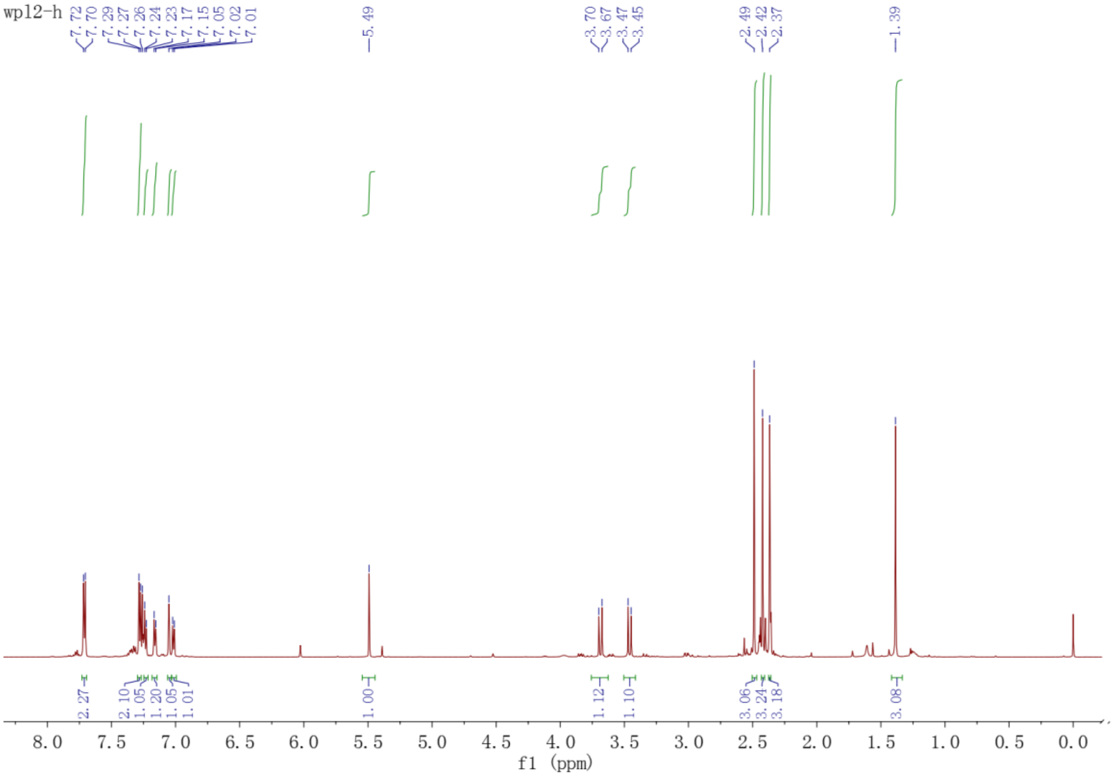
wp12-h
7.72
7.70
7.29
7.27
7.26
7.24
7.23
7.17
7.15
7.05
7.02
7.01
5.49
3.70
3.67
3.47
3.45
2.49
2.42
2.37
1.39
2.27
2.10
1.05
1.20
1.05
1.01
1.00
1.12
1.10
3.06
3.24
3.18
3.08
8.0
7.5
7.0
6.5
6.0
5.5
5.0
4.5
4.0
3.5
3.0
2.5
2.0
1.5
1.0
0.5
0.0
f1 (ppm)

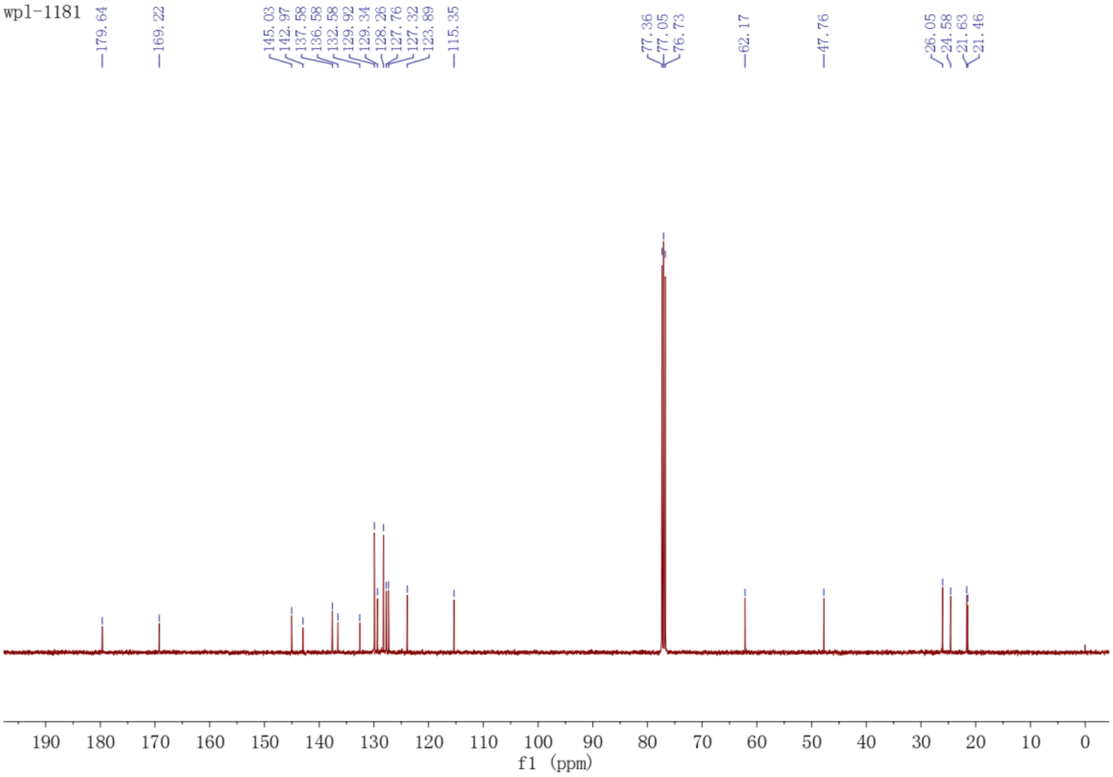
wp1-1181
179.64
169.22
145.03
142.97
137.58
136.58
132.58
129.92
129.34
128.26
127.76
127.32
123.89
115.35
77.36
77.05
76.73
62.17
47.76
26.05
24.58
21.63
21.46
190
180
170
160
150
140
130
120
110
100
90
80
70
60
50
40
30
20
10
0
f1 (ppm)

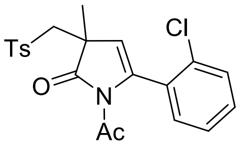
Ts
Cl
O
N
Ac

3k

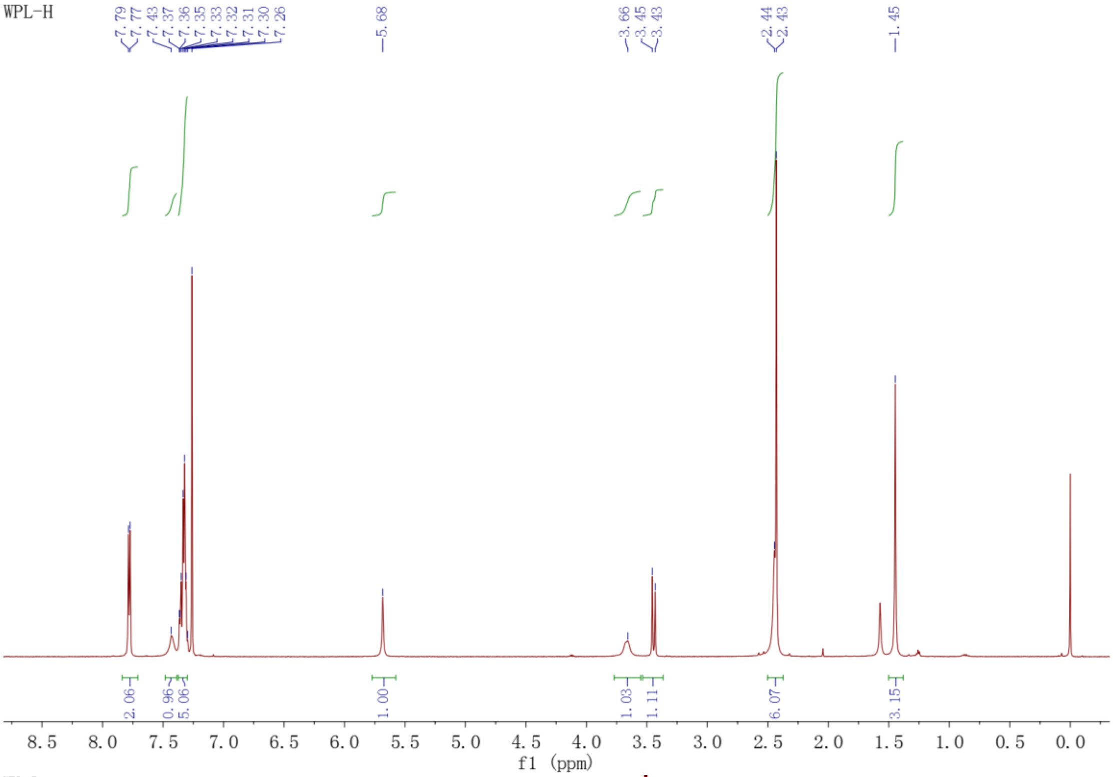
WPL-H
7.79
7.77
7.43
7.37
7.36
7.35
7.33
7.32
7.31
7.30
7.26
5.68
3.66
3.45
3.43
2.44
2.43
1.45
2.06
0.96
5.06
1.00
1.03
1.11
6.07
3.15
8.5 8.0 7.5 7.0 6.5 6.0 5.5 5.0 4.5 4.0 3.5 3.0 2.5 2.0 1.5 1.0 0.5 0.0
f1 (ppm)

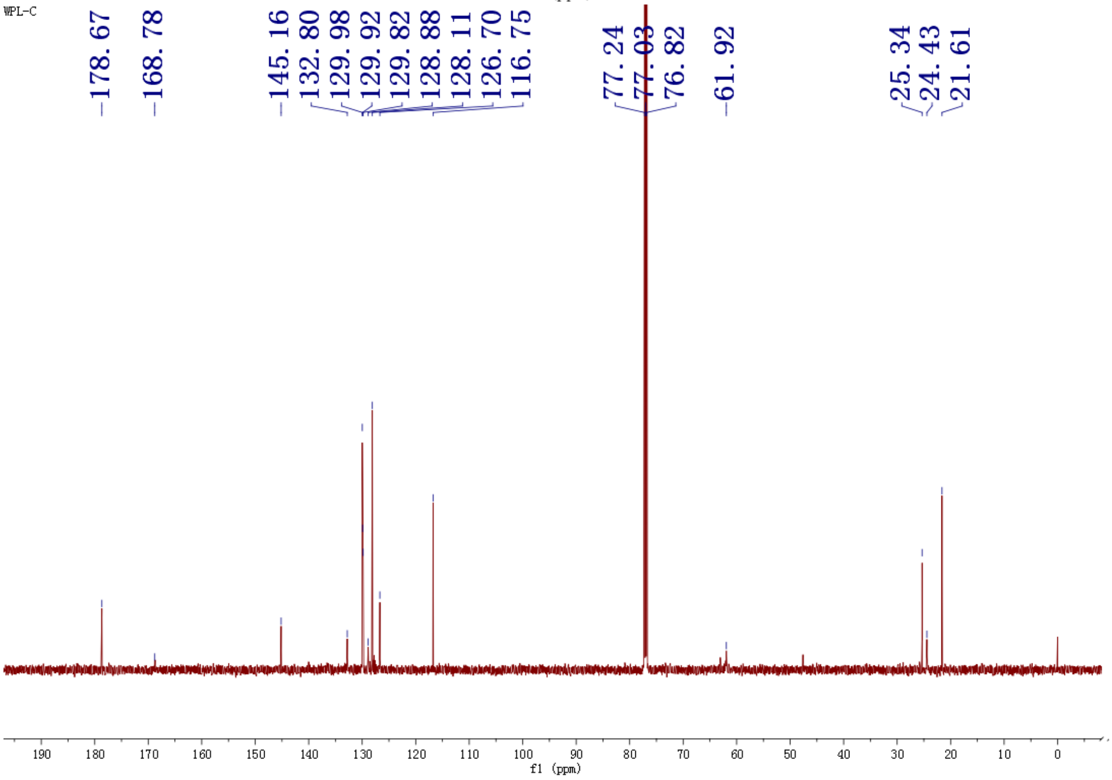
WPL-C
178.67
168.78
145.16
132.80
129.98
129.92
129.82
128.88
128.11
126.70
116.75
77.24
77.03
76.82
61.92
25.34
24.43
21.61
190 180 170 160 150 140 130 120 110 100 90 80 70 60 50 40 30 20 10 0
f1 (ppm)

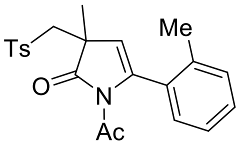
Ts
Me
O
N
Ac

3l

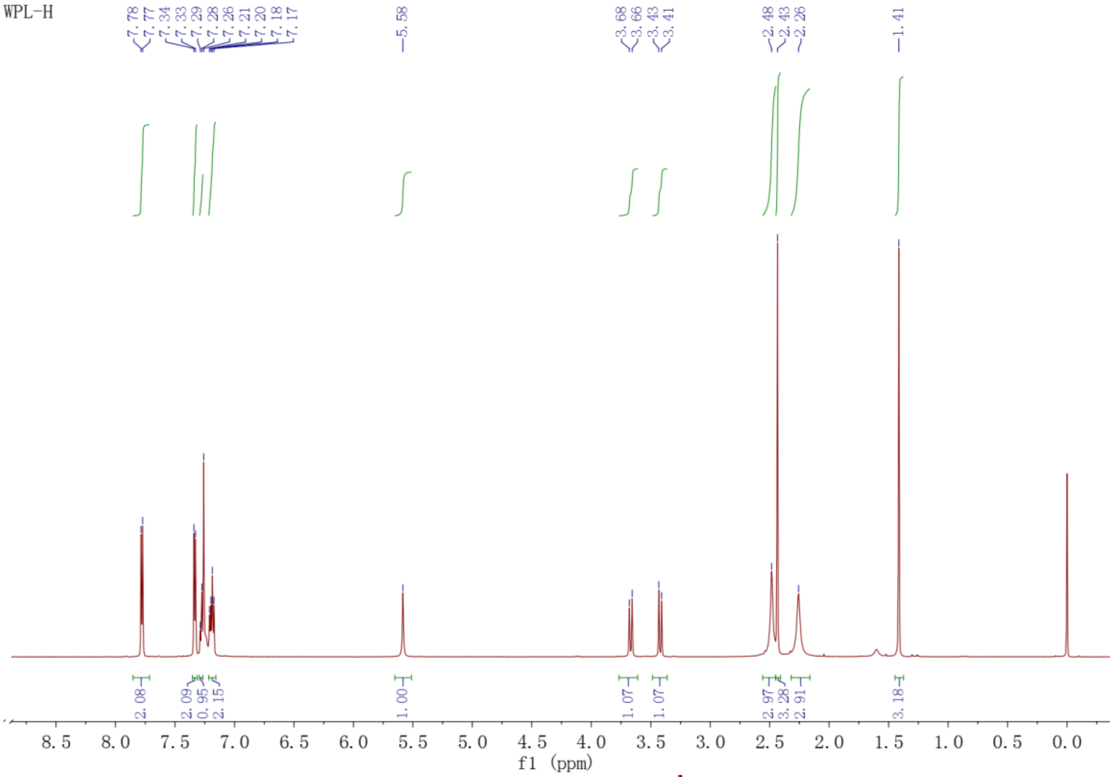
WPL-H
7.78
7.77
7.34
7.33
7.29
7.28
7.26
7.21
7.20
7.18
7.17
5.58
3.68
3.66
3.43
3.41
2.48
2.43
2.26
1.41
2.08
2.09
0.95
2.15
1.00
1.07
1.07
2.97
3.28
2.91
3.18
8.5 8.0 7.5 7.0 6.5 6.0 5.5 5.0 4.5 4.0 3.5 3.0 2.5 2.0 1.5 1.0 0.5 0.0
f1 (ppm)

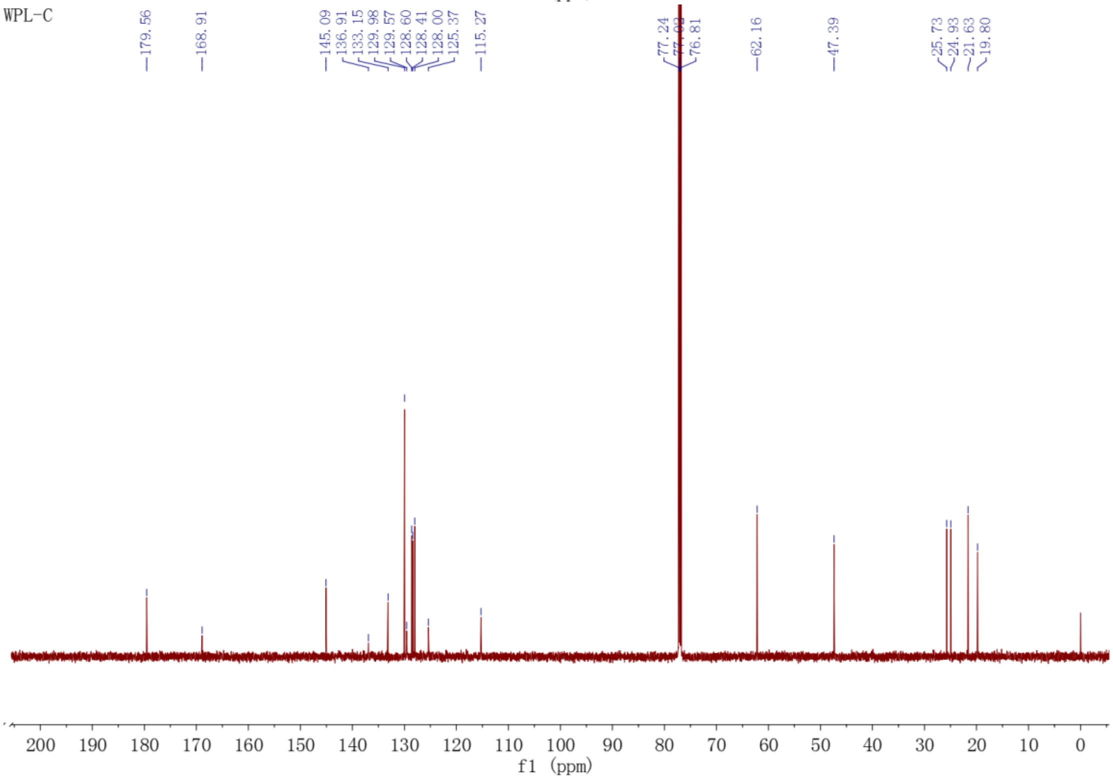
WPL-C
179.56
168.91
145.09
136.91
133.15
129.98
129.57
128.60
128.41
128.00
125.37
115.27
77.24
77.02
76.81
62.16
47.39
25.73
24.93
21.63
19.80
200 190 180 170 160 150 140 130 120 110 100 90 80 70 60 50 40 30 20 10 0
f1 (ppm)

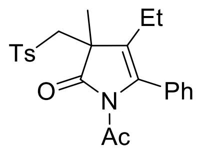
Ts
Et
O
N
Ph
Ac

3m

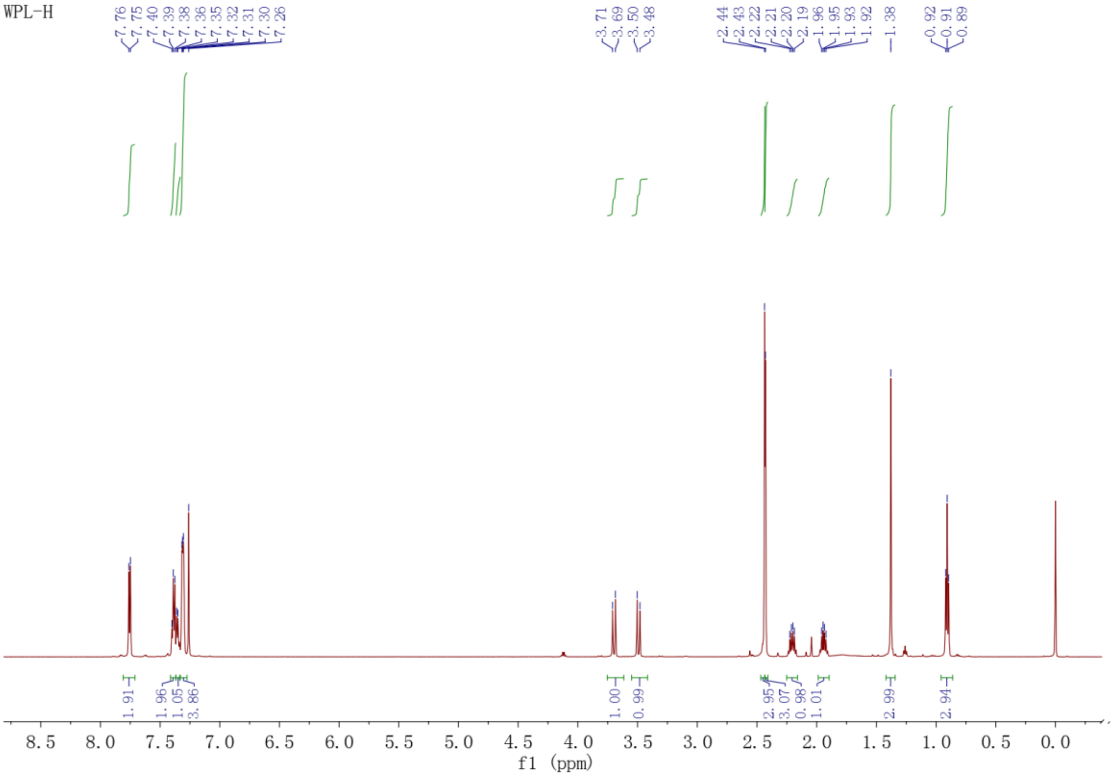
WPL-H
7.76
7.75
7.40
7.39
7.38
7.36
7.35
7.32
7.31
7.30
7.26
3.71
3.69
3.50
3.48
2.44
2.43
2.22
2.21
2.20
2.19
1.96
1.95
1.93
1.92
1.38
0.92
0.91
0.89
1.91
1.96
1.05
3.86
1.00
0.99
2.95
3.07
0.98
1.01
2.99
2.94
8.5
8.0
7.5
7.0
6.5
6.0
5.5
5.0
4.5
4.0
3.5
3.0
2.5
2.0
1.5
1.0
0.5
0.0
f1 (ppm)

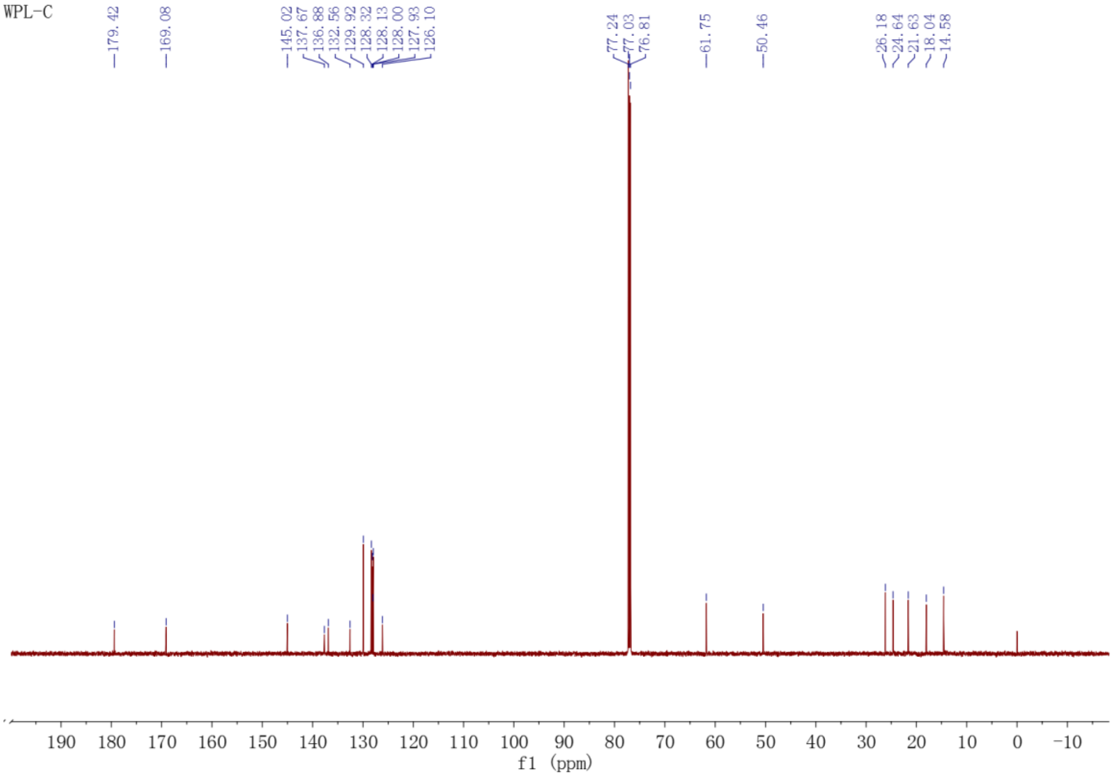
WPL-C
179.42
169.08
145.02
137.67
136.88
132.56
129.92
128.32
128.13
128.00
127.93
126.10
77.24
77.03
76.81
61.75
50.46
26.18
24.64
21.63
18.04
14.58
190
180
170
160
150
140
130
120
110
100
90
80
70
60
50
40
30
20
10
0
-10
f1 (ppm)

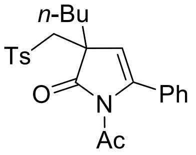

3n

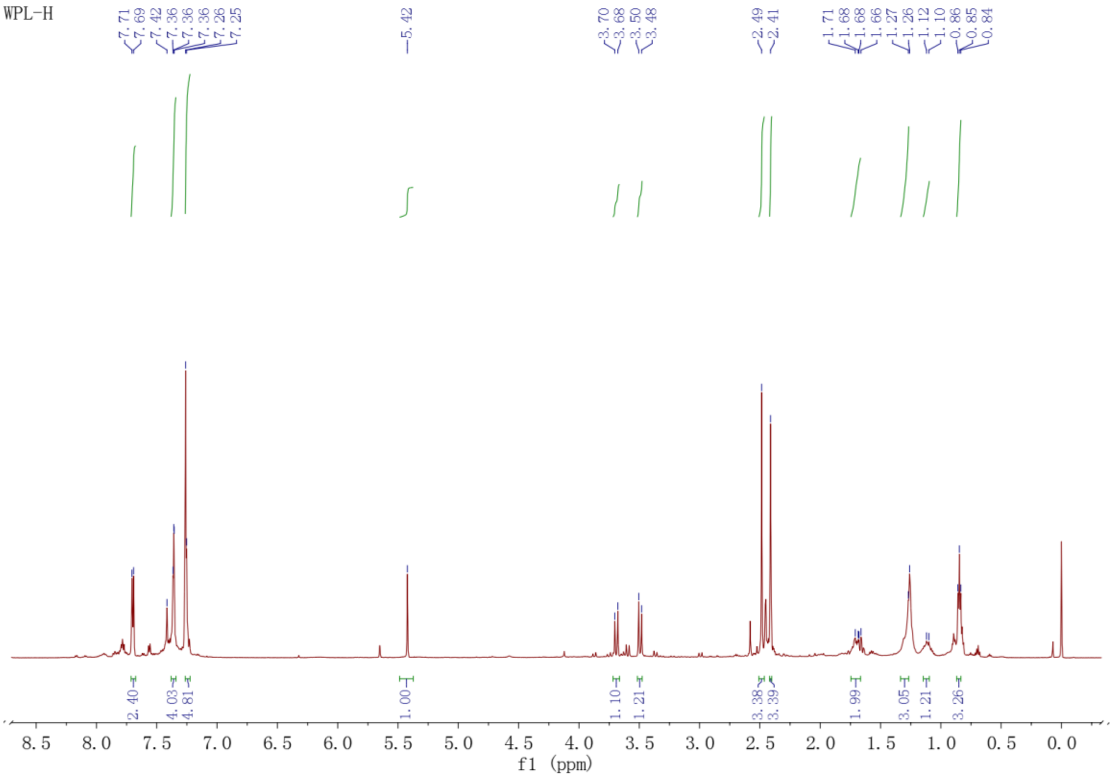
WPL-H
7.71
7.69
7.42
7.36
7.36
7.36
7.26
7.25
5.42
3.70
3.68
3.50
3.48
2.49
2.41
1.71
1.68
1.68
1.66
1.27
1.26
1.12
1.10
0.86
0.85
0.84
2.40
4.03
4.81
1.00
1.10
1.21
3.38
3.39
1.99
3.05
1.21
3.26
8.5 8.0 7.5 7.0 6.5 6.0 5.5 5.0 4.5 4.0 3.5 3.0 2.5 2.0 1.5 1.0 0.5 0.0
f1 (ppm)

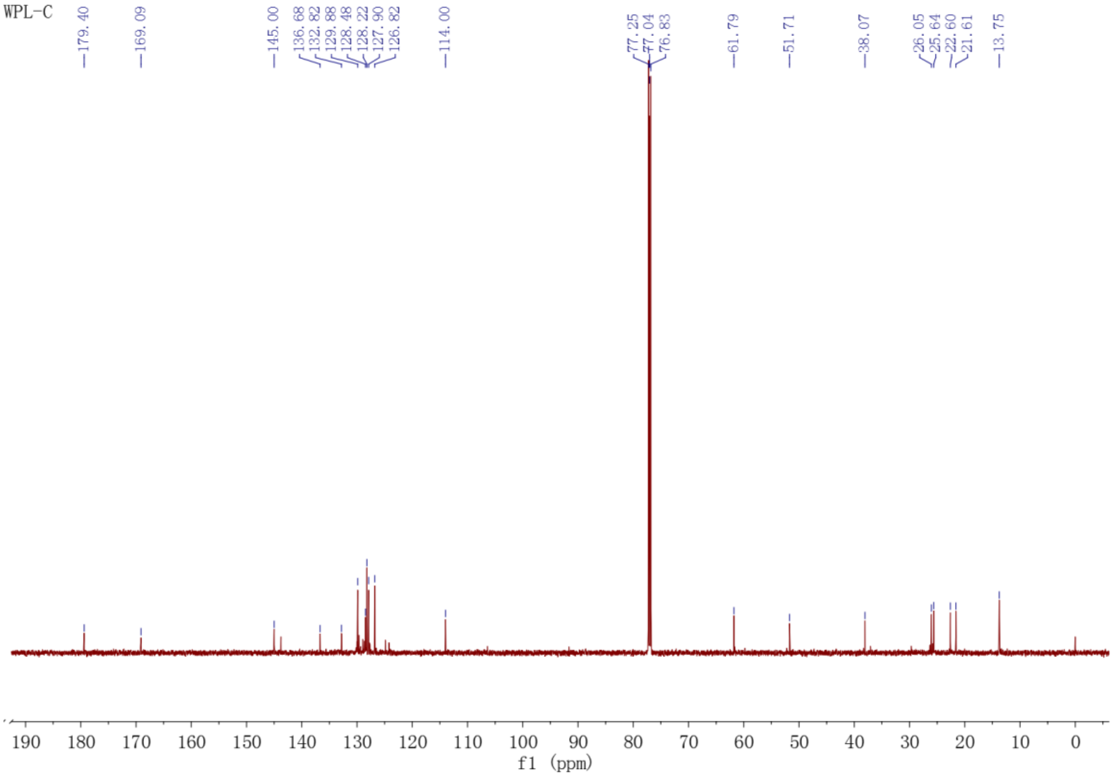
WPL-C
179.40
169.09
145.00
136.68
132.82
129.88
128.48
128.22
127.90
126.82
114.00
77.25
77.04
76.83
61.79
51.71
38.07
26.05
25.64
22.60
21.61
13.75
190 180 170 160 150 140 130 120 110 100 90 80 70 60 50 40 30 20 10 0
f1 (ppm)

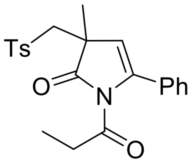
Ts
O
N
Ph
O

3o

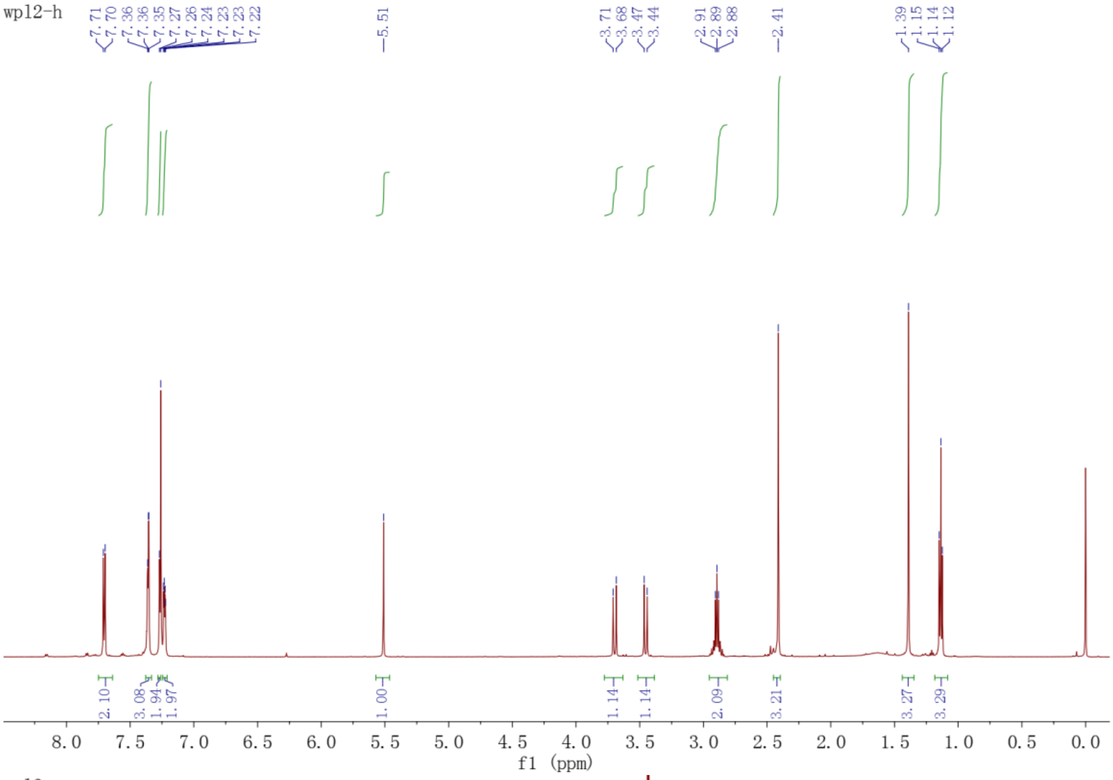
wp12-h
7.71
7.70
7.36
7.36
7.35
7.27
7.26
7.24
7.23
7.23
7.22
5.51
3.71
3.68
3.47
3.44
2.91
2.89
2.88
2.41
1.39
1.15
1.14
1.12
2.10
3.08
1.94
1.97
1.00
1.14
1.14
2.09
3.21
3.27
3.29
8.0
7.5
7.0
6.5
6.0
5.5
5.0
4.5
4.0
3.5
3.0
2.5
2.0
1.5
1.0
0.5
0.0
f1 (ppm)

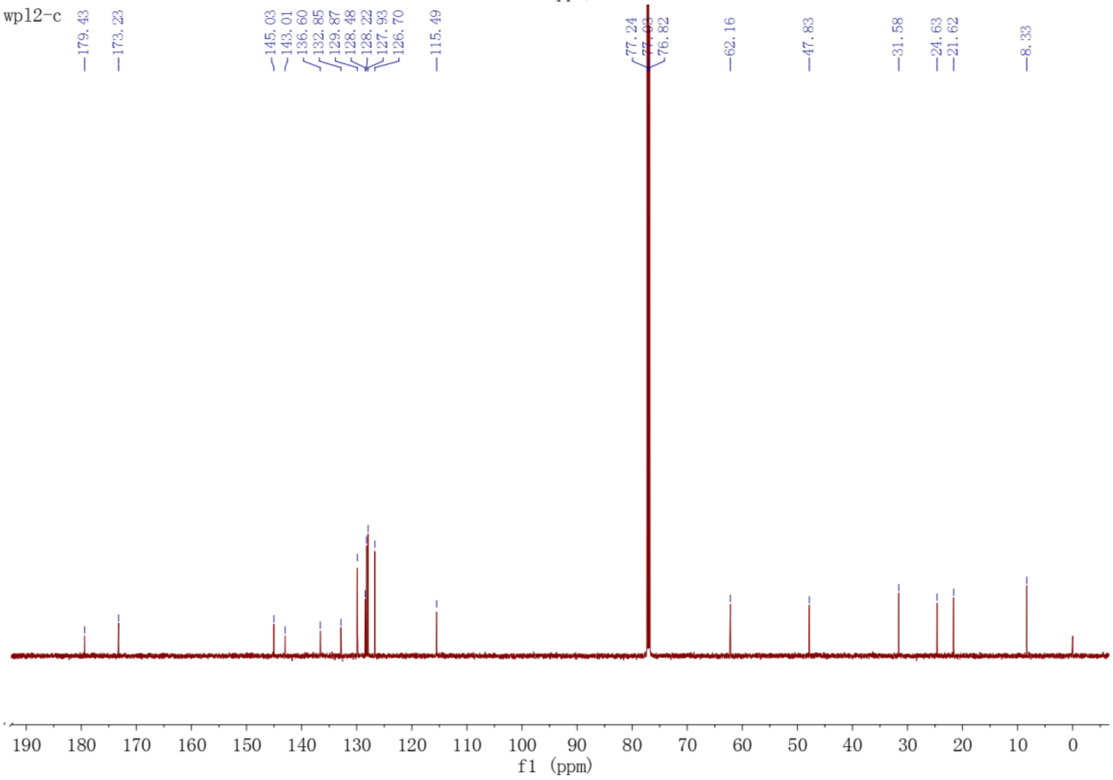
wp12-c
179.43
173.23
145.03
143.01
136.60
132.85
129.87
128.48
128.22
127.93
126.70
115.49
77.24
76.82
62.16
47.83
31.58
24.63
21.62
8.33
190
180
170
160
150
140
130
120
110
100
90
80
70
60
50
40
30
20
10
0
f1 (ppm)

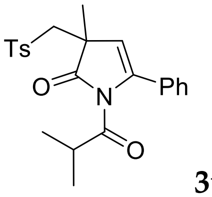
Ts
O
N
Ph
O

3p

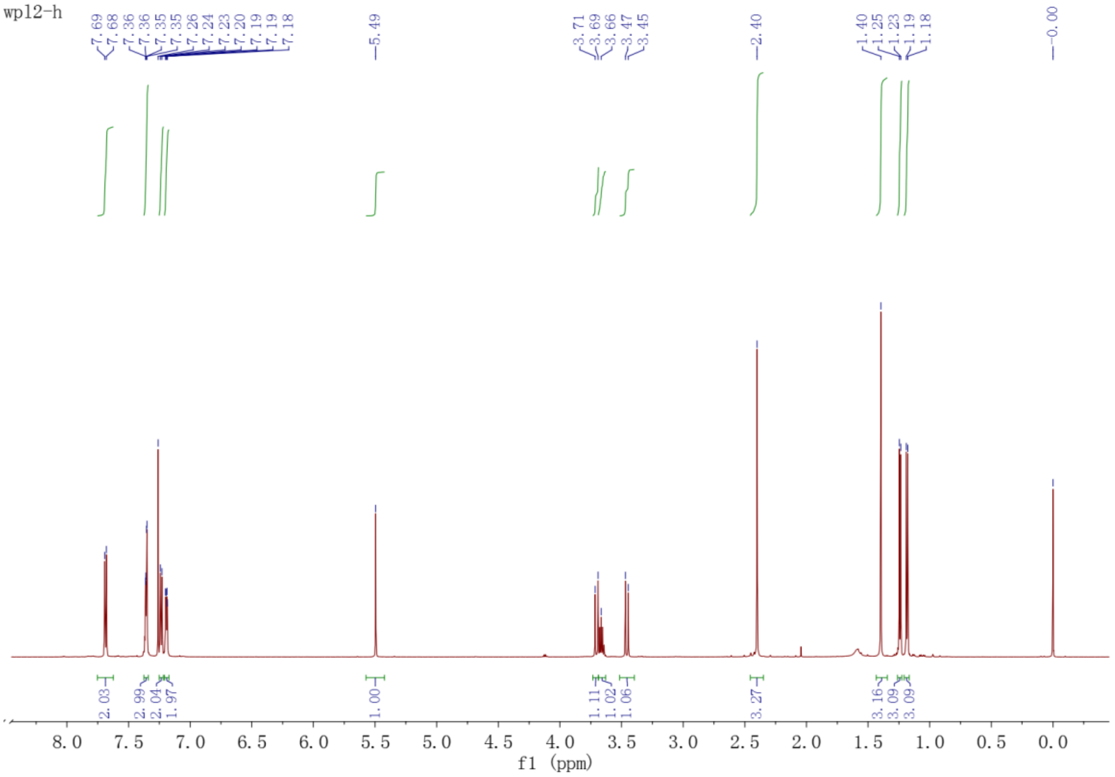
wp12-h
7.69
7.68
7.36
7.36
7.35
7.35
7.26
7.24
7.23
7.20
7.19
7.19
7.18
5.49
3.71
3.69
3.66
3.47
3.45
2.40
1.40
1.25
1.23
1.19
1.18
0.00
2.03
2.99
2.04
1.97
1.00
1.11
1.02
1.06
3.27
3.16
3.09
3.09
8.0 7.5 7.0 6.5 6.0 5.5 5.0 4.5 4.0 3.5 3.0 2.5 2.0 1.5 1.0 0.5 0.0
f1 (ppm)

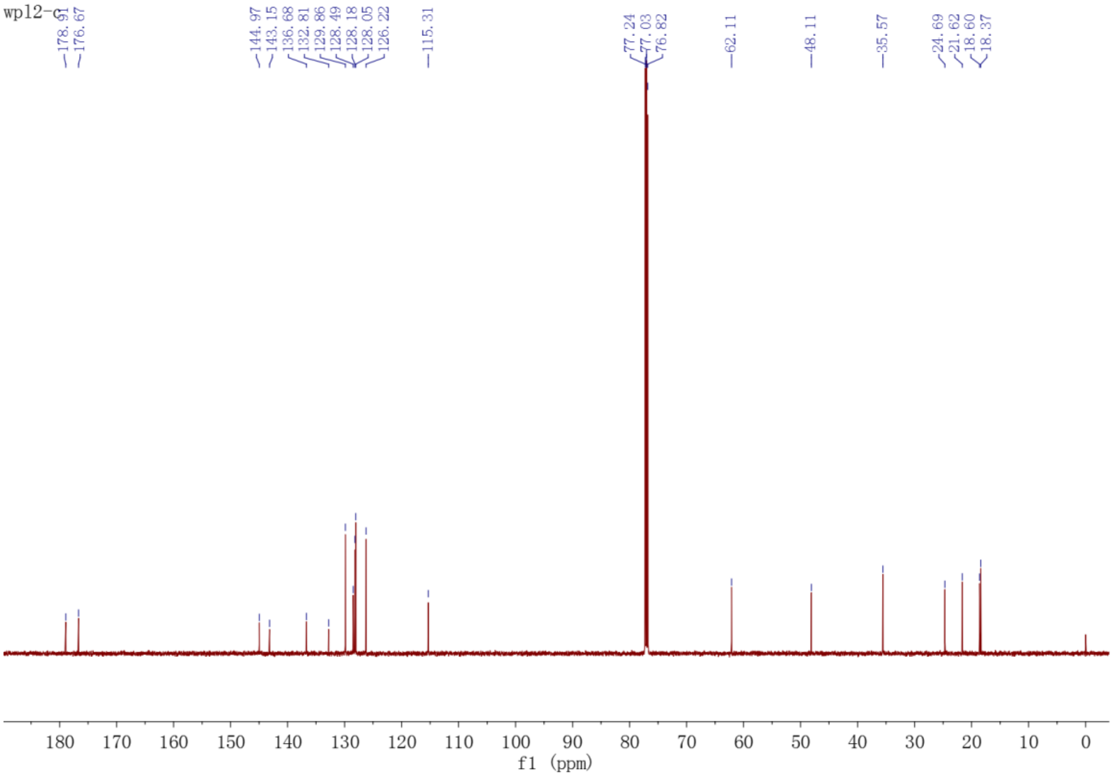
wp12-c
178.91
176.67
144.97
143.15
136.68
132.81
129.86
128.49
128.18
128.05
126.22
115.31
77.24
77.03
76.82
62.11
48.11
35.57
24.69
21.62
18.60
18.37
180 170 160 150 140 130 120 110 100 90 80 70 60 50 40 30 20 10 0
f1 (ppm)

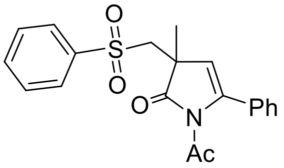
O
S
O
O
N
Ph
Ac

4a

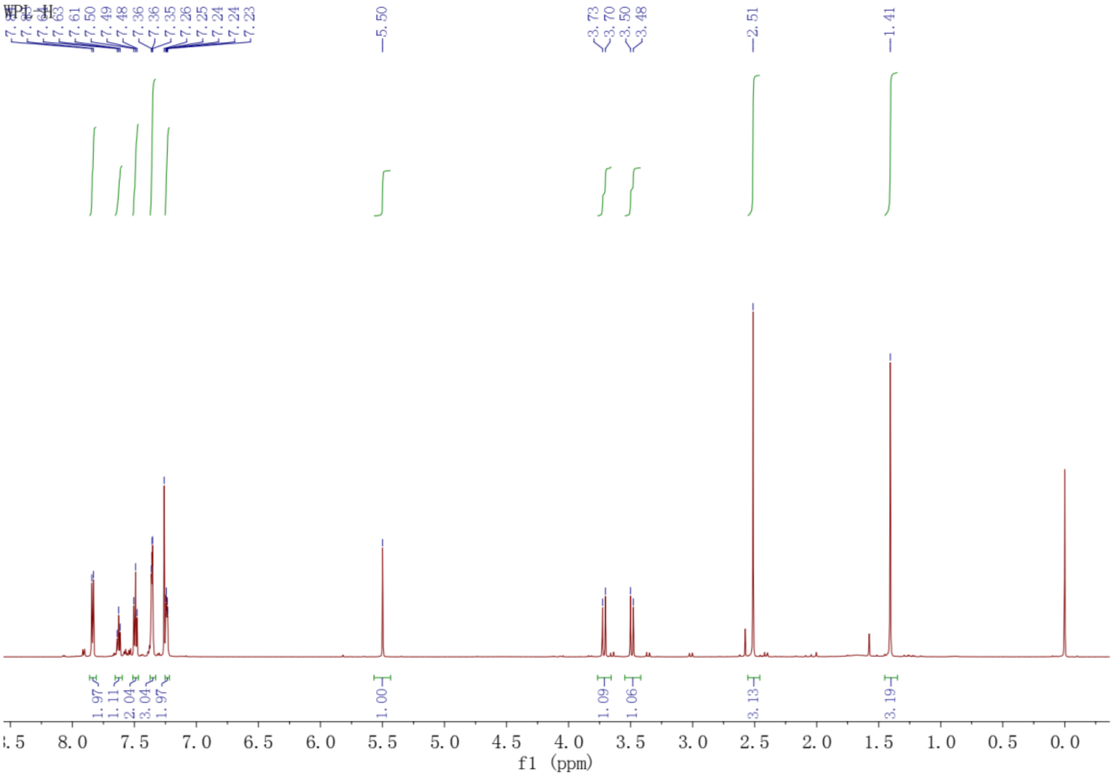
WPL-H
7.63
7.61
7.50
7.49
7.48
7.36
7.36
7.35
7.26
7.25
7.24
7.24
7.23
5.50
3.73
3.70
3.50
3.48
2.51
1.41
1.97
1.11
2.04
3.04
1.97
1.00
1.09
1.06
3.13
3.19
8.0 7.5 7.0 6.5 6.0 5.5 5.0 4.5 4.0 3.5 3.0 2.5 2.0 1.5 1.0 0.5 0.0
f1 (ppm)

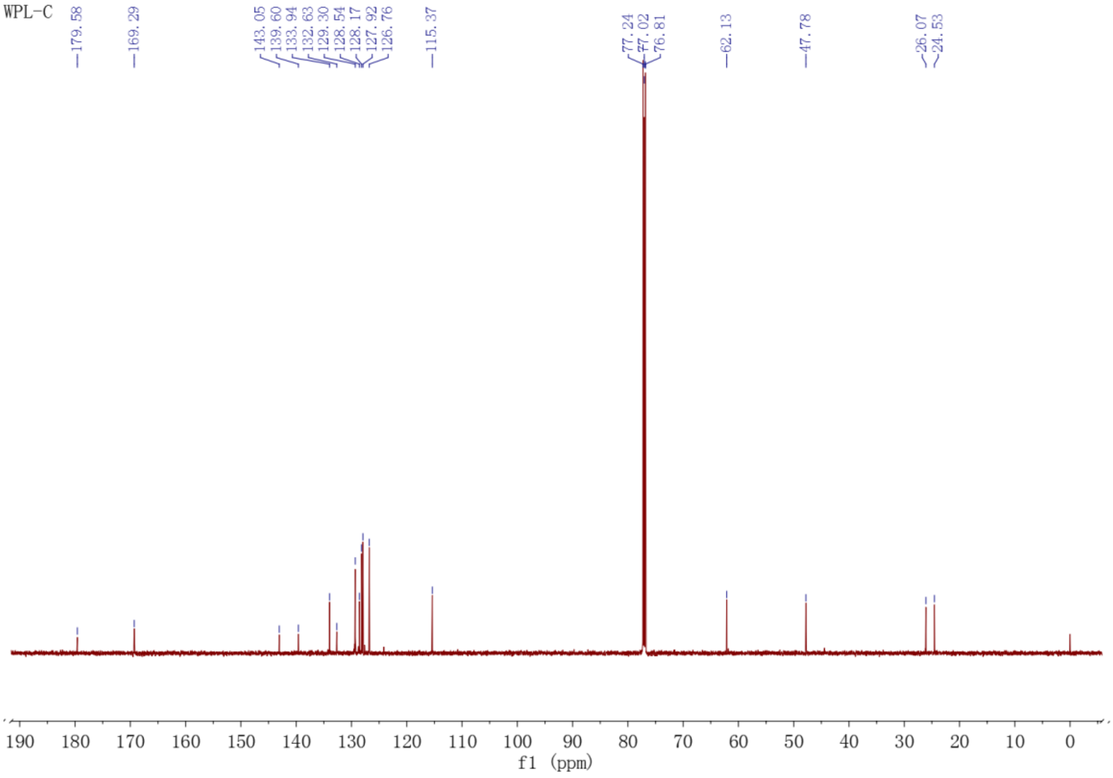
WPL-C
179.58
169.29
143.05
139.60
133.94
132.63
129.30
128.54
128.17
127.92
126.76
115.37
77.24
77.02
76.81
62.13
47.78
26.07
24.53
190 180 170 160 150 140 130 120 110 100 90 80 70 60 50 40 30 20 10 0
f1 (ppm)

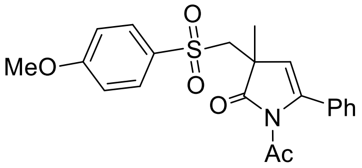
MeO
O
S
O
O
N
Ph
Ac

4b

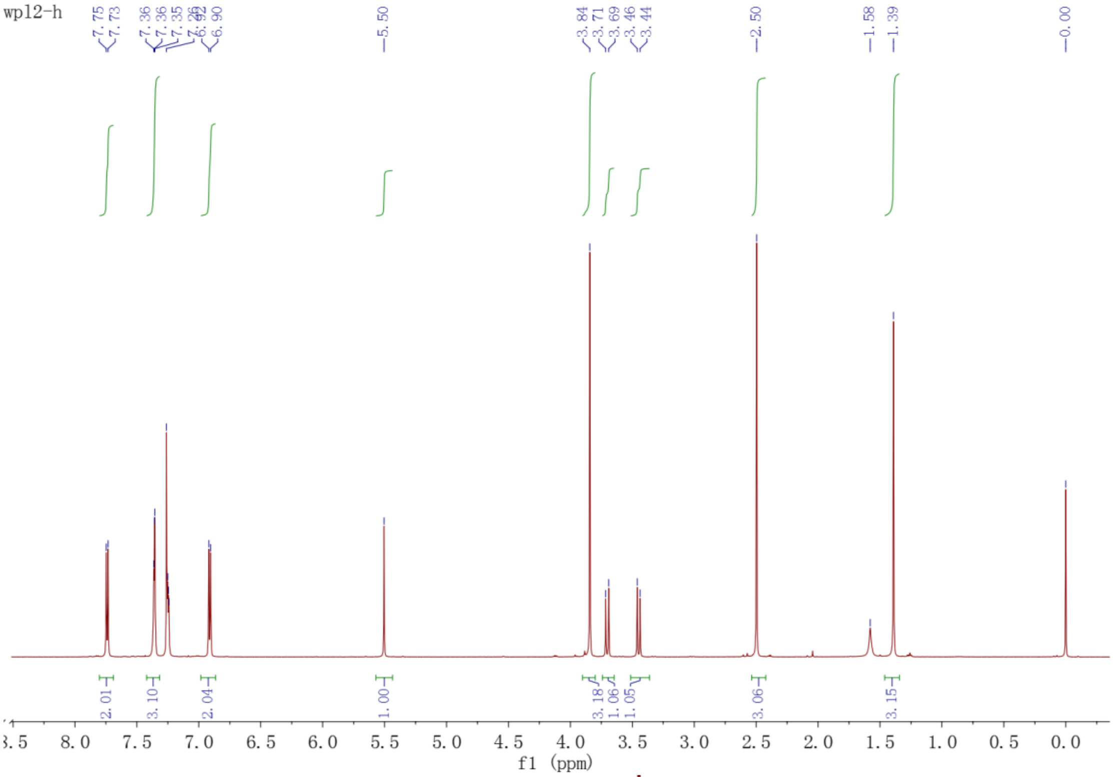
wp12-h
7.75
7.73
7.36
7.36
7.35
7.26
6.92
6.90
5.50
3.84
3.71
3.69
3.46
3.44
2.50
1.58
1.39
0.00
2.01
3.10
2.04
1.00
3.18
1.06
1.05
3.06
3.15
8.0 7.5 7.0 6.5 6.0 5.5 5.0 4.5 4.0 3.5 3.0 2.5 2.0 1.5 1.0 0.5 0.0
f1 (ppm)

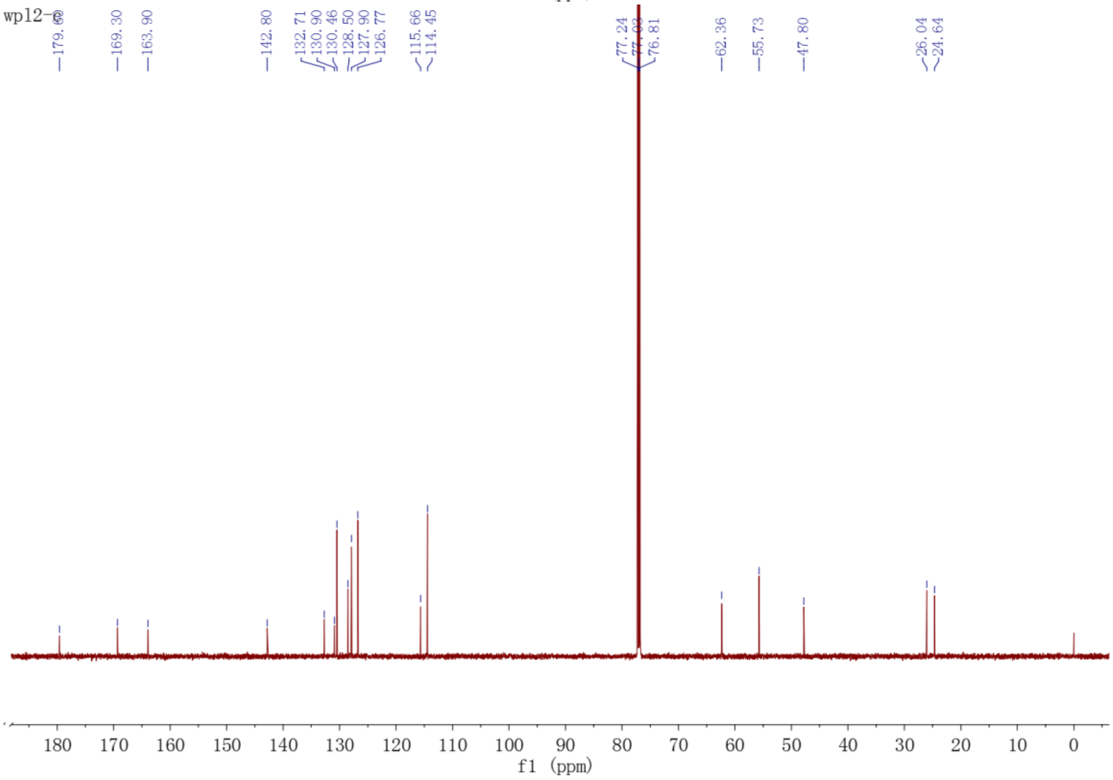
169.30
163.90
142.80
132.71
130.90
130.46
128.50
127.90
126.77
115.66
114.45
77.24
76.81
62.36
55.73
47.80
26.04
24.64
180 170 160 150 140 130 120 110 100 90 80 70 60 50 40 30 20 10 0
f1 (ppm)

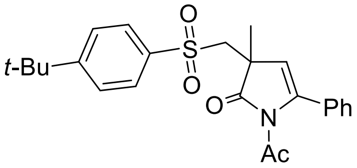

t-Bu
O
S
O
O
N
Ac
Ph

4c

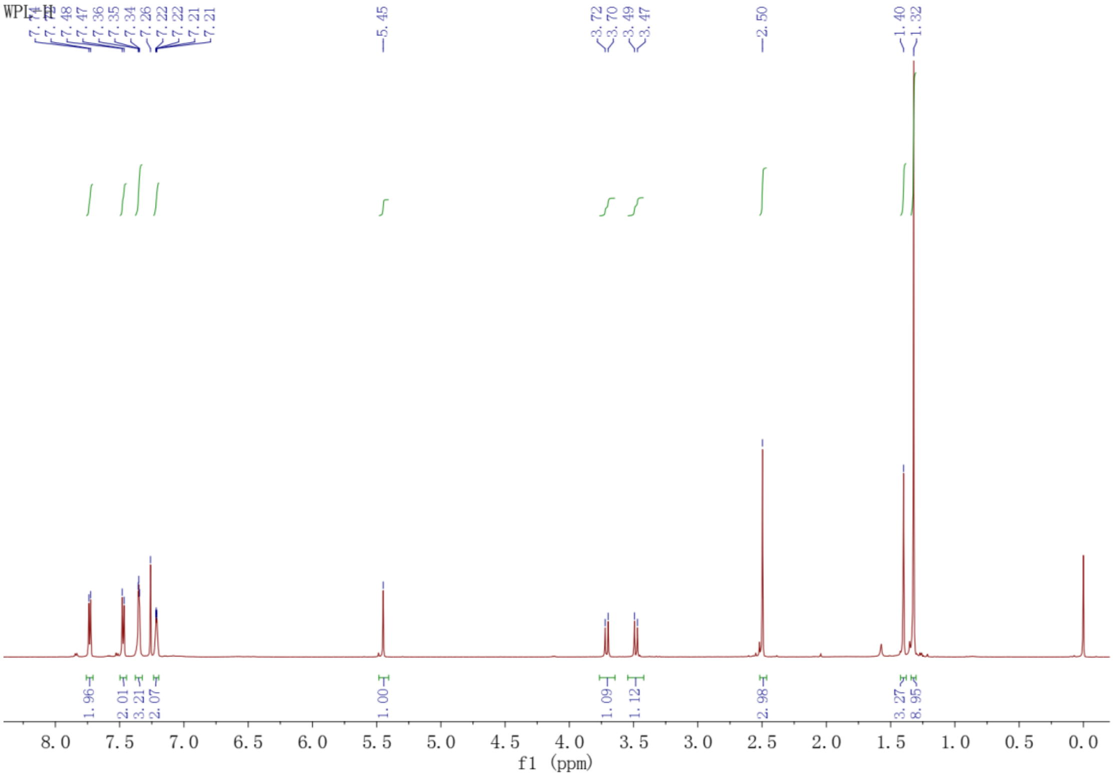

WPL-H
7.48
7.47
7.36
7.35
7.34
7.26
7.22
7.22
7.21
7.21
5.45
3.72
3.70
3.49
3.47
2.50
1.40
1.32
1.96
2.01
3.21
2.07
1.00
1.09
1.12
2.98
3.27
8.95
8.0 7.5 7.0 6.5 6.0 5.5 5.0 4.5 4.0 3.5 3.0 2.5 2.0 1.5 1.0 0.5 0.0
f1 (ppm)

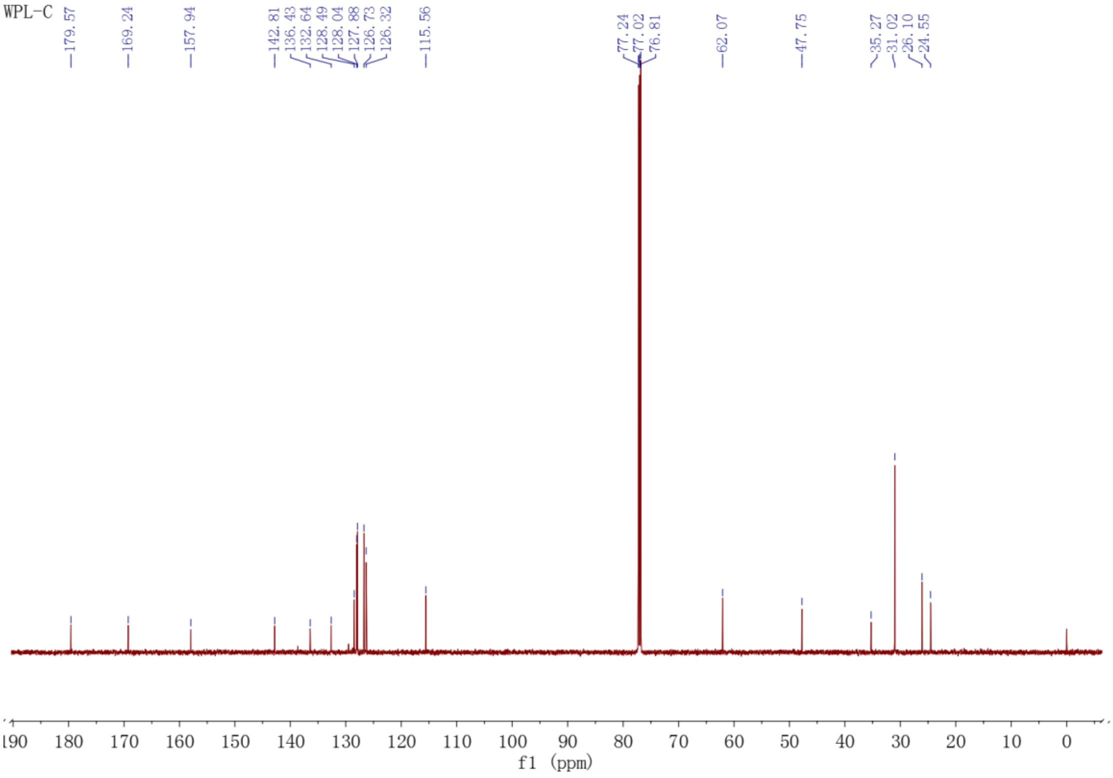

WPL-C
179.57
169.24
157.94
142.81
136.43
132.64
128.49
128.04
127.88
126.73
126.32
115.56
77.24
77.02
76.81
62.07
47.75
35.27
31.02
26.10
24.55
190 180 170 160 150 140 130 120 110 100 90 80 70 60 50 40 30 20 10 0
f1 (ppm)

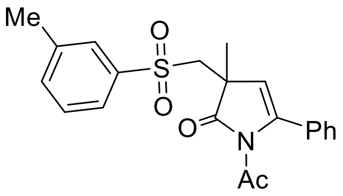
Me
O
S
O
O
N
Ac
Ph

4d

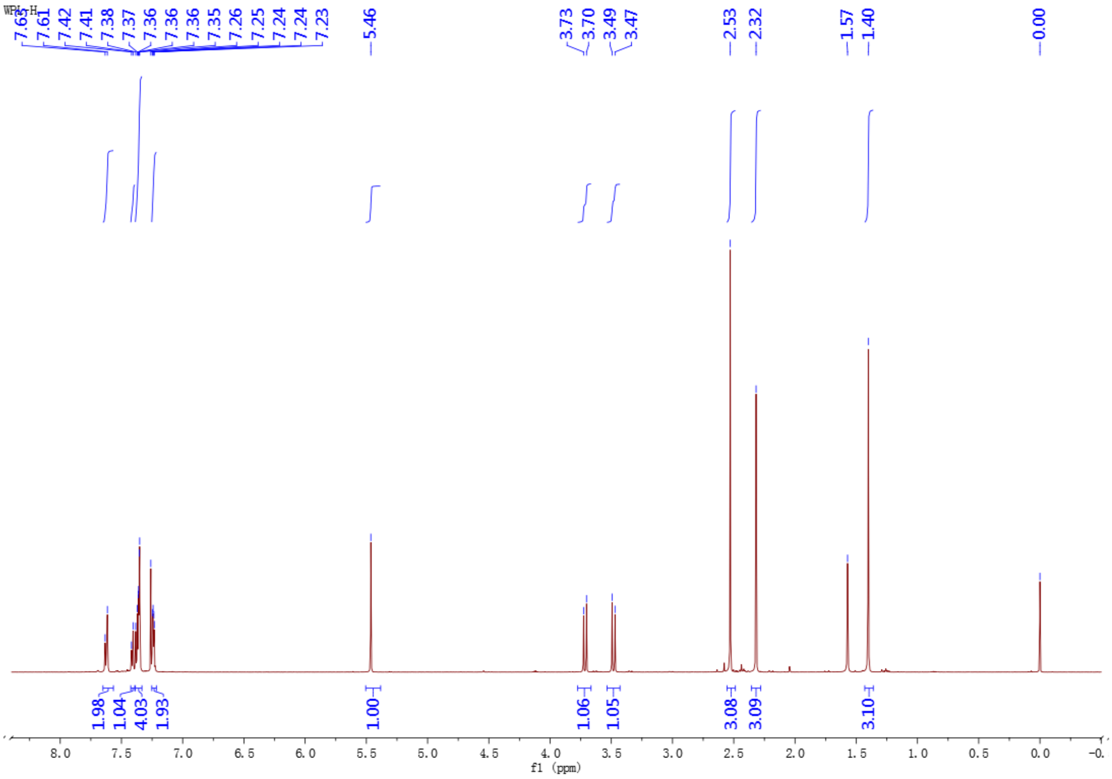
WPL-H
7.61
7.42
7.41
7.38
7.37
7.36
7.36
7.35
7.26
7.25
7.24
7.24
7.23
5.46
3.73
3.70
3.49
3.47
2.53
2.32
1.57
1.40
0.00
1.98
1.04
4.03
1.93
1.00
1.06
1.05
3.08
3.09
3.10
8.0 7.5 7.0 6.5 6.0 5.5 5.0 4.5 4.0 3.5 3.0 2.5 2.0 1.5 1.0 0.5 0.0
f1 (ppm)

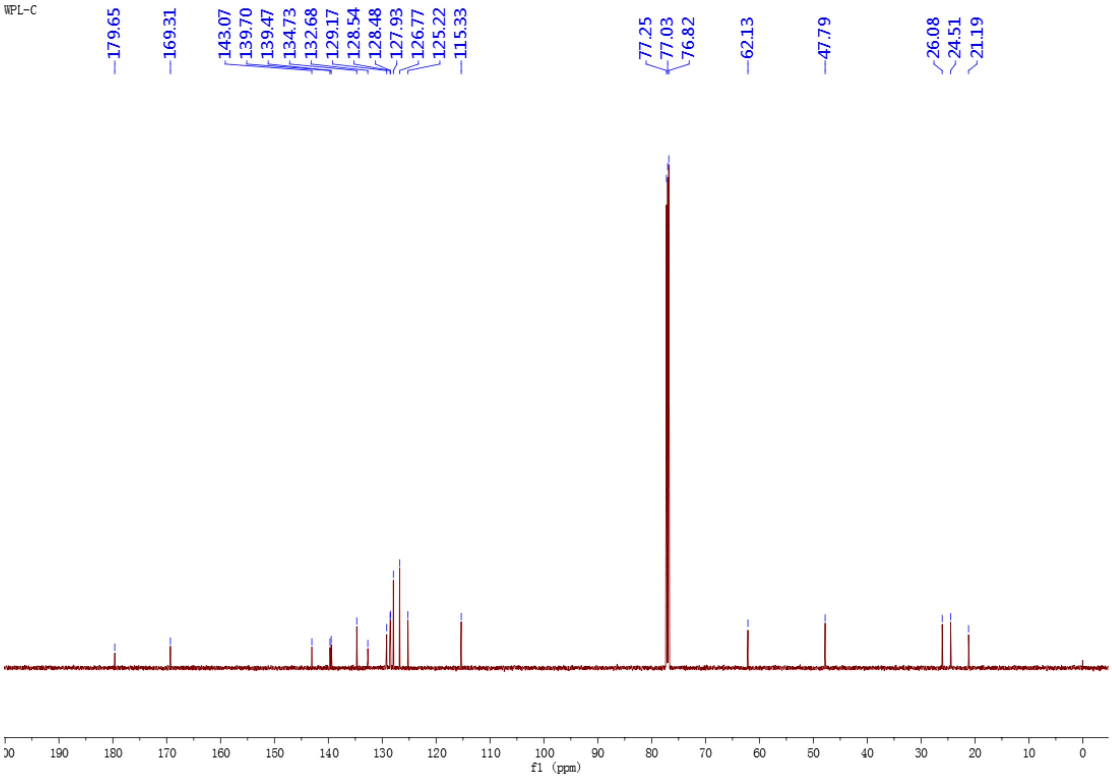
WPL-C
179.65
169.31
143.07
139.70
139.47
134.73
132.68
129.17
128.54
128.48
127.93
126.77
125.22
115.33
77.25
77.03
76.82
62.13
47.79
26.08
24.51
21.19
190 180 170 160 150 140 130 120 110 100 90 80 70 60 50 40 30 20 10 0
f1 (ppm)

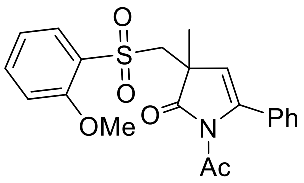
O
S
O
OMe
O
N
Ac
Ph

4e

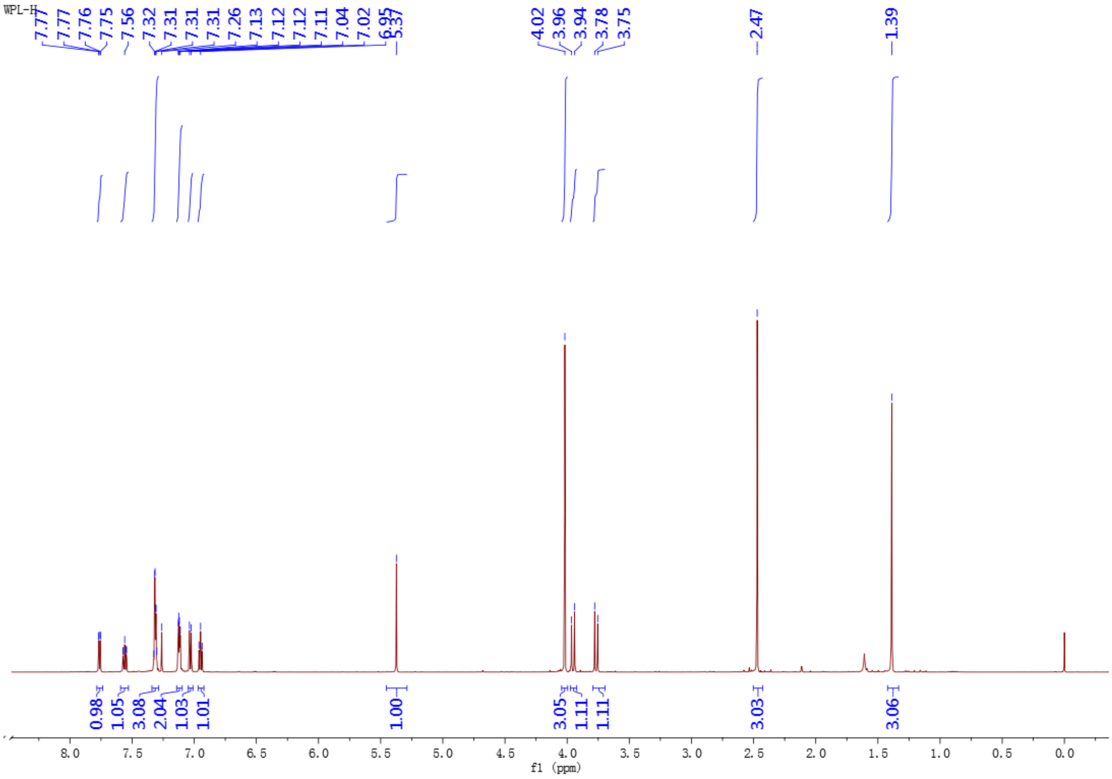
WPL-H
7.77
7.77
7.76
7.75
7.56
7.32
7.31
7.31
7.31
7.26
7.13
7.12
7.12
7.11
7.04
7.02
6.95
5.37
4.02
3.96
3.94
3.78
3.75
2.47
1.39
0.98
1.05
3.08
2.04
1.03
1.01
1.00
3.05
1.11
1.11
3.03
3.06
8.0 7.5 7.0 6.5 6.0 5.5 5.0 4.5 4.0 3.5 3.0 2.5 2.0 1.5 1.0 0.5 0.0
f1 (ppm)

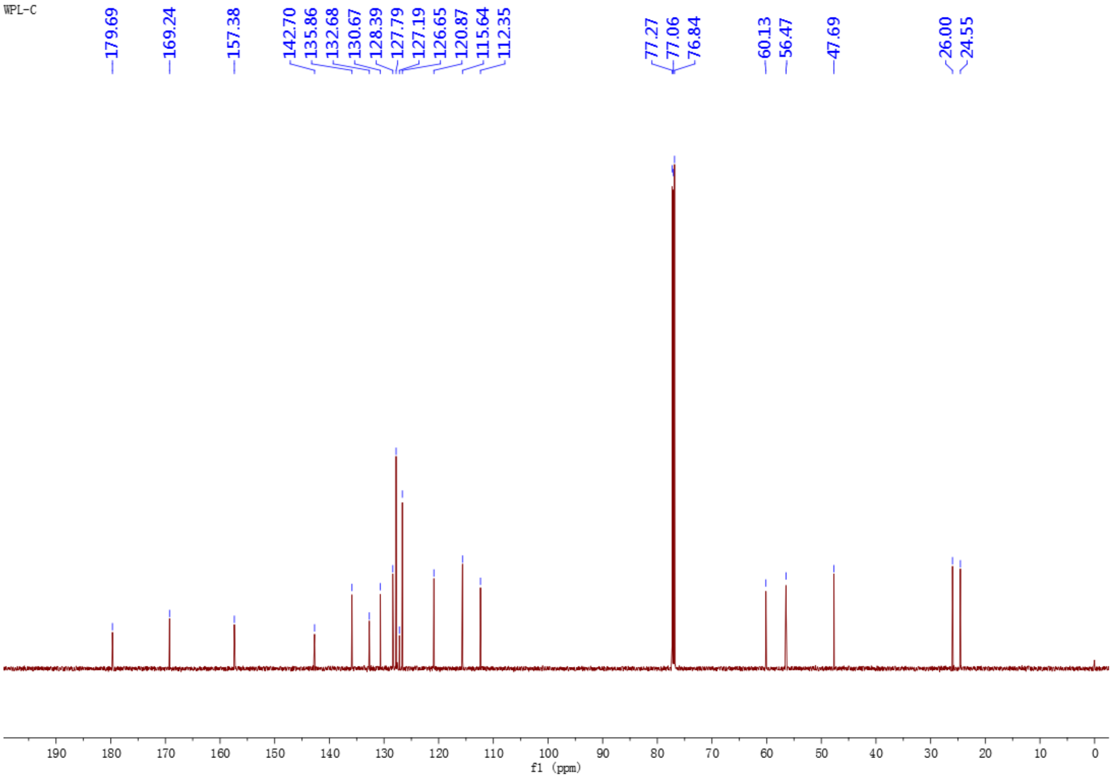
WPL-C
179.69
169.24
157.38
142.70
135.86
132.68
130.67
128.39
127.79
127.19
126.65
120.87
115.64
112.35
77.27
77.06
76.84
60.13
56.47
47.69
26.00
24.55
190 180 170 160 150 140 130 120 110 100 90 80 70 60 50 40 30 20 10 0
f1 (ppm)

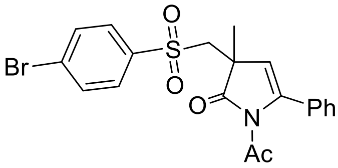
Br
O
S
O
O
N
Ph
Ac

4f

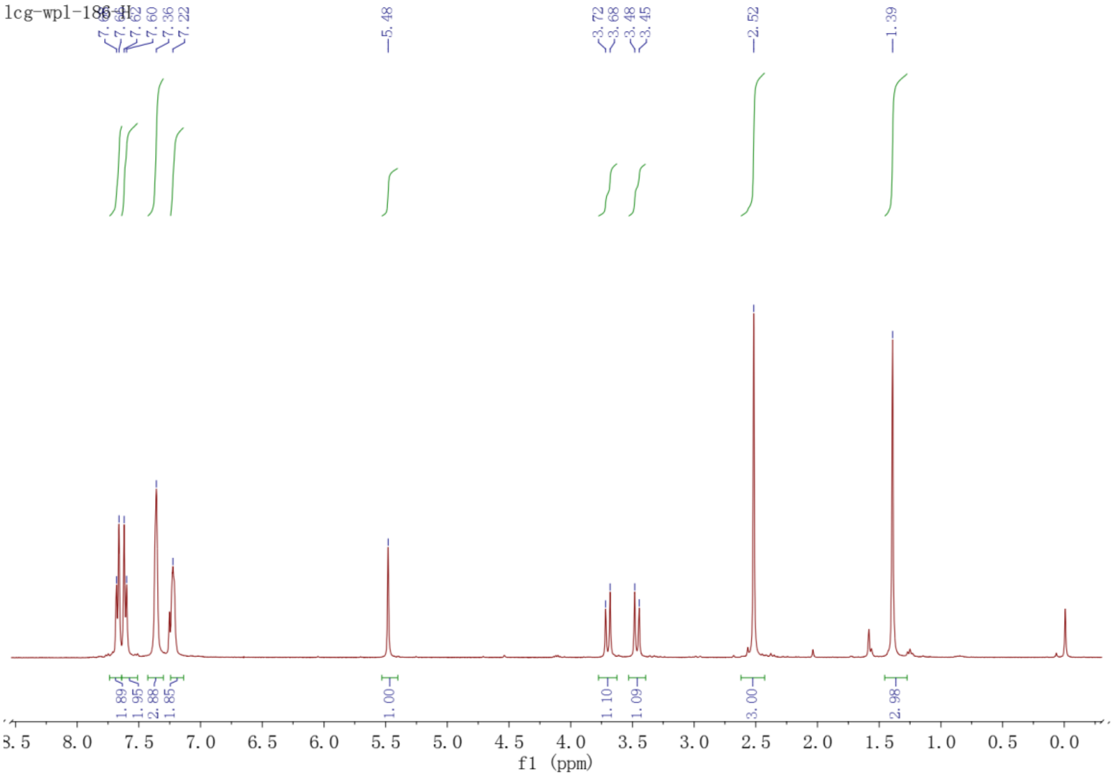
lcg-wpl-186-H
7.62
7.60
7.36
7.22
5.48
3.72
3.68
3.48
3.45
2.52
1.39
1.89
1.95
2.88
1.85
1.00
1.10
1.09
3.00
2.98
8.5 8.0 7.5 7.0 6.5 6.0 5.5 5.0 4.5 4.0 3.5 3.0 2.5 2.0 1.5 1.0 0.5 0.0
f1 (ppm)

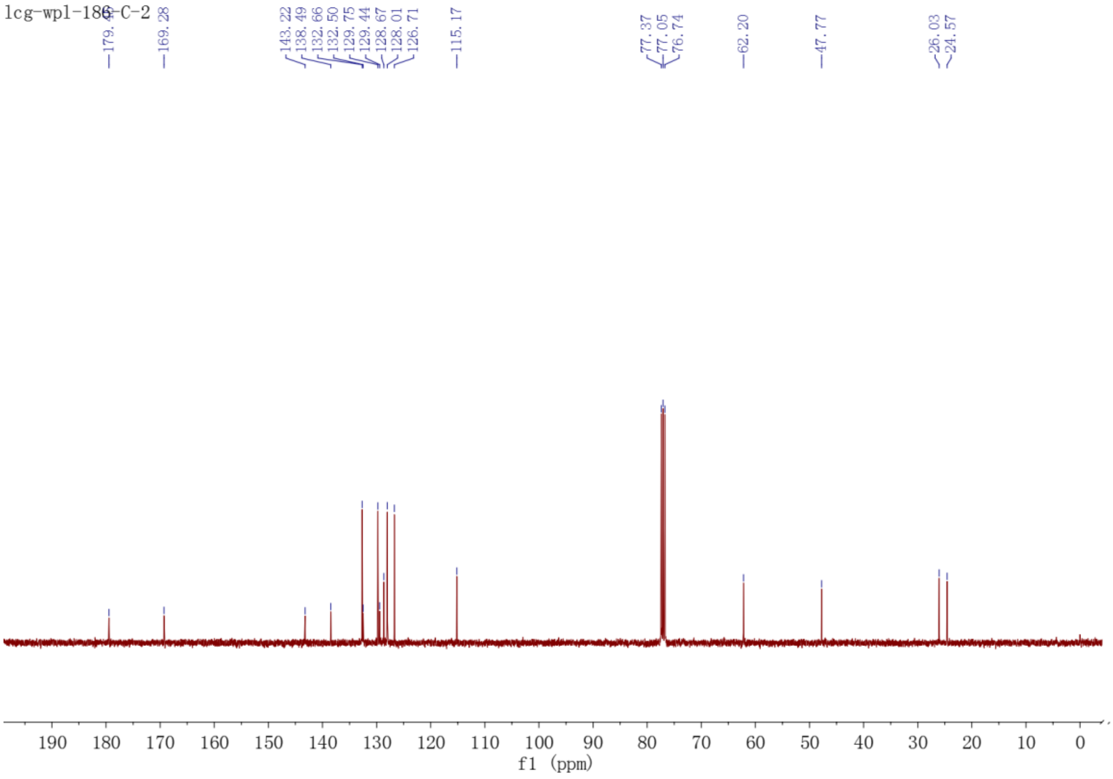
lcg-wpl-186-C-2
179.
169.28
143.22
138.49
132.66
132.50
129.75
129.44
128.67
128.01
126.71
115.17
77.37
77.05
76.74
62.20
47.77
26.03
24.57
190 180 170 160 150 140 130 120 110 100 90 80 70 60 50 40 30 20 10 0
f1 (ppm)

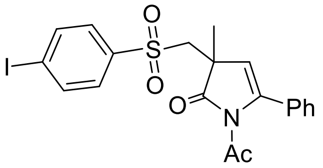
I
O
S
O
O
N
Ph
Ac

4g

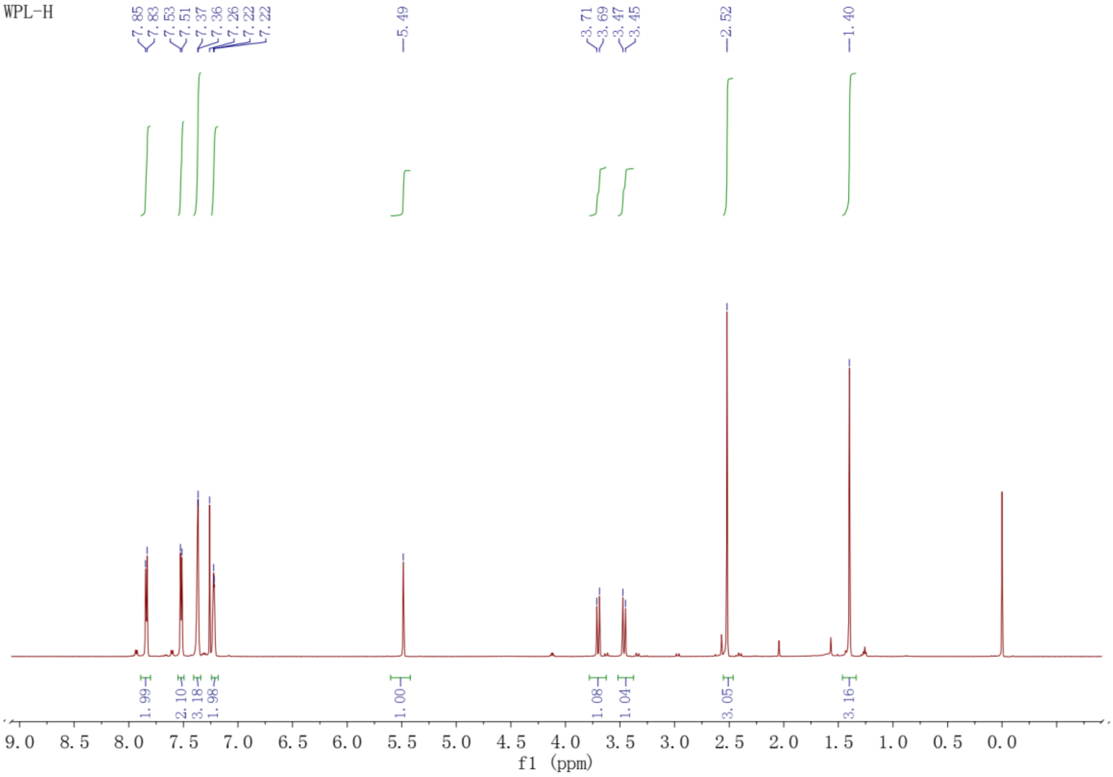
WPL-H
7.85
7.83
7.53
7.51
7.37
7.36
7.26
7.22
7.22
5.49
3.71
3.69
3.47
3.45
2.52
1.40
1.99
2.10
3.18
1.98
1.00
1.08
1.04
3.05
3.16
9.0 8.5 8.0 7.5 7.0 6.5 6.0 5.5 5.0 4.5 4.0 3.5 3.0 2.5 2.0 1.5 1.0 0.5 0.0
f1 (ppm)

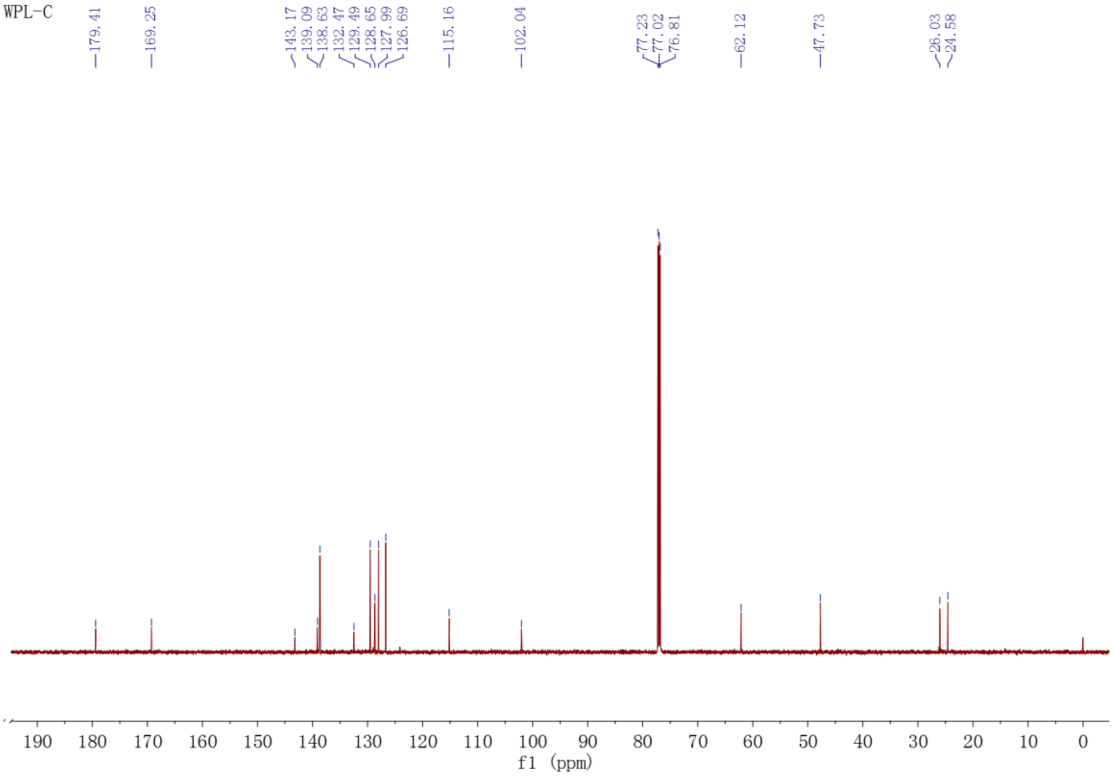
WPL-C
179.41
169.25
143.17
139.09
138.63
132.47
129.49
128.65
127.99
126.69
115.16
102.04
77.23
77.02
76.81
62.12
47.73
26.03
24.58
190 180 170 160 150 140 130 120 110 100 90 80 70 60 50 40 30 20 10 0
f1 (ppm)

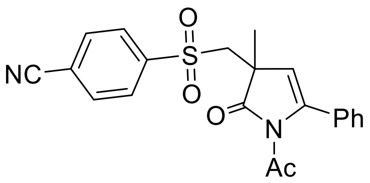
NC
O
S
O
O
N
Ph
Ac

4h

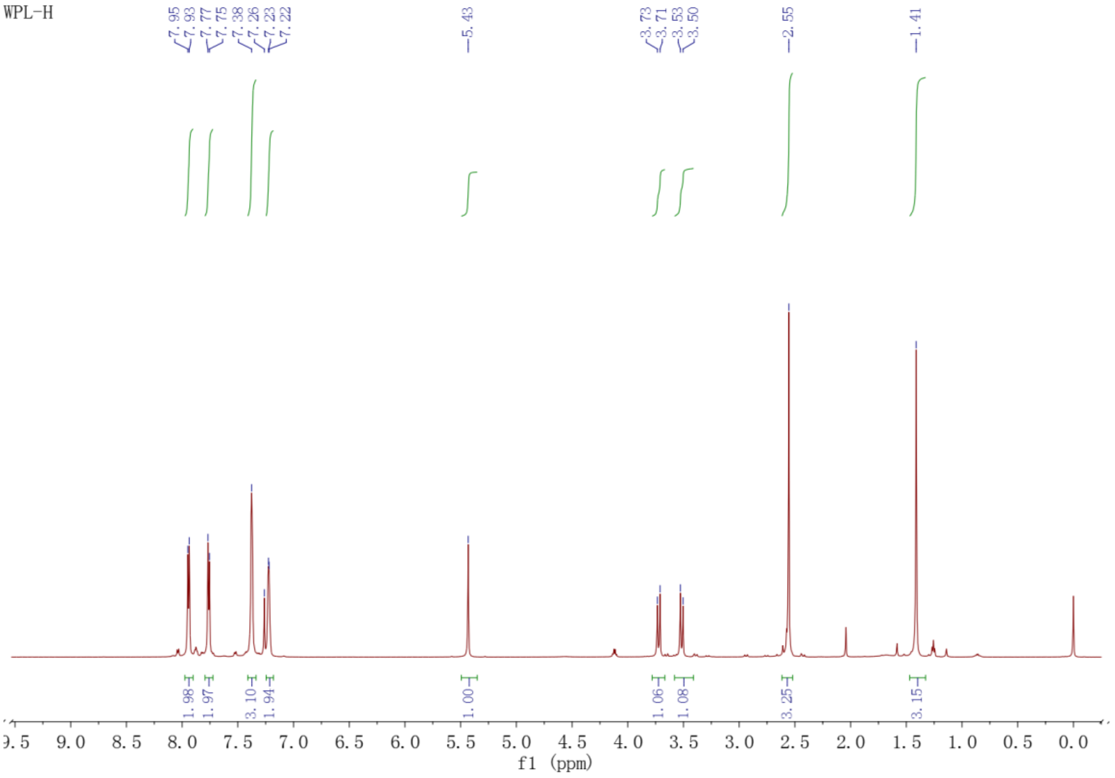
WPL-H
7.95
7.93
7.77
7.75
7.38
7.26
7.23
7.22
5.43
3.73
3.71
3.53
3.50
2.55
1.41
1.98
1.97
3.10
1.94
1.00
1.06
1.08
3.25
3.15
9.5 9.0 8.5 8.0 7.5 7.0 6.5 6.0 5.5 5.0 4.5 4.0 3.5 3.0 2.5 2.0 1.5 1.0 0.5 0.0
f1 (ppm)

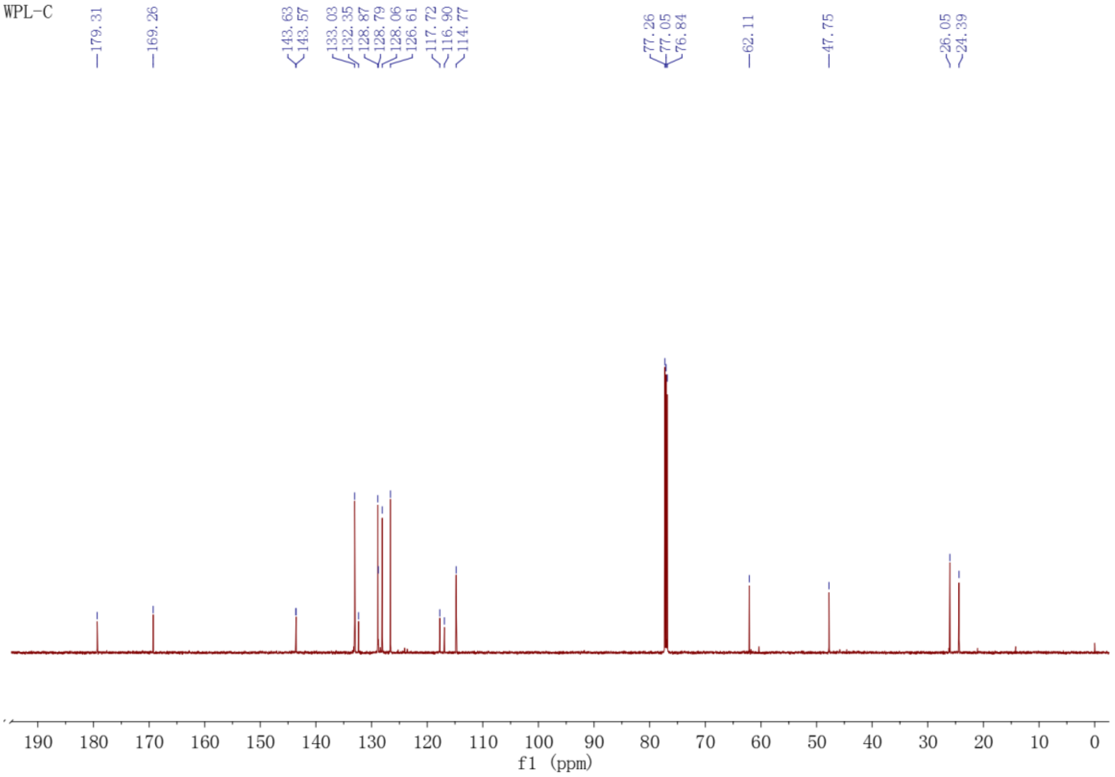
WPL-C
179.31
169.26
143.63
143.57
133.03
132.35
128.87
128.79
128.06
126.61
117.72
116.90
114.77
77.26
77.05
76.84
62.11
47.75
26.05
24.39
190 180 170 160 150 140 130 120 110 100 90 80 70 60 50 40 30 20 10 0
f1 (ppm)

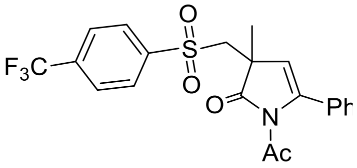

F3C
O
S
O
O
N
Ac
Ph

4i

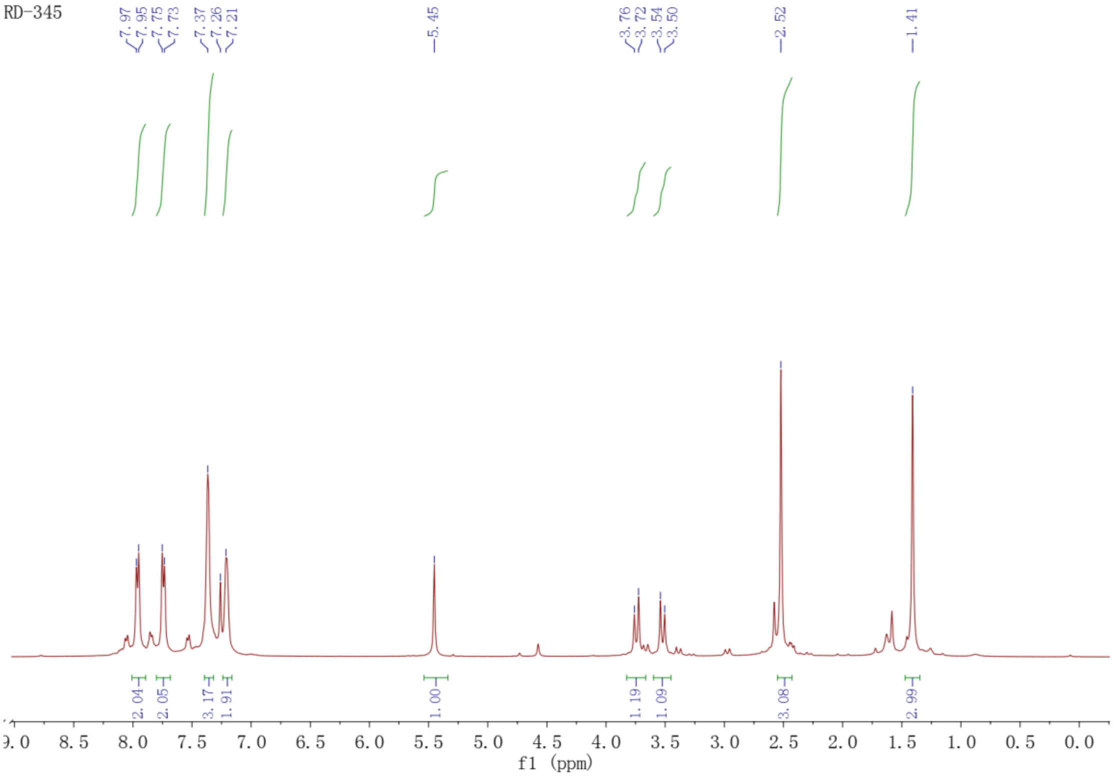

RD-345
7.97
7.95
7.75
7.73
7.37
7.26
7.21
5.45
3.76
3.72
3.54
3.50
2.52
1.41
2.04
2.05
3.17
1.91
1.00
1.19
1.09
3.08
2.99
f1 (ppm)

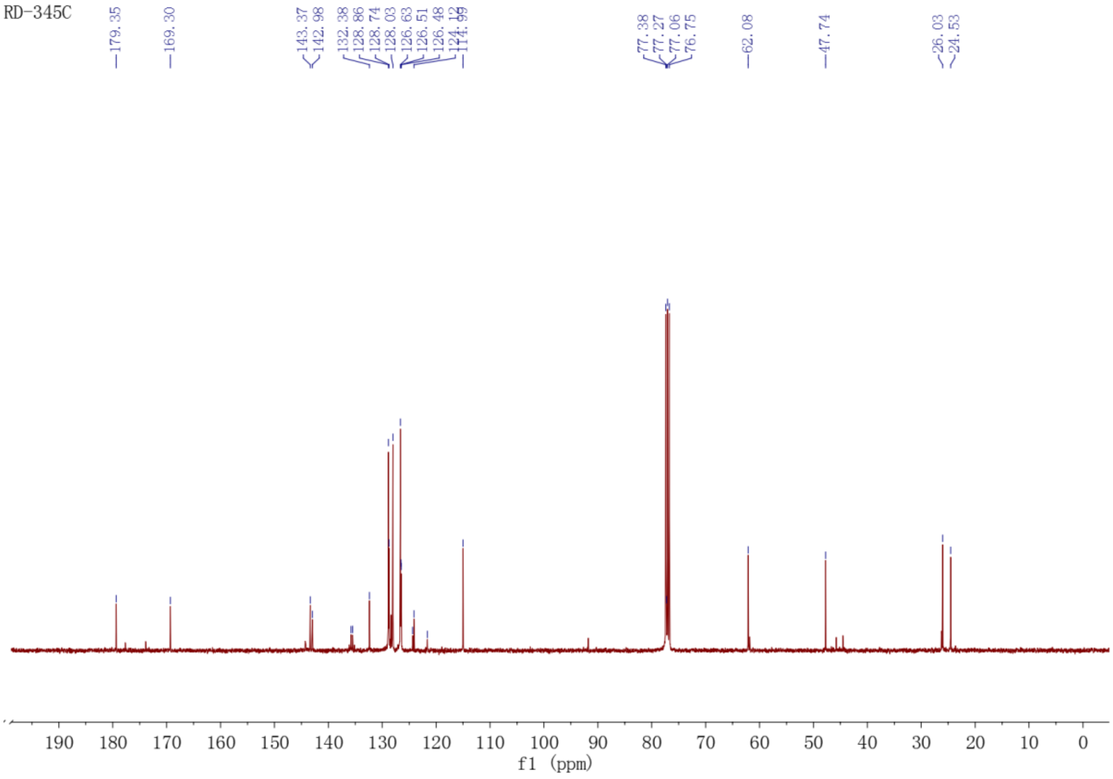

RD-345C
179.35
169.30
143.37
142.98
132.38
128.86
128.74
128.03
126.63
126.51
126.48
124.12
114.99
77.38
77.27
77.06
76.75
62.08
47.74
26.03
24.53
f1 (ppm)

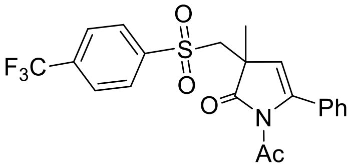

4i

RD-345F

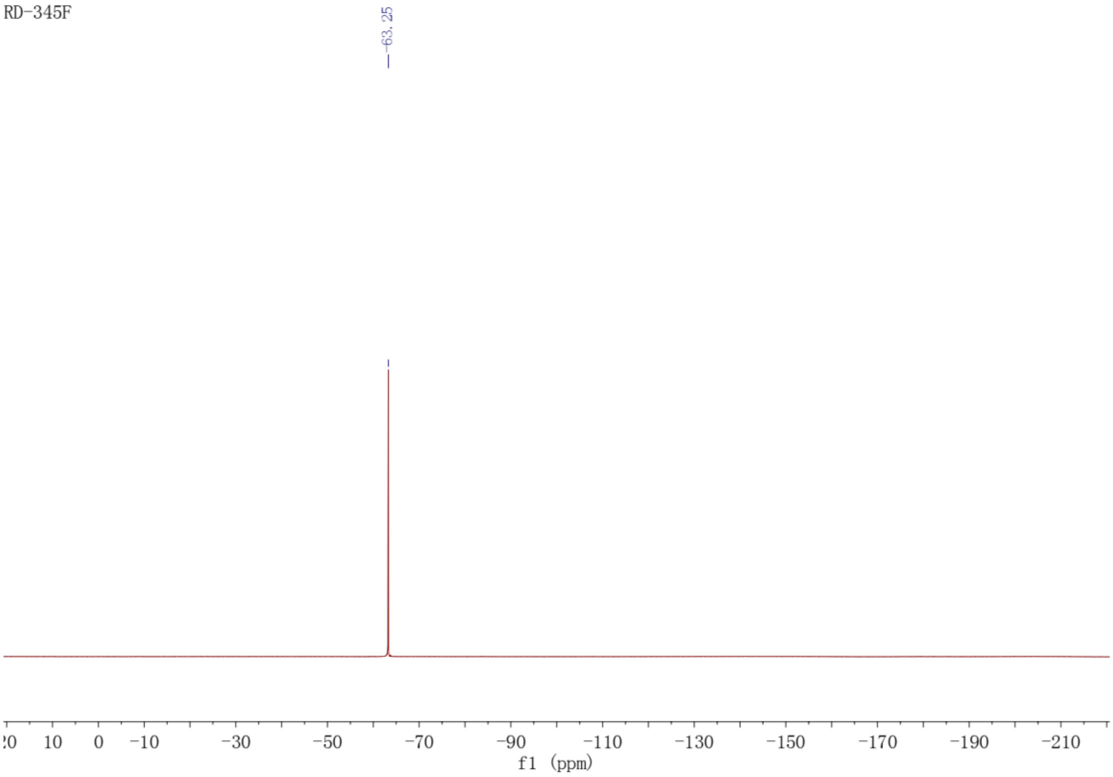

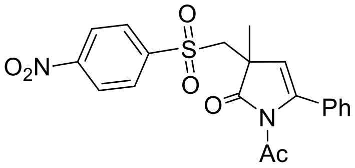

O2N
O
S
O
O
N
Ph
Ac

4j

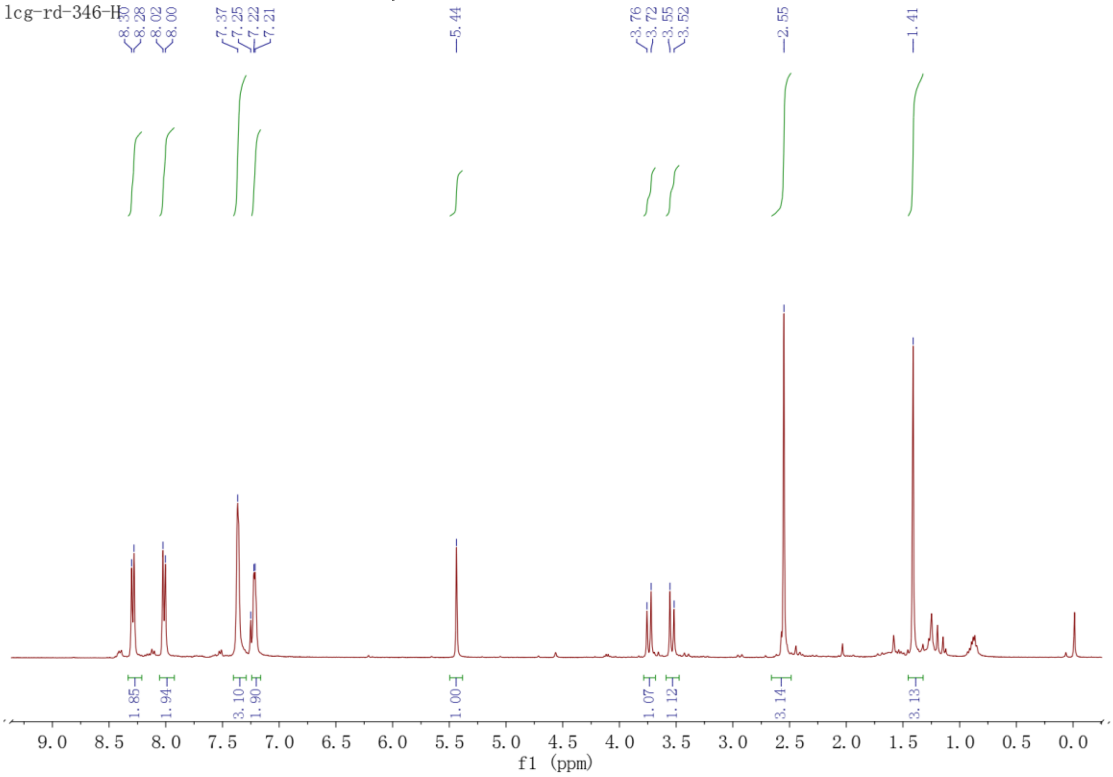

lcg-rd-346-H
8.30
8.28
8.02
8.00
7.37
7.25
7.22
7.21
5.44
3.76
3.72
3.55
3.52
2.55
1.41
1.85
1.94
3.10
1.90
1.00
1.07
1.12
3.14
3.13
9.0 8.5 8.0 7.5 7.0 6.5 6.0 5.5 5.0 4.5 4.0 3.5 3.0 2.5 2.0 1.5 1.0 0.5 0.0
f1 (ppm)

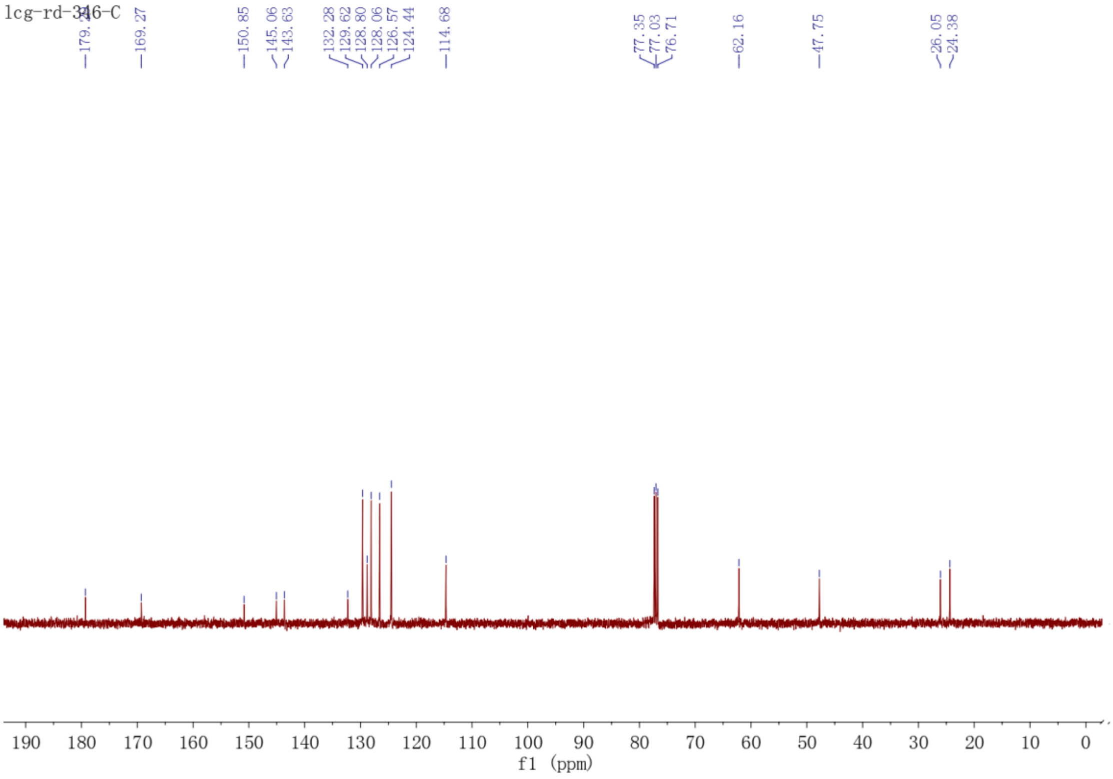

lcg-rd-346-C
179.
169.27
150.85
145.06
143.63
132.28
129.62
128.80
128.06
126.57
124.44
114.68
77.35
77.03
76.71
62.16
47.75
26.05
24.38
190 180 170 160 150 140 130 120 110 100 90 80 70 60 50 40 30 20 10 0
f1 (ppm)

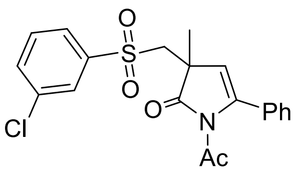
Cl
O
S
O
O
N
Ac
Ph

4k

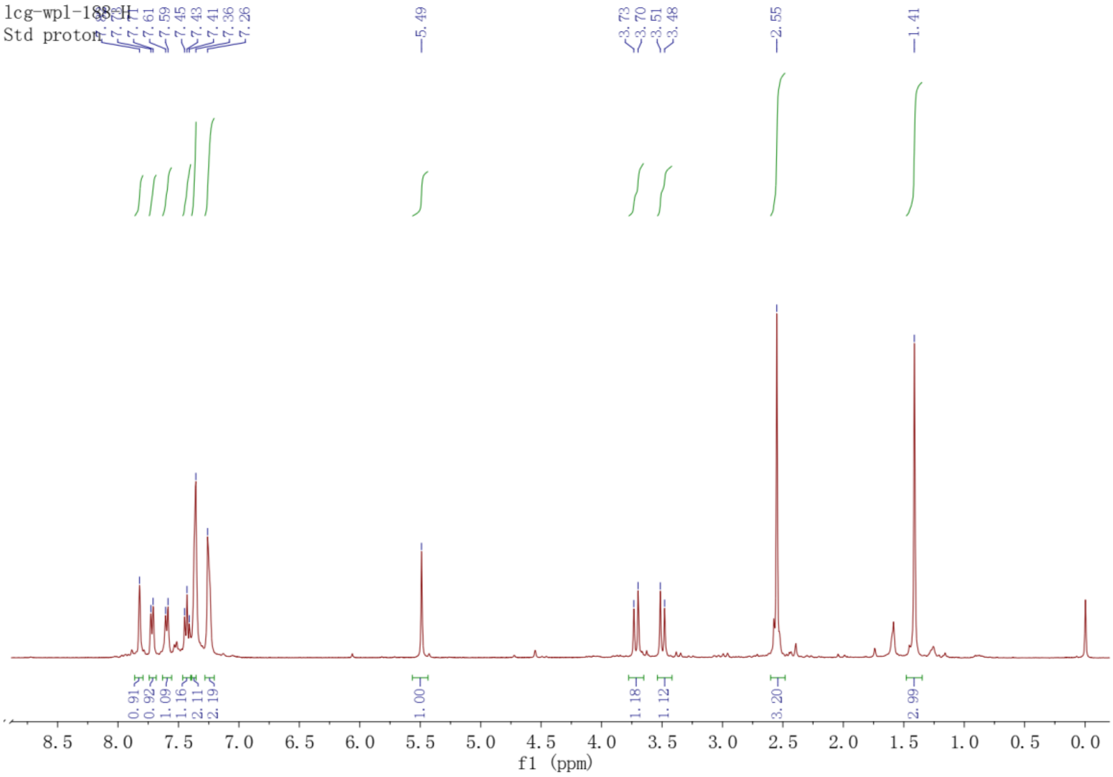
lcg-wpl-188-H
Std proton
7.61
7.59
7.45
7.43
7.41
7.36
7.26
5.49
3.73
3.70
3.51
3.48
2.55
1.41
0.91
0.92
1.09
1.16
2.11
2.19
1.00
1.18
1.12
3.20
2.99
f1 (ppm)

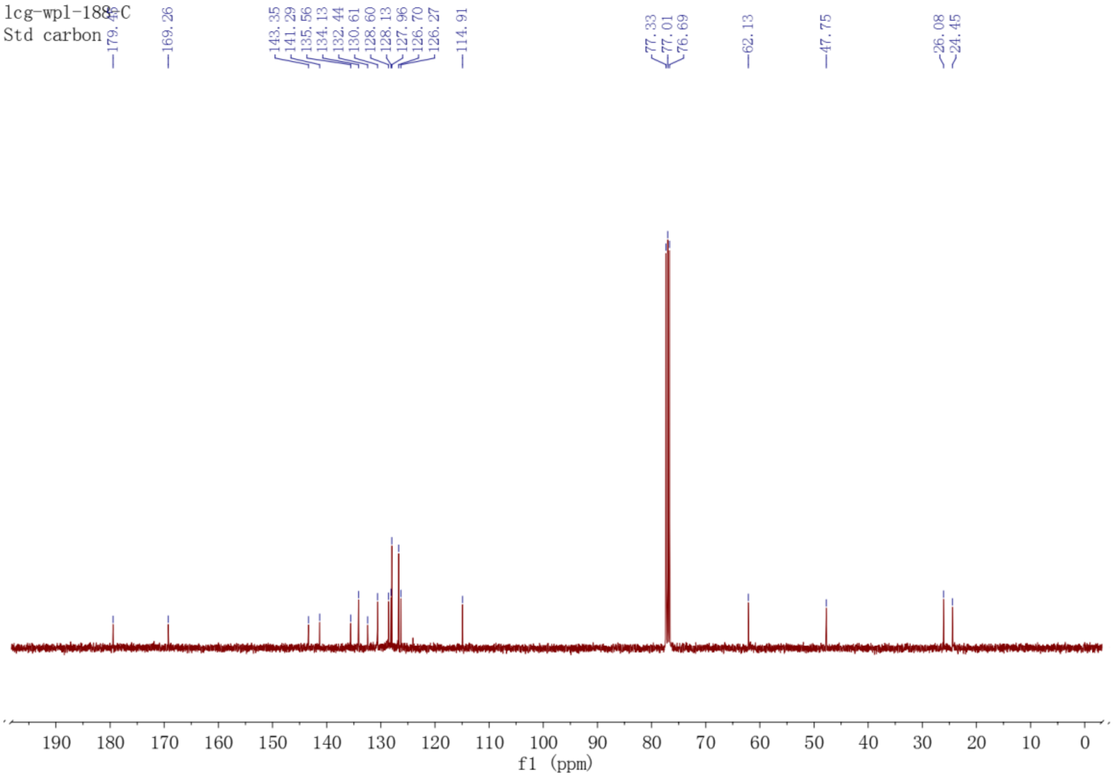
lcg-wpl-188-C
Std carbon
169.26
143.35
141.29
135.56
134.13
132.44
130.61
128.60
128.13
127.96
126.70
126.27
114.91
77.33
77.01
76.69
62.13
47.75
26.08
24.45
f1 (ppm)

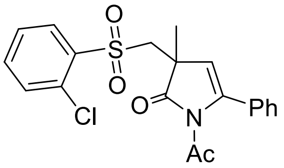
O
S
O
Cl
O
N
Ac
Ph

4l

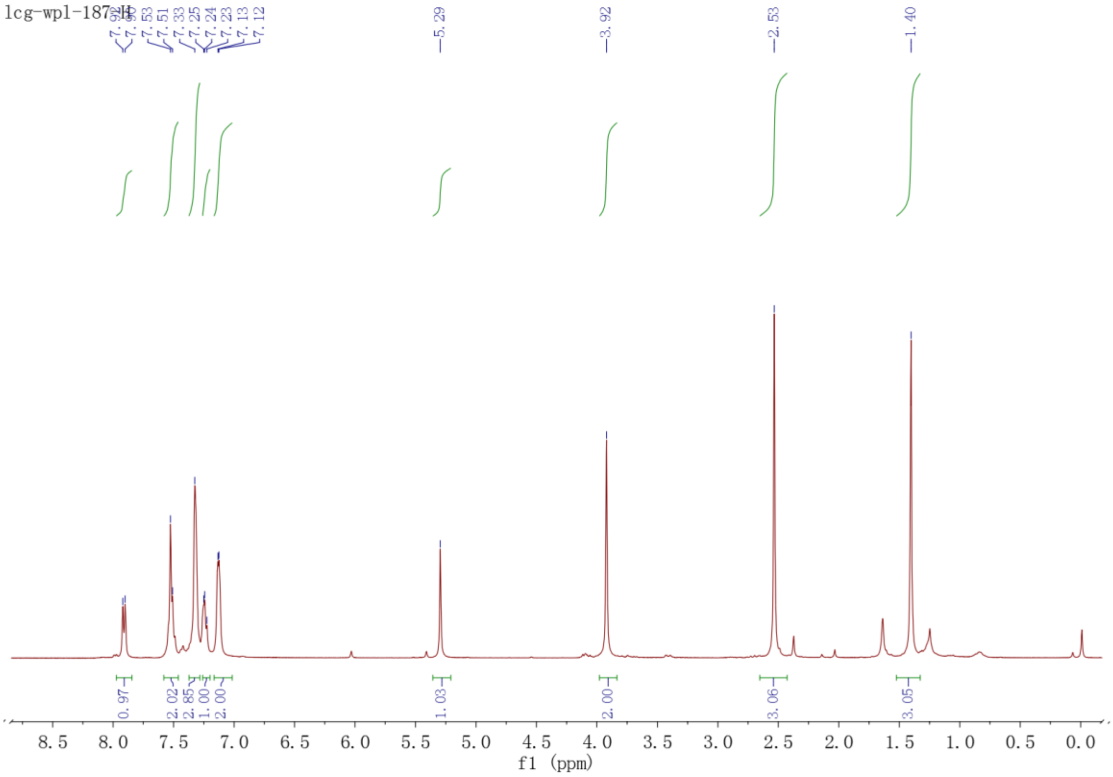
lcg-wpl-187-H
7.53
7.51
7.33
7.25
7.24
7.23
7.13
7.12
5.29
3.92
2.53
1.40
0.97
2.02
2.85
1.00
2.00
1.03
2.00
3.06
3.05
8.5 8.0 7.5 7.0 6.5 6.0 5.5 5.0 4.5 4.0 3.5 3.0 2.5 2.0 1.5 1.0 0.5 0.0
f1 (ppm)

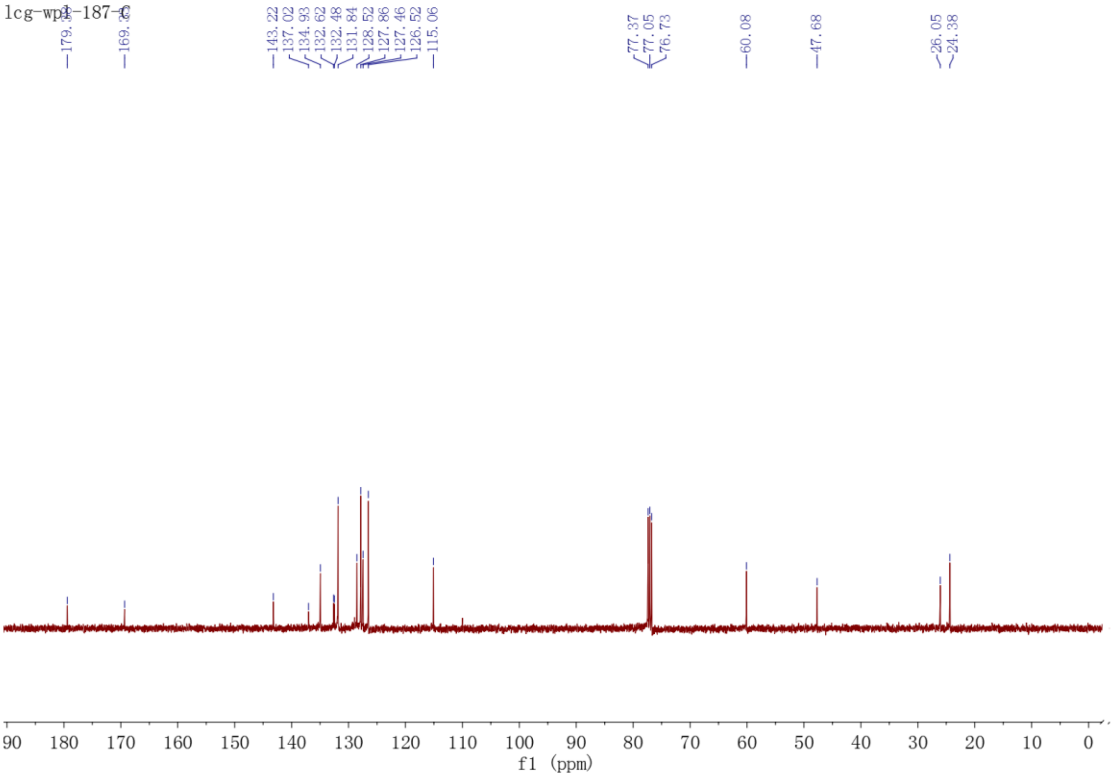
143.22
137.02
134.93
132.62
132.48
131.84
128.52
127.86
127.46
126.52
115.06
77.37
77.05
76.73
60.08
47.68
26.05
24.38
90 180 170 160 150 140 130 120 110 100 90 80 70 60 50 40 30 20 10 0
f1 (ppm)

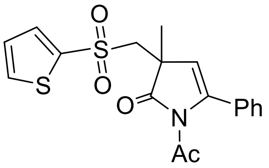

4m

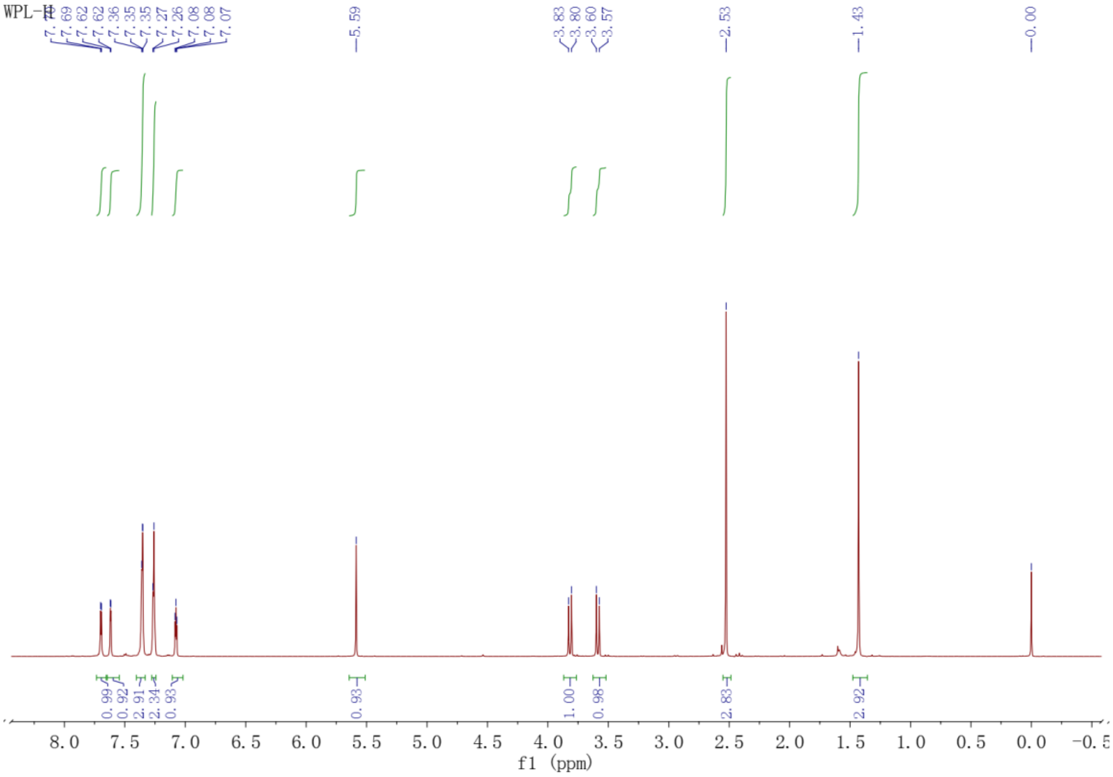
WPL-H
7.69
7.69
7.62
7.62
7.36
7.35
7.35
7.27
7.26
7.08
7.08
7.07
5.59
3.83
3.80
3.60
3.57
2.53
1.43
0.00
0.99
0.92
2.91
2.34
0.93
0.93
1.00
0.98
2.83
2.92
8.0 7.5 7.0 6.5 6.0 5.5 5.0 4.5 4.0 3.5 3.0 2.5 2.0 1.5 1.0 0.5 0.0 -0.5
f1 (ppm)

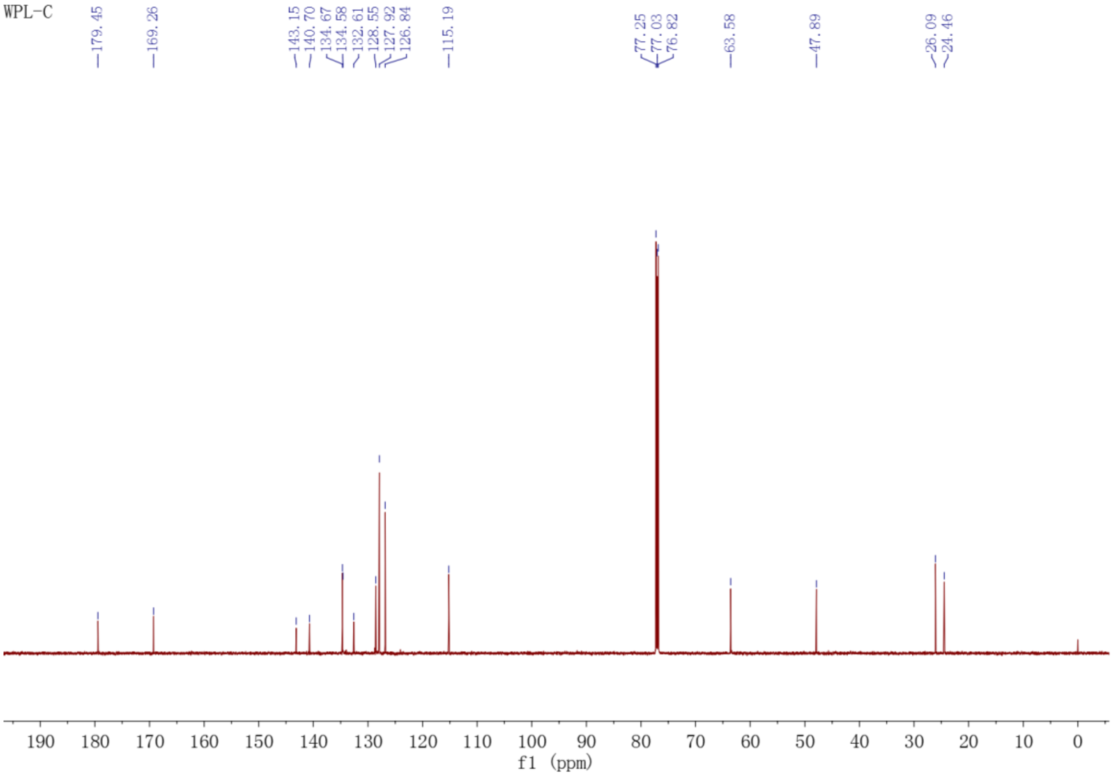
WPL-C
179.45
169.26
143.15
140.70
134.67
134.58
132.61
128.55
127.92
126.84
115.19
77.25
77.03
76.82
63.58
47.89
26.09
24.46
190 180 170 160 150 140 130 120 110 100 90 80 70 60 50 40 30 20 10 0
f1 (ppm)

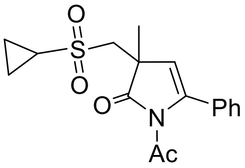
O
S
O
O
N
Ph
Ac

4n

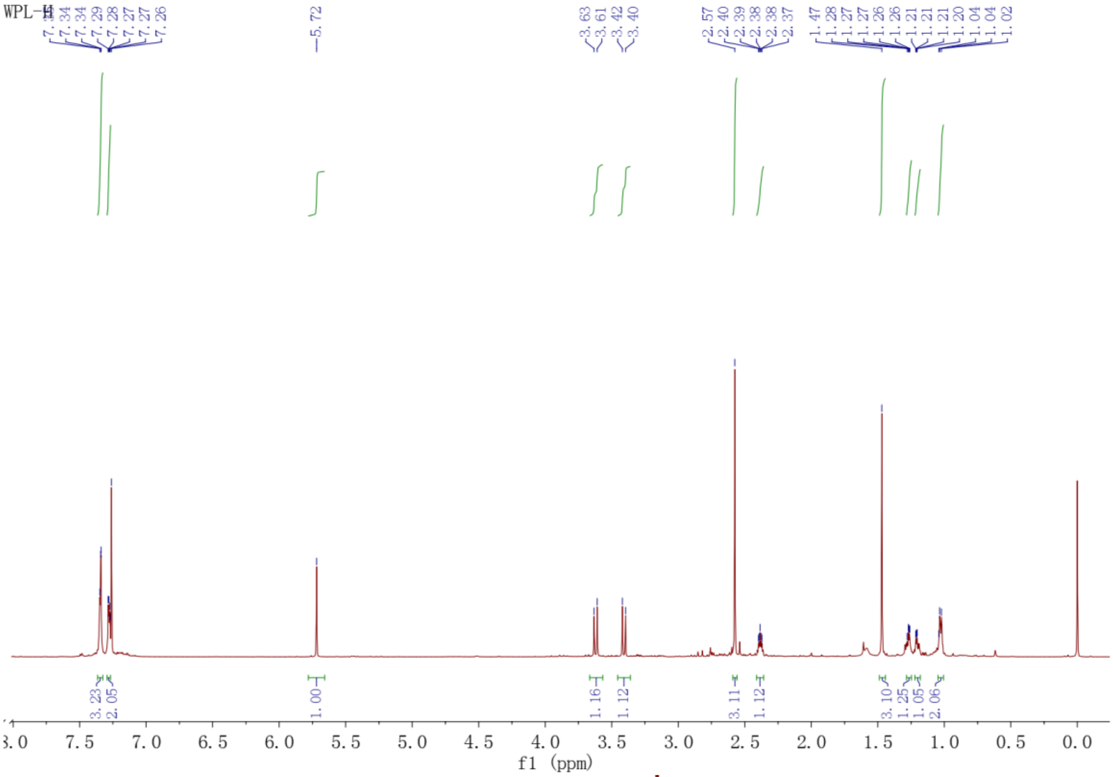
WPL-H
7.34
7.34
7.29
7.28
7.27
7.27
7.26
5.72
3.63
3.61
3.42
3.40
2.57
2.40
2.39
2.38
2.38
2.37
1.47
1.28
1.27
1.27
1.26
1.26
1.21
1.21
1.21
1.20
1.04
1.04
1.02
3.23
2.05
1.00
1.16
1.12
3.11
1.12
3.10
1.25
1.05
2.06
8.0 7.5 7.0 6.5 6.0 5.5 5.0 4.5 4.0 3.5 3.0 2.5 2.0 1.5 1.0 0.5 0.0
f1 (ppm)

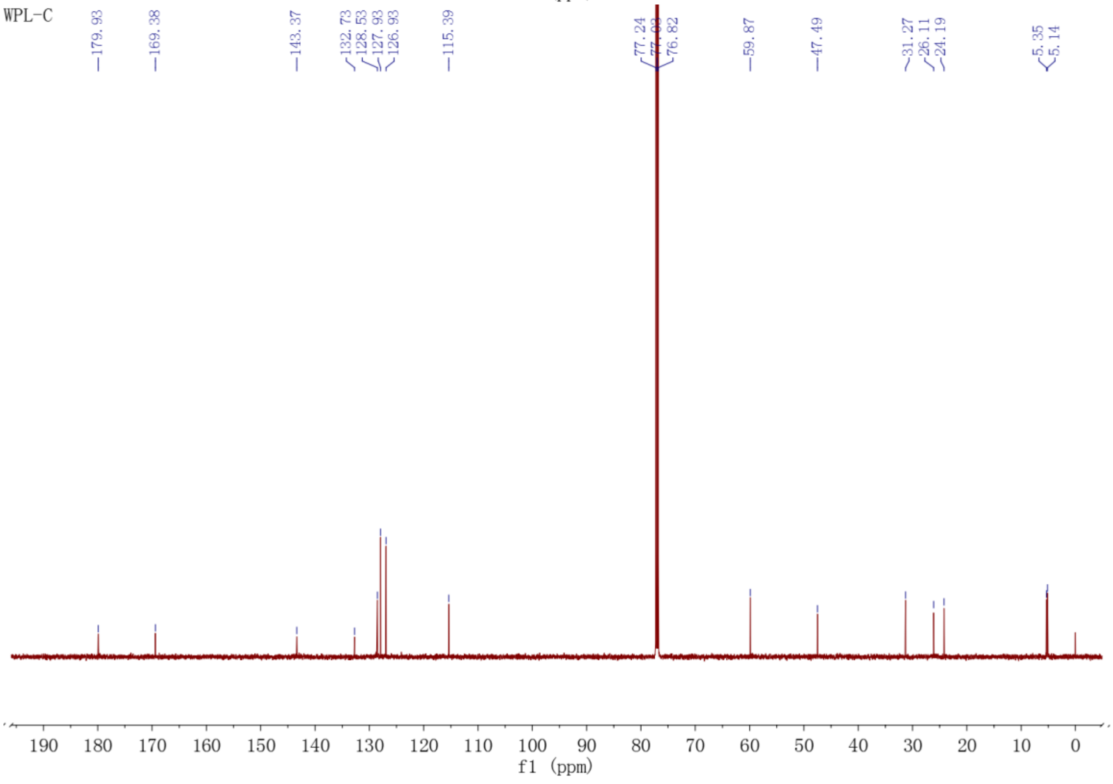
WPL-C
179.93
169.38
143.37
132.73
128.53
127.93
126.93
115.39
77.24
77.03
76.82
59.87
47.49
31.27
26.11
24.19
5.35
5.14
190 180 170 160 150 140 130 120 110 100 90 80 70 60 50 40 30 20 10 0
f1 (ppm)

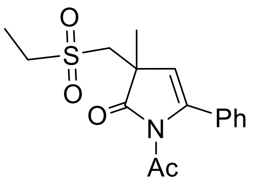

O
S
O
O
N
Ph
Ac

4o

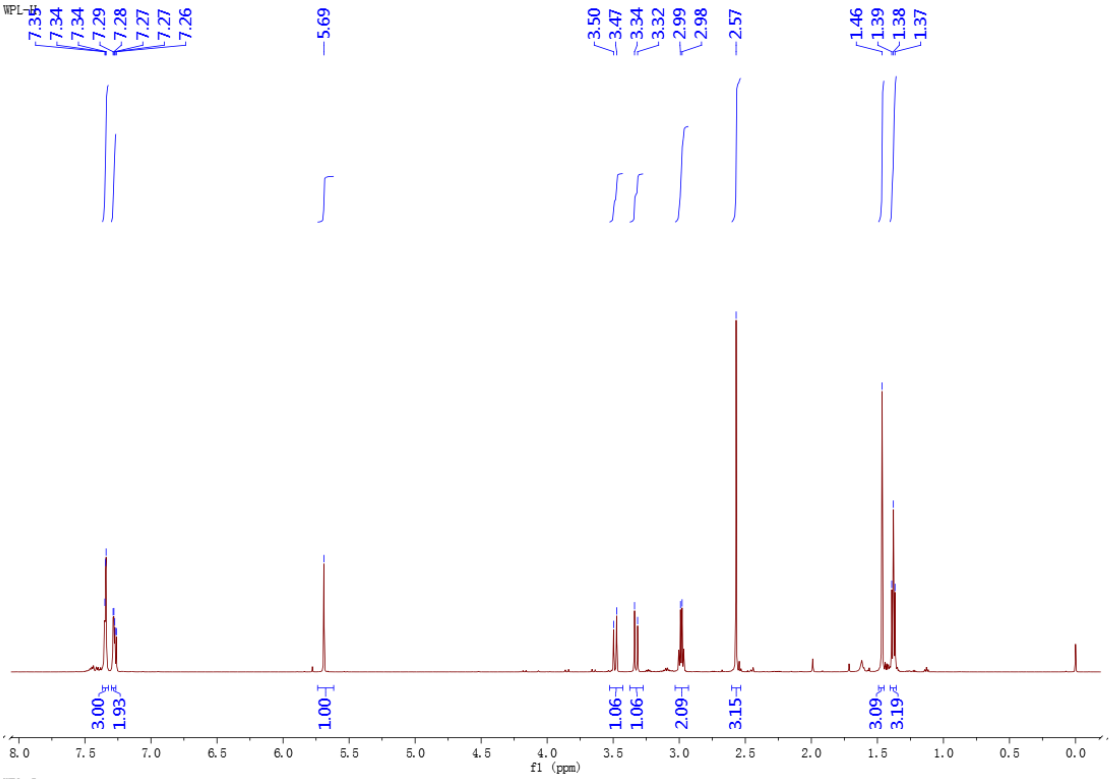

WPL-H
7.35 7.34 7.34 7.29 7.28 7.27 7.27 7.26
5.69
3.50 3.47 3.34 3.32 2.99 2.98
2.57
1.46 1.39 1.38 1.37
3.00 1.93 1.00 1.06 1.06 2.09 3.15 3.09 3.19
8.0 7.5 7.0 6.5 6.0 5.5 5.0 4.5 4.0 3.5 3.0 2.5 2.0 1.5 1.0 0.5 0.0
f1 (ppm)

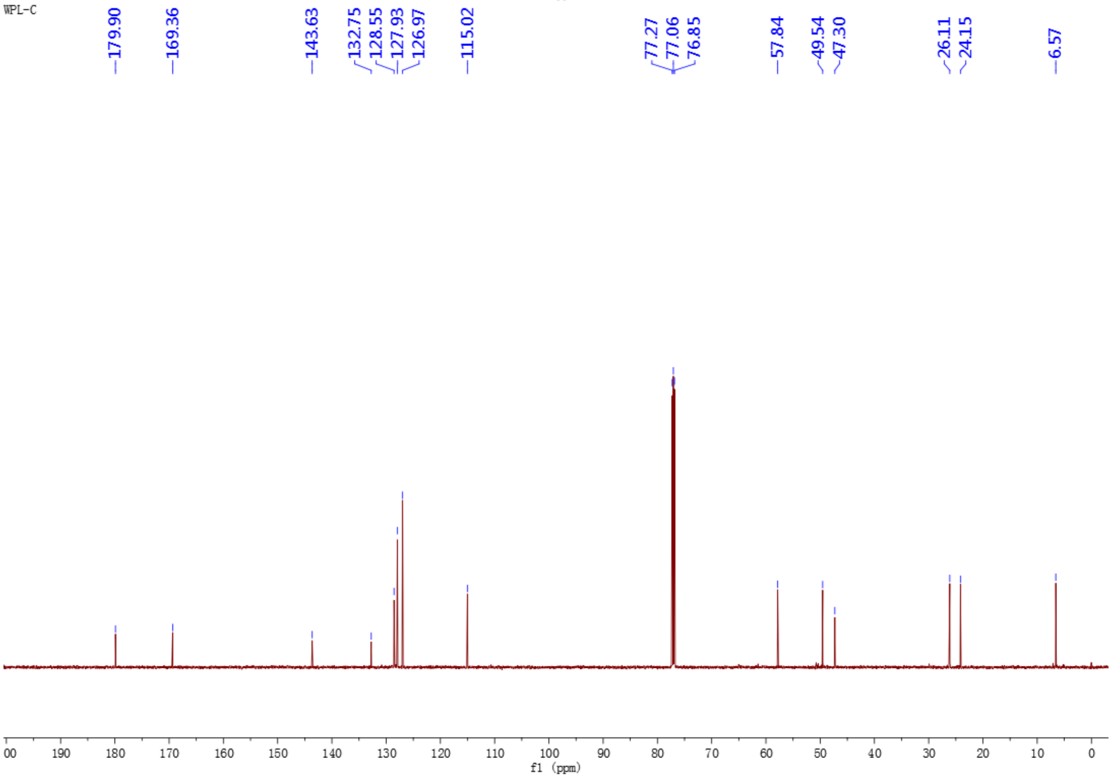

WPL-C
179.90
169.36
143.63
132.75 128.55 127.93 126.97
115.02
77.27 77.06 76.85
57.84
49.54 47.30
26.11 24.15
6.57
00 190 180 170 160 150 140 130 120 110 100 90 80 70 60 50 40 30 20 10 0
f1 (ppm)

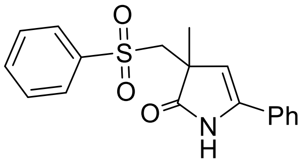
O
S
O
O
N
H
Ph

4aa

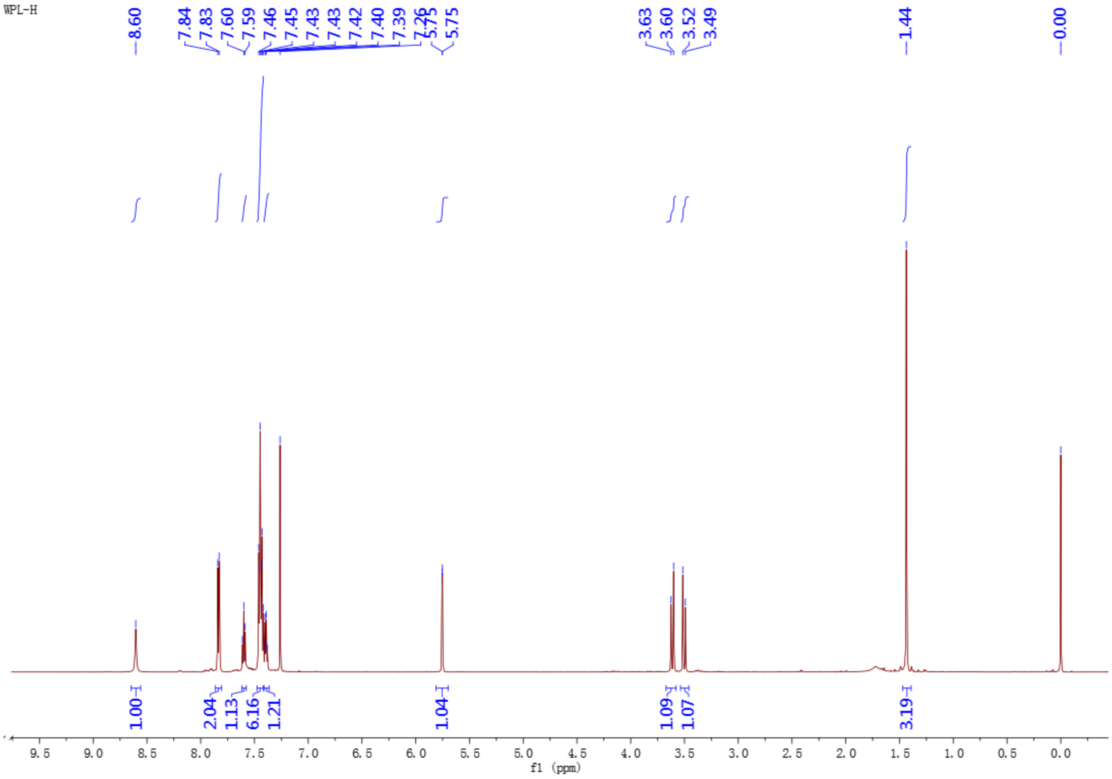
WPL-H
8.60
7.84
7.83
7.60
7.59
7.46
7.45
7.43
7.43
7.42
7.40
7.39
7.26
5.75
5.75
3.63
3.60
3.52
3.49
1.44
0.00
1.00
2.04
1.13
6.16
1.21
1.04
1.09
1.07
3.19
f1 (ppm)

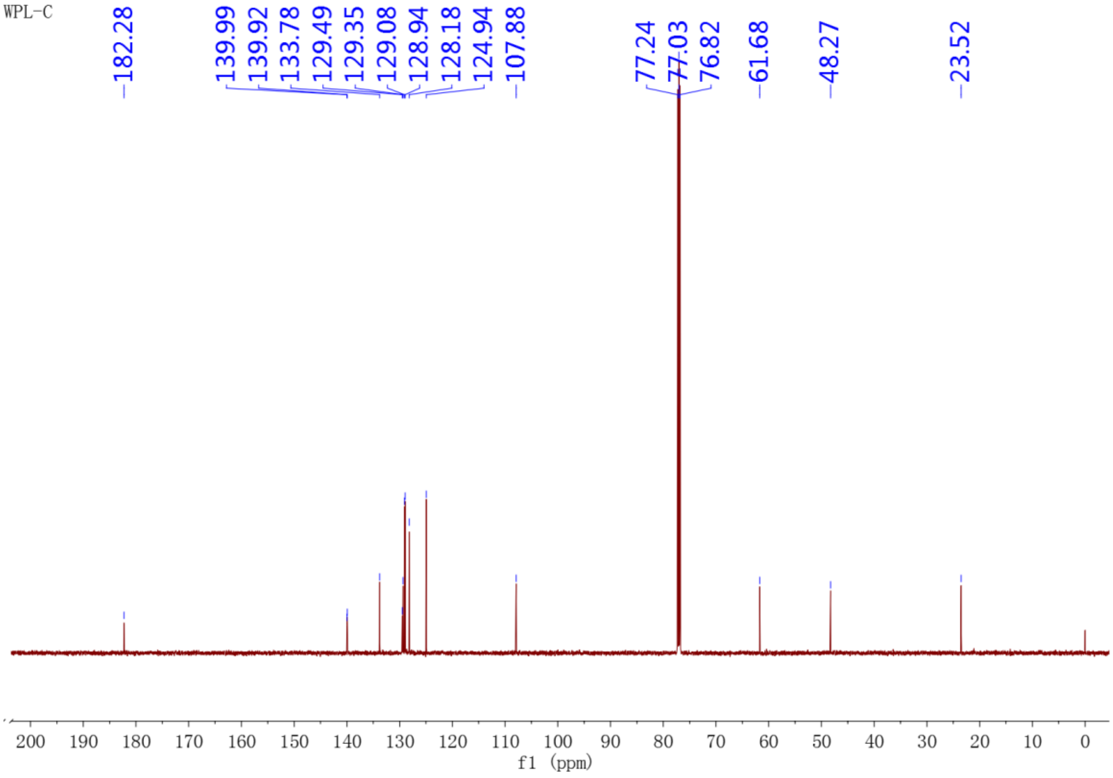
WPL-C
182.28
139.99
139.92
133.78
129.49
129.35
129.08
128.94
128.18
124.94
107.88
77.24
77.03
76.82
61.68
48.27
23.52
f1 (ppm)

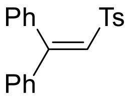
Ph
Ts
Ph

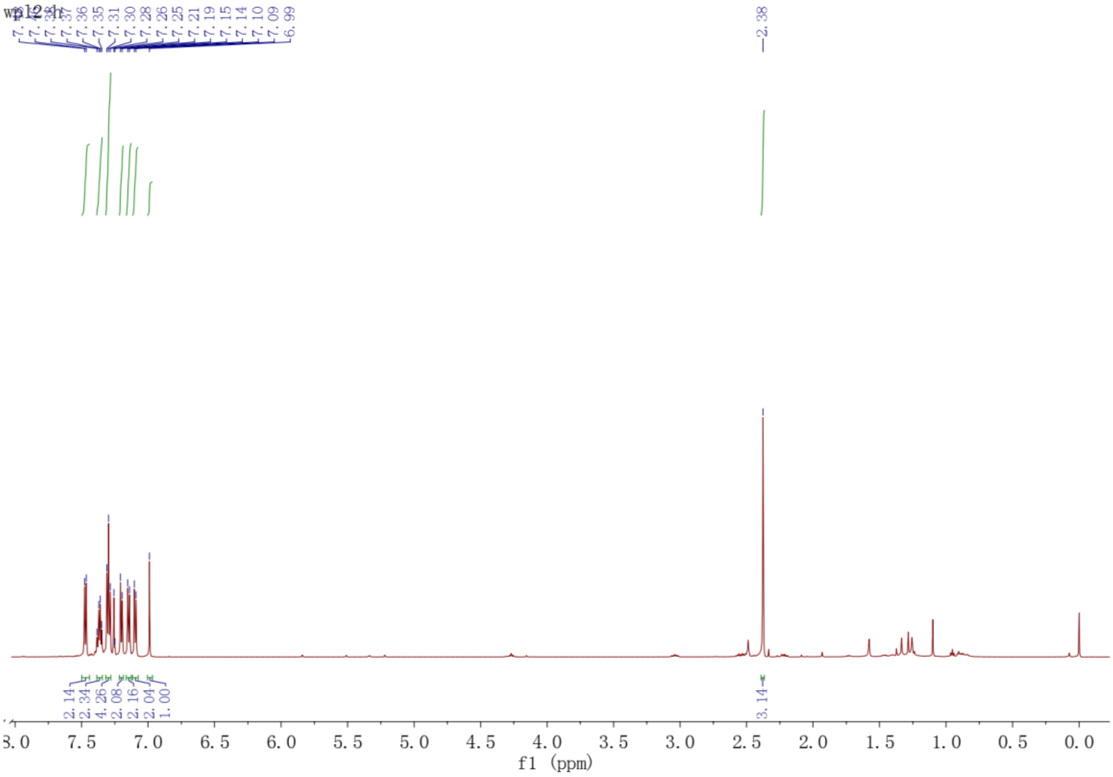
wp12-h
7.37
7.36
7.35
7.31
7.30
7.28
7.26
7.25
7.21
7.19
7.15
7.14
7.10
7.09
6.99
2.38
2.14
2.34
4.26
2.08
2.16
2.04
1.00
3.14
7.5
7.0
6.5
6.0
5.5
5.0
4.5
4.0
3.5
3.0
2.5
2.0
1.5
1.0
0.5
0.0
f1 (ppm)

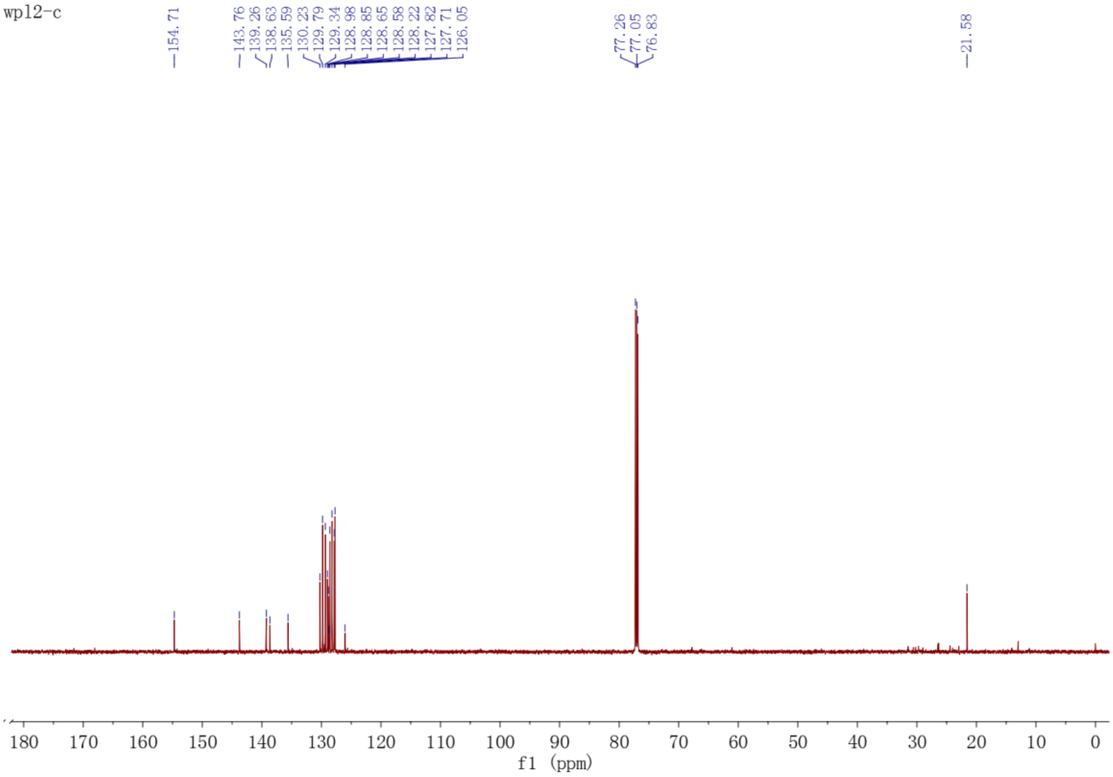
wp12-c
154.71
143.76
139.26
138.63
135.59
130.23
129.79
129.34
128.98
128.85
128.65
128.58
128.22
127.82
127.71
126.05
77.26
77.05
76.83
21.58
180
170
160
150
140
130
120
110
100
90
80
70
60
50
40
30
20
10
0
f1 (ppm)
